# The convergent neuroscience of Christian prayer and attachment relationships in the context of mental health: a systematic review

**DOI:** 10.3389/fpsyg.2025.1569514

**Published:** 2025-06-18

**Authors:** Egbert Haverkamp, Erik Olsman, Branislava Ćurčić-Blake, Víctor Vila Ramírez, André Aleman, Johannes C. F. Ket, Hanneke Schaap-Jonker

**Affiliations:** ^1^Community and Care, Protestant Theological University, Utrecht, Netherlands; ^2^Cognitive Neuroscience Center, University Medical Center Groningen, Groningen, Netherlands; ^3^Department of Clinical Neuropsychology, Faculty of Behavioural and Social Sciences, University of Groningen, Groningen, Netherlands; ^4^Department of Psychology, Universitat de les Illes Balears, Palma, Mallorca, Spain; ^5^Department of Neuropsychology and Psychopharmacology, Faculty of Psychology and Neuroscience, Maastricht University, Maastricht, Netherlands; ^6^Medical Library, Vrije Universiteit, Amsterdam, Netherlands; ^7^Faculty of Social Sciences and Humanities, School of Religion and Theology, Vrije Universiteit, Amsterdam, Netherlands; ^8^Centre for Research and Innovation in Christian Mental Healthcare, Eleos/De Hoop ggz, Hoevelaken, Netherlands

**Keywords:** adult attachment, Christianity, mental health, neuroimaging, prayer, psychotherapy, religion, social neuroscience

## Abstract

**Background:**

It has become increasingly accepted within psychotherapy to incorporate various forms of spirituality and religiosity to address the rising prevalence of mental health issues. This is well-founded, as a growing number of findings report benefits of spiritual practices for individuals experiencing depression, anxiety, and stress. However, science-based guidelines on how to embed spiritual practices in therapeutic interventions have not been developed, as the mechanisms by which human cognition, spirituality, and mental health interact—positively or negatively—remain largely unknown. Considering one of the most widely practiced religious behaviors worldwide, prayer, it is posited that the experience of interacting with God is psychologically comparable to human attachment bonds that are strongly associated with mental health.

**Method:**

This systematic review assesses the attachment to God hypothesis by providing an overview of the neural regions implicated in Christian prayer and attachment relationships, exploring their potential convergence. A systematic search was conducted in eight databases, resulting in 44 included records that examine brain activity during prayer or the activation of the attachment system in adults.

**Results:**

Evidence was found for convergence between prayer and neural correlates associated with the mentalizing module of attachment, comprising the default mode network (DMN) and areas associated with theory of mind (ToM), both related to social cognition. No significant differences were observed between prayer and attachment in regions connected to the approach and emotion (self-)regulation modules of attachment, whereas findings diverged for the aversion module of attachment, particularly in the insula.

**Discussion:**

The findings highlight shared cognitive and affective dimensions of attachment and prayer. Future research is warranted to identify whether neural patterns observed in different attachment styles coincide with distinct neural patterns of (Christian) prayer, so that both positive and negative effects of prayer can be better understood and integrated into psychotherapy.

**Systematic review registration:**

https://doi.org/10.17605/OSF.IO/HYZPN.

## Introduction

Whether through the rise of social media, the lack of deep and fulfilling social relationships in individualistic cultures, the expected global doubling of the elderly population by 2050, the lingering effects of a worldwide pandemic, or all of the above, there has been a dramatic growth in mental health problems in recent years, especially among youth (Eilert and Buchheim, 2023; Vadivel et al., 2021; Vos et al., 2016; Ghafari et al., 2022; Søvold et al., 2021; Jalali et al., 2024; Weigle and Shafi, 2024; Hodge and Gebler-Wolfe, 2022). As a consequence, psychotherapy has turned to incorporating mindfulness-based therapy to alleviate the increasing burden on mental healthcare workers, recognizing the need for cost-efficient preventive measures in clinical and non-clinical populations (Gkintoni et al., 2025; Galante et al., 2021; Goyal et al., 2014). Several meta-analyses have indeed found secular mindfulness-based therapies to be associated with decreased anxiety, depression, and stress symptoms, thereby addressing some of the most prevalent mental health disorders worldwide (Vos et al., 2016; Gkintoni et al., 2025; De Filippi et al., 2022; Kaisti et al., 2024). Notably, some studies indicate that spiritual forms of meditation, such as prayer, may have a similar or even greater influence on mental health, either positive or negative (Wachholtz and Pargament, 2005; Lucchetti et al., 2021). Although incorporating religion and spirituality into psychotherapy has been shown to improve treatment outcomes, psychotherapists often lack knowledge of the tenets of particular faith traditions, let alone their potential positive and negative side effects (Captari et al., 2018, 2022; Currier et al., 2019; Pargament, 2007; Cook, 2011). Hence, the statement issued by the WPO and WHO on integrating religiosity with psychotherapy cannot yet be fully realized, as the mechanisms by which different spiritual and religious practices interact with mental health remain largely unknown (Lucchetti et al., 2021; Moreira-Almeida et al., 2016).

Therefore, researchers from different backgrounds have called for the investigation of explanatory mechanisms through neuroimaging techniques (Galante et al., 2021; Pargament et al., 2004). However, this task is considerably complicated by the wide variety of spiritual practices supported by distinct underlying neural networks (Schjoedt et al., 2009; Newberg, 2014; James, 1902). Prior studies have attempted to unify these findings, for example, by differentially weighting interoceptive and exteroceptive stimuli within a broader framework of predictive processing (van Elk and Aleman, 2017). Although very useful in its own right, a more detailed account of the different functions and neural substrates of religious experiences is warranted, especially in relation to mental health (Thomas and Barbato, 2020; Nowicki et al., 2023).

When considering prayer, a central practice in religious traditions across the globe, it was found that meditative and colloquial prayer types positively correlated with existential wellbeing and happiness, whereas a negative association was detected between prayer and anxiety as well as depression in several studies (Winkeljohn Black et al., 2017; Black et al., 2015; Francis et al., 2008; Hebert et al., 2007; Koenig, 2007; Meisenhelder and Chandler, 2000, 2002; Anderson and Nunnelley, 2016; Schaap-Jonker, 2020; Chen and VanderWeele, 2018). Even though these effects could not be replicated in monotheistic religions other than Christianity, it needs to be explored to what degree these findings reflect sociological factors, such as being the majority religion in a country (Kobayashi et al., 2020). Contrarily, other studies found a mixed or negative association between prayer and mental health, also depending on the type of prayer (Upenieks, 2023; Froese et al., 2024; Braam et al., 2007; Newman et al., 2023). Regardless of the direction of the results, the effects in most studies were adjusted for prosocial factors such as social support or religious attendance, indicating that prayer itself affects mental health, as was also evident for mindfulness-based therapy (Anderson and Nunnelley, 2016; Froese et al., 2024; Tix and Frazier, 1998; Weber and Pargament, 2014; Ellison et al., 2014).

In search of an explanation for these effects, it has been suggested that religions provide a coping mechanism during hardships, for example, by allowing for the maintenance of a ‘just-world' view in adversity, or by attributing circumstances to God (Pargament et al., 2004; Weber and Pargament, 2014; Szałachowski and Tuszyńska-Bogucka, 2021; Breslin and Lewis, 2008; Graça and Brandão, 2024; Pargament et al., 2005; Pargament and Hahn, 1986; Spilka et al., 1985). Accordingly, these factors influence outcomes during major life stressors, thereby impacting the mental health of individuals (Thomas and Barbato, 2020; Nowicki et al., 2023). Notwithstanding the clear contribution of such concepts in attributing meaning to particular religious experiences, they do not constitute what others have identified as the essence of these experiences, such as the “feeling of absolute dependence” or “the willingness to surrender,” perhaps with the exception of “seeking spiritual support” through prayer (Seibert, 2023; Pargament, 1997). Even then, these descriptions do not directly lead to hypotheses about how perceived support is connected with cognitive functions or which empirically assessable neurobiological networks may sustain it.

On the contrary, verifiable insights may be attained through attachment theory, which originated as a psychological theory but has been validated and further developed through neuroimaging techniques in recent decades (Bowlby, 1982; White L. K. et al., 2023; Long et al., 2020; Vrticka, 2017; Vrtička et al., 2012; Labek et al., 2016). In brief, this theory posits that humans are biologically predisposed to seek close proximity to their primary caregivers to attain safety (Bowlby, 1982; Main, 1991). Over time, the availability and emotional sensitivity of caregivers lead to both conscious and unconscious expectations about the ability to rely on others, as well as a positive or negative self-image, depending on whether an individual feels worthy of attention and care (Fonagy and Luyten, 2009; Fonagy et al., 2014). These so-called internal working models were initially thought to remain fixed throughout life. However, recent studies indicate that they are somewhat malleable through psychotherapy, potentially becoming more secure, avoidant, preoccupied, or unresolved (Taylor et al., 2015; Levy et al., 2006; Buchheim et al., 2012a).

Two strands of literature have since developed to classify attachment styles. The first employs implicit measures, including the strange situation procedure (SSP) in children and the adult attachment interview (AAI) or the subsequent adult attachment projective (AAP). In keeping with attachment theory, these measures focus on mental representations and unconscious defensive processes, with the AAP and SSP directly activating the attachment system (Buchheim et al., 2003; George and West, 2001; Bakermans-Kranenburg and van IJzendoorn, 2009; Solomon and George, 2008; Ainsworth et al., 2015). As these methods take considerable time and require extensive training, self-report questionnaires in the tradition of Mikulincer and Shaver were later developed (Mikulincer and Shaver, 2003). Despite their convenience, this method cannot detect unconscious processes, distorts the distinction between preoccupied and unresolved classifications, allows avoidant individuals to classify themselves as securely attached, and is associated with different neural representations compared to implicit measures (Solomon and George, 2008; Yaseen et al., 2016; Solomon and George, 1999; George and Solomon, 1996; Roisman et al., 2007; Ravitz et al., 2010). Nevertheless, self-report outcomes may reflect explicit and conscious attachment representations, supported by the executive frontal network (Yaseen et al., 2016). Although attachment relationships initially require the physical proximity of primary caregivers to return to emotional homeostasis, humans gradually replace this proximity with an internal working model of the parental figure (Fonagy et al., 2014). Referring to this representation enables children and adults to explore from an internalized secure base (Solomon and George, 2008; George and Solomon, 1996). This process is readily observed when growing children venture further without relying on the continued presence of a parent. The degree to which early attachment needs were met also influences how individuals learn to mirror and infer the mental states of others, commonly referred to as mentalizing (Fonagy and Luyten, 2009; Fonagy et al., 2023; Frith and Frith, 2012; Granqvist and Kirkpatrick, 2013; Norenzayan et al., 2012).

These internal working models, implied in mentalizing, may also be recruited during prayer (Fonagy et al., 2023; Norenzayan et al., 2012; Schaap-Jonker and Corveleyn, 2014). From a developmental perspective, Bowlby argued that individuals form secondary attachment relationships, potentially even with larger entities such as political parties or specific groups of people (Bowlby, 1980). In line with this view, it has been theorized that prayer can similarly be understood from an attachment perspective, with God as a “substitute attachment figure” (Granqvist and Kirkpatrick, 2013; Cherniak et al., 2021; Granqvist, 2006). So far, this hypothesis has been supported by self-report questionnaires, revealing an association between secure attachment to God and improved mental health, and a reverse correlation for insecure attachment to God (Ellison et al., 2014; Ghobary Bonab et al., 2013; Leman et al., 2018; Counted, 2016; Virto-Farfan et al., 2023). Nonetheless, when comparing representations of God with those of important others, the results were mixed, with some findings more supportive of *compensation*, whereby God functions as a substitute attachment figure for those with insecure attachment to primary caregivers, whereas other studies indicate that perceived attachment to God *corresponds* with implicit representations of self and others (Cherniak et al., 2021; Hall et al., 2009; Holmes and Slade, 2019). Attachment to God may explicitly compensate for a lack of parental care, while an implicit connection to insecurity remains (Schaap-Jonker and Corveleyn, 2014; Hall et al., 2009; Stulp et al., 2021).

In order to assess how the relationships between implicit and explicit attachment to God may function, as well as to verify whether prayer to God can be understood through attachment theory, neuroscience could provide valuable insights (van Elk and Aleman, 2017). Previous neuroimaging studies have shown the recruitment of Theory of Mind (ToM) networks during prayer, as well as increased activity in the default mode network (DMN), mirroring the neural activations observed in the context of friendship (Schjoedt et al., 2009; Neubauer, 2014; Li et al., 2014; McNamara, 2009). Both networks overlap with the broader framework of social cognition, which may indicate a shared mechanism between attachment and prayer that will be further explored in this article. In the neuroscience of attachment, four modules of attachment were identified in prior research, comprising a large number of neural areas associated with attachment behaviors (Coan, 2008). These modules include the approach and aversion, as well as the emotion self-regulation and mentalizing modules (Long et al., 2020; Vrticka, 2017; White et al., 2020). As mentioned in the preregistration, we expected both prayer and attachment to require mentalizing behaviors (Norenzayan et al., 2012; Schaap-Jonker and Corveleyn, 2014). Moreover, a slight convergence with self-regulation and the approach or aversion modules may be observed during prayer, possibly reflecting mental approach or avoidance depending on God-image and the positive or negative coping strategies employed during prayer (Pargament et al., 2004; Bradshaw et al., 2010; Aletti, 2005). In brief, this systematic review evaluates whether perceived interaction with God can accurately be described as an attachment relationship from a neuroscientific perspective. In doing so, we seek to understand how religious beliefs are interwoven with everyday human cognition, to provide a pathway for future research and ultimately integrate religious belief within psychotherapy to enhance treatment outcomes.

## Methods

This systematic review was conducted in accordance with the PRISMA recommendations (Page et al., 2021). The screening protocol, including inclusion and exclusion criteria, was pre-registered in the Open Science Foundation (Haverkamp, 2024). Subsequently, two authors independently carried out the first and second screening phases in Rayyan (Ouzzani et al., 2016). Due to the heterogeneity of the included articles and methodologies employed, we used a qualitative synthesis to integrate the results. The findings are described in six separate sections: 1. Implicit measures in healthy samples, 2. Implicit measures with comorbidity, 3. Explicit measures in healthy samples, 4. Structural neurobiological findings of attachment (VBM), 5. Structural neurobiological findings of attachment (DTI), 6. Neural correlates of Christian prayer. The discussion focuses on the main effects of prayer and attachment, and evaluates the neural differences between attachment styles that may impact the neuroscience of prayer. Finally, the convergence and divergence between the neuroscience of prayer and attachment relationships are assessed.

### Database searches

A comprehensive search was conducted in the following databases: Elsevier/Scopus, OVIDMedline, Ebsco/ATLA Religion, Ebsco/CINAHL, Ebsco/APA PsycINFO, Ebsco/Psychological and Behavioral Sciences Collection, PubPsych.eu, and Clarivate Analytics/Web of Science Core Collection, from inception to March 25, 2024, in collaboration with a medical information specialist (JCFK). The search included controlled and free text terms for synonyms of “attachment” or “prayer” and “MRI” or “MEG”, and separately “object relation” and “MRI” or “MEG”. The search was performed without restrictions on methodology, date, or language. The full search strategies can be found in the Supplementary material. Duplicate articles were removed by a medical information specialist (JCFK) using Endnote X20.0.1 (Clarivate^tm^), following the Amsterdam Efficient Deduplication (AED) method (Otten et al., 2019) and the Bramer method (Bramer et al., 2016). Systematic reviews deemed relevant to our endeavor were examined for cross-references.

### Screening procedure

The inclusion and exclusion criteria were specified in the research protocol beforehand and uploaded to the Open Science Foundation (Haverkamp, 2024). The PICOS criteria for the two types of studies are mentioned in Tables 1, 2. We decided to include studies on prayer in other (monotheistic) faith traditions as well, as specified in the screening protocol uploaded to OSF. After the screening, we determined whether including them would be necessary based on the number of records related to Christian prayer. Three studies were identified (Baykara et al., 2023; AlMahrouqi and Mostafa, 2023; Perez-Diaz et al., 2023); however, we chose not to incorporate the results in the main analysis for two reasons. First, the number of studies on Christian prayer exceeded our expectations. More importantly, including other faith traditions could potentially bias the effects through faith diffusion and is based on the unwarranted assumption that different religious and spiritual practices are similarly represented in the brain (Winkeljohn Black et al., 2017; Newberg, 2018). Prior to the screening procedures, we expanded the inclusion criteria to include participants suffering from anxiety disorder, depressive disorder without manic episodes, borderline personality disorder, narcissistic personality disorder, and prolonged grief disorder, as these conditions are strongly connected with insecure attachment (Galynker et al., 2012; Buchheim et al., 2012b; Bettmann and Jasperson, 2010; Cascio et al., 2013; Buchheim et al., 2008). To distinguish specific effects for each attachment style, we included attachment studies that reported both main effects and differences across attachment styles, as prayer studies were expected to reveal a more regular distribution (Bakermans-Kranenburg and van IJzendoorn, 2009). Moreover, we excluded articles where conditions evoked the affiliative, parental, or romantic/sexual system, since attachment theory, along with neuroscientific results, indicates that these behavioral systems are characterized by different goals and functions. This distinction is further supported by their distinct neural substrates (Bowlby, 1982; Bartels and Zeki, 2000; Wolfe et al., 2018; Laurita et al., 2019; Hou et al., 2016; George and Solomon, 2008). Finally, we sought to isolate the effects of adult attachment to contribute to our discussion within the wider neuroscientific literature on attachment, predominantly conducted with adults, as attachment in youth may be represented differently in the brain (Takamura et al., 2016, 2015).

**Table 1:** PICOS criteria for inclusion and exclusion of attachment studies.

**Population**	**Mentally healthy adults of various backgrounds and attachment styles**
**Intervention**	Attachment style assessment through self-report measures or interviews. Participants are exposed to a stimulus that activates the attachment system while being scanned through a neuroimaging technique to explore the neural correlates of attachment
**Comparator (control group)**	Activity in the attachment condition was compared to a non-attachment control condition
**Outcomes of interest**	Neural activation during activation of the attachment system in human beings in comparison to a non-attachment control condition. Data on the activation pattern of neural areas in included articles should be obtained through neuroimaging methods similar to the techniques mentioned in the extended screening protocol, uploaded in OSF
**Study type**	Critically appraised studies that assess the neural correlates of attachment relationships or object relations through neuroimaging techniques

**Table 2:** PICOS criteria for inclusion and exclusion of prayer studies.

**Population**	**Mentally healthy adults of various backgrounds and attachment styles**
**Intervention**	Included prayer studies should expose participants to any form of colloquial, meditative, and/or improvised Christian prayer performed by adults who are praying alone. Studies on other forms of Christian prayer (petitionary or ritual prayer) might be analyzed separately
**Comparator (control group)**	A non-prayer control condition. Examples are reading a poem out loud or making wishes to Santa Claus. At least, the prayer-exposure should be controlled for by baseline activity
**Outcomes of interest**	Neural correlates of Christian prayer in comparison to a control condition. Data should be obtained through neuroimaging methods
**Study type**	Critically appraised studies that assessed the neural correlates of Christian prayer through neuroimaging techniques

**Figure 1:**
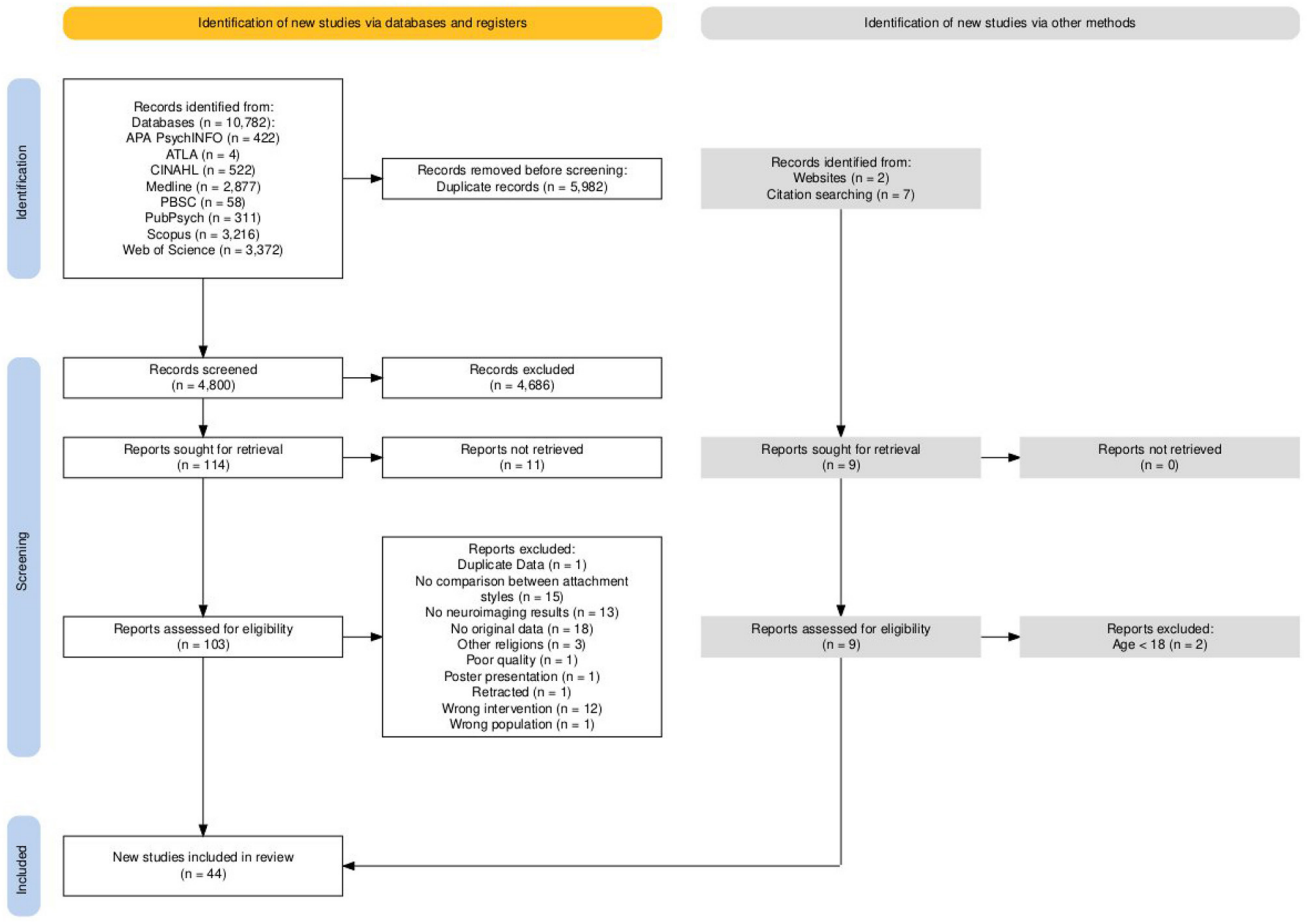
PRISMA flowchart of included and excluded studies during screening protocol.

For the flowchart, see Figure 1 (Haddaway et al., 2022). The records identified via citation chaining and a hand search, as well as articles included in the screening stages and reasons for exclusion, are reported here. Differences between authors regarding the inclusion and exclusion of records were resolved through discussion or by consulting a third party. For evaluation of the screening process, see Supplementary material 1.

### Data extraction

Quality assessment and data extraction were performed by the first author. For the adapted version of the JBI critical appraisal tool for analytical cross-sectional studies, see Supplementary material 2. Assessment was performed based on the following criteria: 1. Specified criteria for inclusion, 2. Subject settings, 3. Validity of exposure stimulus, 4. Objective criteria for measurement of the condition, 5. Identification of confounding factors, 6. Measurement of outcomes, and 7. Quality of statistical analysis. Quality assessment yielded satisfactory results overall, with 40 out of 44 studies scoring 7/10 or higher, and four studies scoring 6/10. One study was excluded. The greatest potential source of bias was the use of small sample sizes. This may be common in neuroimaging studies due to the high costs of neuroimaging techniques, but it does warrant caution in extending results to larger populations. Furthermore, most of the findings obtained were based on samples that consisted largely of females, indicating that the results should not be generalized to male populations. Study details are listed in Supplementary Tables 2–7.

## Results

The characteristics of included studies, including demographics, populations, and neuroimaging methods, are as follows: 31 records focused on the neuroscience of attachment. They were conducted in Europe (*N* = 19), North America (*N* = 9), and Asia, China (*N* = 3). Functional studies of attachment involved 543 participants (73% female). Among these studies, articles employing implicit attachment measures demonstrated an even greater imbalance (85% female). Structural neuroimaging outcomes of attachment were obtained with a near-equal representation of males and females (*N* = 1,021). Most functional and structural studies employed (f)MRI, with exceptions including two studies using EEG. Furthermore, 13 studies assessed prayer through neuroimaging techniques: they were conducted in Europe (*N* = 7) and North America (*N* = 6), involving 234 participants (63% female). Most results were obtained through fMRI, apart from three EEG studies, two studies that utilized PET, and one study conducted with sMRI.

To obtain an overview of data on the neuroscience of attachment and prayer in hypothesized areas, a graph was created highlighting the neural regions identified in prior studies (Long et al., 2020; White et al., 2020). None of the prayer-related studies accounted for the effects of attachment styles. Thus, at present, convergence can only be investigated through an analysis of the main effects. Therefore, for each neural area, we calculated the percentage of participants exhibiting a significant positive effect by dividing the number of participants in whom activation was observed by the total number assessed, multiplied by 100. Importantly, although our results reflect individual participant data, they were not weighted for effect size as is typically done in meta-analyses, since variations in methodologies employed to activate the attachment system limit the applicability of standard meta-analytic approaches. Moreover, we entered the main effects of attachment obtained using functional neuroimaging techniques as specified in Tables 3–5, as there is no control condition to isolate the main effects of attachment in structural imaging. Finally, the main effects of attachment included in this table were derived from results obtained using negative stimuli, such as the unpleasant stimuli employed by Vrtička et al. (2012). This approach was chosen because the attachment system is primarily activated in adverse circumstances—such as solitude, separation, and abuse—wherein compensatory behavioral and physiological responses are required to restore emotional homeostasis (White et al., 2020; Buchheim et al., 2006).

**Table 3:** Attachment style implicitly assessed in healthy populations.

**References**	**Adults sample (female)**	**Imaging technique**	**Field of view**	**Attachment assessment instrument**	**Attachment styles assessed**	**Stimuli**	**Control**	**Main findings: neural correlates of attachment (R: right, L: left)**	**Corrected for multiple comparisons**
Buchheim et al. (2006)	11 (F = 11)	fMRI	Whole brain	AAP interview	**F**, secure; **Ds**, dismissing; **E**, preoccupied; **U**, unresolved newline F/Ds/E: 6 U: 5	AAP	Increasing attachment system effect	**ME**, main effects of attachment stimuli including all classifications; **CSAS**, correlates for separate attachment styles; **DBAS**, differences between attachment styles **ME:** L SFG, L MFG, R/L precentral gyrus, R/L MSTG, R/L occipital cortex, R/L caudate nucleus, globus pallidus, R ACC, L/R cerebellar hemisphere **DBAS, unresolved** **>** **resolved:** R IFG, L STG, L caudate nucleus, R/L AMY **DBAS:** unresolved > resolved for all AAP stimuli: R precentral gyrus, IFG, L STG, R occipital cortex **DBAS:** unresolved > resolved for monadic AAP stimuli: ACC, and parahippocampal gyrus for dyadic AAP stimuli	Uncorrected
Lemche et al. (2006)	12 (F = 5)	fMRI	Whole brain	Reaction time difference to stress primes	Secure: 7 Insecure: 5	Subliminal prime sentences of unpleasant attachment experiences based on the design of Maier et al. (2004)	Neutral prime sentences	**ME:** R MTG, L DLPFC, R VLPFC, L STG, R IPL, L MOG, L INS, L putamen, R cuneus, R/L AMY **CSAS, insecurity:** L VLPFC, L IPC, L MTG L STG, R DACC R/L AMY	*P* < 0,005 Cluster-wise probability threshold
Petrowski et al. (2019)	38 (F = 28)	fMRI	Whole Brain	AAP interview	F: 14 Ds/E: 15 U: 9	Faces of romantic partners and parents	Viewing unfamiliar faces	**ME:** R/L MFG, L SFG, R IFG, R precentral/postcentral gyrus, SMA, R/L MTG, R/L supramarginal gyrus, R/L INS, R rolandic operculum, L precuneus, R/L ACC, L PCC, R/L cingulate gyrus, R/L thalamus, R/L cerebellum, R cerebellar vermis, L caudate nucleus, L hippocampus, L parahippocampal gyrus, L AMY **DBAS, secure** **>** **disorganized:** L IPL, L STG, L INS, R ACC, R MCC, L cerebellum/cerebellar vermis **DBAS, insecure** **>** **disorganized:** R IFG, L IPL, R putamen, R MCC **DBAS, secure** **>** **insecure, insecure** **>** **secure, disorganized** **>** **secure and disorganized** **>** **insecure:** no suprathreshold voxels	P <0,001 Cluster-wise probability threshold, uncorrected at voxel level
Yaseen et al. (2016)	28 (F = 28)	fMRI	Whole brain	AAI interview	F: 15 Ds: 4 E: 4 U: 3	Valence and salience viewing of pictures of mother	Neutral viewing pictures of mother	**ME in salience contrast:** increased activity in R/L thalamostriatal system and PCC, deactivations in the R/L OPFC **ME in valence contrast:** increased activity in R/L thalamostriatal system, PCC and L INS **CSAS, security:** increased activity in R parahippocampal gyrus, R PCC, R FG, and deactivations in the R/L cuneus **CSAS, dismissiveness:** increased activity in R cuneus, L lingual gyrus, R/L thalamus, deactivations in: L MFG, R/L temporal lobe white matter tracts, R ACC, parahippocampal gyri, CC	P <0,005 Cluster-wise probability threshold

AMY, amygdala; CC, corpus callosum; CSAS, correlates for separate attachment styles. DACC, dorsal anterior cingulate cortex; DBAS, differences between attachment styles; DLPFC, dorsolateral prefrontal cortex; Ds, dismissing; E, preoccupied; F, secure; FG, fusiform gyrus; fMRI, functional magnetic resonance imaging; IFG, inferior frontal gyrus; INS, insula; IPC, inferior parietal cortex; IPL, inferior parietal lobule; ME, main effects; MFG, middle frontal gyrus; MOG, middle occipital gyrus; MSTG, middle superior temporal gyrus; MTG, middle temporal gyrus; OPFC, orbital prefrontal cortex; PCC, posterior cingulate cortex; SFG, superior frontal gyrus; SMA, supplementary motor area; STG, superior temporal gyrus; U, unresolved; VLPFC, ventrolateral prefrontal cortex.

**Table 4:** Attachment implicitly assessed in samples with comorbidities.

**References**	**Sample size (females) [comorbidity/ healthy controls (HC)]**	**Imaging technique**	**Field of view**	**Attachment assessment instrument**	**Attachment styles**	**Stimuli**	**Control**	**Main findings: neural correlates of attachment (R: right, L: left)**	**Corrected for multiple comparisons**
Bernheim et al. (2022)	26 (F = 26) BPD 26	fMRI	Whole brain	AAP interview	**F**, Secure; **Ds**, Dismissing; **E**, preoccupied; **U**, unresolved For monadic AAP stimuli: F/Ds/E: 8 U: 18 For dyadic AAP stimuli: F/Ds/E: 22 U: 4	AAP with personalized sentences	AAP with neutral sentences	**ME**, main effects of attachment stimuli including all classifications; **CSAS**, correlates for separate attachment styles; **DBAS**, differences between attachment styles newline **ME:** bilateral fronto-temporal and occipital activation **DBAS, more unresolved BPD** **>** **resolved for monadic AAP stimuli:** R STS, AMCC, L thalamus, R/L AINS, R AMY **DBAS, more unresolved BPD patients** **>** **resolved participants, for monadic** **>** **dyadic AAP stimuli:** L VMPFC, DMCC/PCC, L AINS, R AMY **DBAS, more unresolved BPD** **>** **resolved for dyadic AAP stimuli:** No suprathreshold voxels	P <0,05 cluster-wise FWE corrected
Buchheim et al. (2008)	30 (F = 30) HC 17 BDP 11	fMRI	Whole brain	AAP and AAI interview	HC F/Ds/E: 10 HC U: 7 BPD U: 11	AAP stimuli (pre-speech + narrative)	Baseline	**ME in controls for all AAP stimuli:** L DLPFC, L VLPFC, R SFG, L PREC, L precentral gyrus, R SPL, R parahippocampal gyrus, R/L OC **ME in BPD patients for all AAP stimuli:** R VLPFC, L SFG, R PREC, R/L precentral gyrus, R cuneus, R/L OC, R/L cerebellum **DBAS, ranking from lowest to highest ACC activity in response to monadic and dyadic AAP stimuli**: resolved controls, unresolved controls, unresolved BPD patients **DBAS, ranking from lowest to highest parahippocampal gyrus activity in response to monadic and dyadic AAP stimuli:** unresolved BPD patients, resolved controls, unresolved controls **DBAS, ranking from lowest to highest STG activity in response to dyadic viewing AAP stimuli:** resolved and unresolved controls (equal), unresolved BPD patients	*P* < 0,001 Voxel-wise probability threshold and a *P* < 0,05 Cluster-wise probability threshold
Buchheim et al. (2012a)	36 (F/M ratio not significantly different across groups) HC 17 MDD 16	fMRI	Whole brain	AAP interview	Although not specified, Most likely similar to (Buchheim et al., 2012b) below	AAP with personalized sentences	AAP with irrelevant sentences	**DBAS, more unresolved MDD patients** **>** **resolved participants:** increased activity in AMPFC, L MTG, VACC, L AMY, AHC	Uncorrected
Buchheim et al. (2012b)	36 (F/M ratio not significantly different across groups) HC 17 MDD 16	fMRI	Whole brain	AAP interview	HC F/Ds/E: 17 HC U: 3 Patients F/Ds/E: 9 Patients U: 11 newline After 15 months of therapy, only four of the remaining 18 patients were judged unresolved	AAP with personalized sentences	AAP with irrelevant sentences	**DBAS, more unresolved MDD patients** **>** **resolved participants:** AMPFC, L MTG, VACC, L AMY, AHC	Uncorrected
Buchheim et al. (2013)	1 (F = 1) a female with narcissistic traits and dysthymia	fMRI	Whole brain	AAP interview	U: 1	AAP with personalized sentences	AAP with neutral sentences	**ME summary:** VLPFC, DLPFC, perigenual portion of MPFC, PCC, PREC, MTG, anterior tip of the ITG, occipital/calcarine cortex. **ME in Table 3 of reported study:** L Inf. frontal orbital, L. Inf. temporal, L medial temporal, L medial frontal orbital, L medial frontal orbital, R superior occipital, R calcarine, L SMA, L superior frontal, R precentral, R inferior frontal operculum, R medial temporal, right superior temporal, L medial cingulum, L inferior parietal area	P <0,017 cluster-wise corrected via RFT for posterior cingulate, other levels of significance are specified in Table 3 in the article
Buchheim et al. (2016)	28 (F = 28) HC 17 BPD 11	fMRI	Whole brain	AAP interview	HC F/Ds/E: 10 HC U: 7 BPD U: 11	AAP	Increasing attachment system effect	**ME for all controls:** DLPFC, MPFC, AMY **CSAS, resolved controls:** STS **CSAS, unresolved controls:** DLPFC, SFG, AMY **CSAS, unresolved BPD patients:** STS, CG, ACC, AMY **CSAS, unresolved BPD patients and controls:** STS, MPFC, AMY **DBAS, unresolved controls** **>** **resolved controls:** DLPFC, AMY **DBAS, resolved controls** **>** **unresolved BPD patients:** MPFC **DBAS, unresolved controls** **>** **unresolved patients:** DLPFC, MPFC	P <0,05 Cluster-wise FWE corrected
Flechsig et al. (2023)	41 (F = 41) HC 23 BPD 18	fMRI	Whole brain	AAP interview	AAP score ascribed ranging from 1-4 for each attachment in order of appearance (F, Ds, E, U), with lower scores in the BPD group reflecting insecure attachment	AAP with personalized sentences	AAP with neutral sentences	**DBAS, more unresolved BPD patients** **>** **more resolved controls:** L AMY, AMCC **DBAS, more unresolved BPD patients** **>** **more resolved controls after one year of DBT:** no suprathreshold voxels	Two-sided P <0,0032 voxel-wise corrected, method not specified
Galynker et al. (2012)	28 (F = 28) HC 14 MDD 14	fMRI	Whole brain	AAI interview	AAI coherence of mind score from 1-9 with 6-9 representing secure, 1-3 representing insecure attachment and 4-5 indeterminate	Viewing photographs of mother	Viewing photographs of close female friend or strangers	**ME, Mother** **>** **Stranger:** R/L CG, L FMC, R/L LOC, AG, R PP, R IFG, R IC, R TP, L FP, L FOC, R/L TOFC, L TFC, L MTG, and deactivations in L FP, R/L SMG, R Postcentral Gyrus, R Precentral Gyrus, R LOC in the superior division, R MFG, R IC, L PG **ME, Mother** **>** **Friend:** R MFG, L PG, R IFG, deactivations in the R Frontal Pole, L Lateral Ventricular Frontal Pole **CCAS, insecure attachment Mother** **>** **Stranger contrast:** L SFG, L PCG, R/L MFG, L SPL, R/L SMG, R LOC, L FOC, R/L FP, R/L Thalamus, R/L Caudate, R IC, deactivations in the R/L Posterior CG, L PREC, R/L AG, R PP, R/L FP, R SMG, L MFG, L LG **CCAS, insecure attachment Mother** **>** **Friend contrast:** L ventral caudate, R medial thalamus, deactivations in the L occipital fusiform gyrus, R PREC, R intracalcarine cortex	Absolute Z-score higher than 2,32 (P <0,01) Voxel-wise corrected P <0,05 Cluster-wise FWE corrected
Zhang et al. (2011)	28 (F = 28) HC 14 MDD 14	fMRI	Whole Brain and a GLM analysis to identify ROI: L anterior PCG	AAI interview	AAI coherence of mind score from 1-9 with 6-9 representing secure, 1-3 representing insecure attachment and 4-5 indeterminate	Viewing photographs of mother	Viewing images of a female friend and female strangers	**DBAS, more secure attachment in healthy controls** **>** **more insecure attachment in patients with MDD for mother** **>** **stranger contrast:** deactivations in the APCG	Uncorrected

AAI, adult attachment interview; AAP, adult attachment projective; ACC, anterior cingulate cortex; AG, angular gyrus; AINS, anterior insula; AMCC, anterior midcingulate cortex; AMY, amygdala; APCG, anterior paracingulate gyrus; BPD, borderline personality disorder; CG, cingulate gyrus; CSAS, correlates for separate attachment styles; DBAS, differences between attachment styles; DBT, dialectic behavioral therapy; DMCC, dorsal medial cingulate cortex; Ds, dismissing; E, preoccupied; F, secure; FMC, frontal medial cortex; fMRI, functional magnetic resonance imaging; FOC, frontal orbital cortex; FP, frontal pole; FWE, family-wise error; HC, healthy controls/hippocampus; IC, insular cortex; ITG, inferior temporal gyrus; LG, lingual gyrus; LOC, lateral occipital cortex; MDD, major depression disorder; ME, main effects; MFG, middle frontal gyrus; MTG, middle temporal gyrus; OC, occipital cortex; PG, postcentral gyrus; PCG, paracingulate gyrus; PP, planum polare; PREC, precuneus; RFT, random field theory; ROI, region of interest; SFG, superior frontal gyrus; SMG, supramarginal gyrus; SPL, superior parietal lobule; STS, superior temporal sulcus; TFC, temporal fusiform cortex; TOFC, temporal occipital fusiform cortex; TP, temporal pole; U, unresolved; VACC, ventral anterior cingulate cortex.

**Table 5:** Attachment explicitly assessed in healthy populations.

**References**	**Sample size (female)**	**Imaging technique**	**Field of view**	**Attachment assessment instrument**	**Attachment styles**	**Stimuli**	**Control**	**Main findings: neural correlates of attachment (R, right; L, left)**	**Corrected for multiple comparisons**
Canterberry and Gillath (2013)	30 (F = 15)	fMRI	Whole brain	ECR	**S**, secure; **Av**, avoidant; **Ax**, anxious; **FA**, fearful-avoidant newline A regression analysis was conducted for avoidant and anxious attachment styles. Individual attachment classifications were not specified	(Sub)liminal attachment (in)security-related prime sentences. They modified the design of Murphy and Zajonc (1993)	(Sub)liminal implicit and explicit neutral primes	**ME**, main effects of attachment stimuli including all classifications; **CSAS:** correlates for separate attachment styles; **DBAS**, differences between attachment styles **ME during explicit security vs. neutral contrast:** MFG **ME during explicit security vs. explicit insecurity contrast:** R PG, L FFG, L MTG, L PHG, L MFG, R MTG, L cerebellum nodule, R/L cerebellum declive **ME during explicit insecurity** **>** **explicit neutral contrast:** L MTG, L STG, L IFG **ME during explicit insecurity** **>** **explicit security contrast:** L STG **CSAS, avoidance for explicit security** **>** **explicit neutral contrast:** R caudate, R/L AMY, L PHG, R/L STG, L precentral gyrus, R thalamus, L INS, L MTG **CSAS, avoidance for explicit security** **>** **explicit insecurity contrast:** L MTG, L STG, L IP, L PHG **CSAS, avoidance for implicit security** **>** **implicit insecurity contrast:** no suprathreshold voxels **CSAS, anxiety for explicit security** **>** **explicit neutral contrast:** No suprathreshold voxels **CSAS, anxiety for explicit security** **>** **explicit insecurity contrast:** L Paracentral Lobule, L CG, L IP **CSAS, anxiety for implicit security** **>** **implicit insecurity contrast:** R MFG, R SFG, R IFG	Uncorrected
Krause et al. (2016)	23 (F = 0)	fMRI	ROI: AMY, HC and DACC	ECR	A regression analysis was conducted for avoidant and anxious attachment styles. Individual attachment classifications were not specified	Listening to secure, preoccupied and dismissing speech	Baseline measurement	**ME for dismissing narrative listening** **>** **baseline:** increased functional connectivity between R/L DACC and R/L AMTG **CSAS, anxious attachment controlled for dismissing attachment:** increased functional connectivity between L DACC and R DLPFC (uncorrected, controlled for dismissing attachment) **CSAS, dismissing attachment controlled for anxious attachment:** increased functional connectivity between R DACC and AMTG, between R DACC and MPFC (uncorrected, controlled for dismissing attachment). Functional connectivity between DACC and hippocampus failed to show significance	P <0,05 Voxel-wise FDR corrected
Liu et al. (2017)	33 (F = 14)	fMRI	Whole brain	ECR	S: 16 (8 females) Av: 17 (6 Females)	Viewing images of positive romantic and parent–child bonding, and negative romantic and parent–child bonding scenes	Viewing images of neutral non-attachment scenes	**CSAS, secure attachment during engagement with negative emotional stimuli (but not positive emotional stimuli):** R FFG, R MOG **CSAS, avoidant attachment during attentional engagement with positive and negative emotional stimuli:** R/L FFG, R/L MOG **CSAS, avoidant attachment during attentional disengagement from positive (but not negative emotional stimuli):** R/L FFG, R/L MOG **DBAS, avoidant** **>** **secure attachment during attentional engagement with emotional stimuli:** R STG, R/L MOG, L MFG, SMA, CG	P <0,05 Cluster size of 20+ voxels, Cluster-wise FWE corrected
Nash et al. (2014)	56 (F = 38)	EEG	Scalp locations: Cz and FCz in the EEG 10-20 system	ECR	S: 21 Av: 8 Ax: 18 FA: 9 Analysis was conducted with scores of insecurely attached participants collapsed into one insecure category as opposed to security.	Before and after asking participants to think about feeling insecure, participants conducted a multi-source interference task.	NA	**ME for all participants:** Greater error-related negativity (ERN) signal observed at Cz and FCa after the insecurity threat **DBAS, insecure** **>** **secure participants:** increased signal at Cz and FCz after the insecurity threat	Not mentioned
Vrtička et al. (2012)	19 (F = 19)	fMRI	Whole brain and ROI for areas related to social vs. non-social stimuli	RQ	A regression analysis was conducted for both Av- and Ax-scores across all participants. Individual attachment classifications were not specified	Pleasant and unpleasant IAPS affective pictures during natural viewing, reappraisal, or suppression	Non-social IAPS pictures	**ME of social vs. non-social scenes pleasant and unpleasant scenes:** L/R AMY, MOFC, MPFC, PCC, PREC, R/L FFA, R/L FBA, L fusiform gyrus, R DLPFC, R/L TI, R/L PSTS/occipital, L occipital **CSAS, avoidance for social unpleasant** **>** **non-social unpleasant during natural viewing:** L LPFC, R/L DLPFC, R LPFC R/L DACC, R/L VACC **CSAS, anxiety for social pleasant** **>** **non-social pleasant during natural viewing:** L parahippocampus **CSAS, anxiety for social unpleasant** **>** **non-social unpleasant during natural viewing:** R AMY	P <0,05 Voxel-wise FDR-corrected
Rognoni et al. (2008)	39 (F = 20)	EEG	Scalp Locations: Fp1, Fp2, F7, F8, F3, F4, Fz, C3, C4, Cz, T3, T4, T5, T6, P3, P4, Pz, O1, and O2 in the EEG 10-20 system	RQ	S: 14 Av: 9 Ax: 9 FA: 7	Attachment related film content involving happiness, sadness or fear	Neutral film content	**DBAS, ranking from left asymmetry (approach behavior) to right asymmetry (withdrawal) during happy emotional scenes:** anxious, fearful-avoidant, aecure, avoidant **DBAS, ranking from left asymmetry (approach behavior) to right asymmetry (withdrawal) during fearful emotional scenes:** Avoidant, Secure, Fearful-Avoidant & Anxious **DBAS, ranking from left asymmetry (approach behavior) to right asymmetry (withdrawal) during sad emotional scenes:** avoidant, anxious, secure, fearful-avoidant	Bonferroni corrected
Yaseen et al. (2016)^*^	28 (F = 28)	fMRI	Whole brain	RSQ	S (covaried for Ds), Ds (covaried for S)	Valence and salience viewing of photographs of mother	Neutral viewing photographs of mother	**ME:** reported in Table 3. **CSAS, security for salience contrast, corrected for RSQ avoidance, depression, and anxiety:** deactivations in the R MTG, R STG, R cerebrum sub-Gyral, parietal lobe, R CG, **CSAS, avoidance for salience contrast, corrected for RSQ security, depression, and anxiety:** deactivations in the R cerebellum declive and L cerebellum (uvula, tuber and pyramis), R cuneus, R CG, R/L precentral gyrus, R PG, R CC, R IPL, L STG, L cerebrum sub-lobar extra-nuclear white matter	P <0,01 Voxel-wise probability threshold P <0,05 Cluster-wise probability threshold

Yaseen et al. (2016) investigated the neural correlates of attachment through both implicit and self-report measures, as mentioned in Table 3. Since the main findings do not distinguish between explicit or implicit results, their outcomes are not restated here. AMTG, anterior middle temporal gyrus; AMY, amygdala; Av, avoidant; Ax, anxious; BPD, borderline personality disorder; CG, cingulate gyrus; CC, corpus callosum; CSAS, correlates for separate attachment styles; DBAS, differences between attachment styles; DLPFC, dorsolateral prefrontal cortex; ECR, experiences in close relationships scale; FA, fearful-avoidant; FBA, fusiform body area; FFA, fusiform face area; FDR, false discovery rate; FFG, fusiform gyrus; fMRI, functional magnetic resonance imaging; HC, hippocampus; IFG, inferior frontal gyrus; INS, insula; IPL, inferior parietal lobule; IP, inferior parietal; MDD, major depressive disorder; ME, main effects; MFG, middle frontal gyrus; MOG, middle occipital gyrus; MOFC, middle orbitofrontal cortex; MPFC, middle prefrontal cortex; MTG, middle temporal gyrus; PCC, posterior cingulate cortex; PG, postcentral gyrus; PHG, parahippocampal gyrus; PREC, precuneus; PSTS, posterior superior temporal sulcus; ROI, region of interest; RQ, relationships questionnaire; RSQ, relationship structures questionnaire; S, secure; SMA, supplementary motor area; STG, superior temporal gyrus; TI, temporal inferior; VACC, ventral anterior cingulate cortex.

### Neuroscience of attachment

#### Implicit measures in healthy samples

We included four articles that implicitly measured neural correlates of attachment in mentally healthy individuals. Findings from three studies that controlled for mental health comorbidities are reported here as well. All studies were conducted using fMRI, apart from two records that utilized sMRI, which will be mentioned in the section below. Two records did not correct for multiple comparisons (Buchheim et al., 2006; Moutsiana et al., 2015) or did not specify (Lyons-Ruth et al., 2016); see Table 3.

Buchheim et al. (2006) were the first to assess the neural correlates of adult attachment styles. The authors conducted an ANOVA to compare two attachment groups in response to increasing activation of the attachment system by AAP stimuli. Unresolved participants, as opposed to resolved participants, showed enhanced activity in the right inferior frontal gyrus: an area involved in the reappraisal of social emotions and part of the ToM network (Grecucci et al., 2013; Iarrobino et al., 2021; Molenberghs et al., 2016). Another significant difference between the groups was a stronger engagement of the amygdala in unresolved participants. The amygdala subserves emotional behavior and fear conditioning and shows enhanced activity during more negative experiences (Šimić et al., 2021). In line with its functionality, the amygdala was incorporated into the “aversion module” of attachment (White et al., 2020; Šimić et al., 2021). None of the main effects of attachment corresponded with our hypothesized regions of interest, except for heightened stimulation of the left superior temporal gyrus in unresolved participants. However, its incidence was attributed to semantic retrieval rather than social cognition.

Lemche et al. (2006) measured skin conductance levels and neural activation of participants when observing prime sentences with pleasant, unpleasant, or neutral attachment-related content. It was hypothesized that greater insecurity would correlate with higher reaction times in the priming tasks, allowing neural activity to be assessed based on reaction times. Our focus lies with the results of negative prime sentences, such as “my mum rejects me,” since the attachment system is aroused by negative experiences (Simpson and Steven Rholes, 2017). Significant results were found in regions belonging to either the mentalizing module of attachment, corresponding to the left superior temporal gyrus, or the self-regulation module, associated with recruitment of the left dorsolateral prefrontal cortex (White et al., 2020). Of all results, only stimulation of the amygdala correlated with both skin conductance levels and the fMRI findings. However, enhanced activity in the putamen and insula—which the authors of the study predicted—was observed in the control condition as well and did not differentiate between attachment styles.

Petrowski et al. (2019) compared the faces of parents and romantic partners with unfamiliar faces and observed a main effect in the bilateral supramarginal gyrus, left precuneus, left posterior cingulate cortex, and right inferior frontal gyrus, corresponding to areas involved in social cognition and mentalizing (White et al., 2020; Molenberghs et al., 2016; Schurz et al., 2017). In contrast to previous studies, secure individuals showed greater activation in the left superior temporal gyrus when presented with the face stimuli, reflecting increased social processing (Petrowski et al., 2019). The authors attributed this disparity to the positive nature of the face stimuli used, whereas the previous two studies employed negative attachment experiences to elicit the attachment system. Finally, unlike the findings by Buchheim et al. (2006), the right inferior frontal gyrus was more strongly activated in insecure than in disorganized individuals. This was viewed as evidence of higher risk aversion in this group, an effect that persisted upon exposure to more positive face stimuli (Christopoulos et al., 2009). No suprathreshold voxels were found in other contrasts.

Yaseen et al. (2016) reported main effects from a salience task, “How much do you relate to this picture?” and a valence task, “How pleasant do you feel when you look at this picture?” while viewing pictures of the mother vs. neutrally viewing other pictures. Both tasks activated the posterior cingulate cortex, which is involved in mentalizing behavior (Brewer et al., 2013). Attachment security, covaried for AAI dismissiveness, was associated with activation in the right parahippocampal gyrus, right posterior cingulate cortex, and right fusiform gyrus—important areas for social cognition, empathy, and interpretation of non-verbal communication (Yaseen et al., 2016; Frith and Frith, 2012). Deactivations in the cuneus bilaterally were interpreted as decreased explicit attention to negative affect. Surprisingly, dismissing attachment did not correlate with the hypothesized activity in areas implicated in self-regulation. Conversely, the precuneus negatively interacted with mood in dismissive individuals in the right medial frontal gyrus and the precuneus, which was related to affect regulation (Etkin et al., 2011).

Three records controlled for mental health comorbidities. Therefore, the results are discussed in this paragraph, although the findings are reported in Table 4. Two studies by Buchheim et al. (2008) and Labek et al. (2016) employed the AAP with resolved and unresolved controls. The first study revealed significant activity in the left dorsolateral prefrontal cortex and left precuneus. The left precuneus is involved in understanding the minds of others, whereas enhanced dorsolateral prefrontal cortical activity was associated with cognitive control and emotion regulation (Molenberghs et al., 2016; Ochsner and Gross, 2005; Vrticka et al., 2013; White L. K. et al., 2023). The second study, conducted by Buchheim et al. (2008) and Labek et al. (2016), found the amygdala and right dorsolateral prefrontal cortex to be enhanced in unresolved controls. Significant recruitment of the dorsolateral prefrontal cortex was interpreted in terms of emotion regulation, with the right and left hemisphere activations indicating regulation of affective and verbal emotional content, respectively (White et al., 2020; Buchheim et al., 2008). Furthermore, increased recruitment was observed in the left superior temporal sulcus and medial prefrontal cortex in resolved controls, both regions pertaining to the neural correlates of social cognition (Frith and Frith, 2012). In addition, Galynker et al. (2012) found AAI insecurity, covaried for depression, to be associated with decreased engagement of the medial thalamus and ventral caudate when viewing pictures of mothers vs. strangers. These neural correlates are associated with reward, punishment, affectively motivated behaviors, and memory, demonstrating that insecure individuals are less inclined to engage in approach behaviors when presented with positive representations of attachment figures. In keeping with this interpretation, the posterior cingulate gyrus and the left precuneus were deactivated, reflecting decreased mentalizing. Moreover, increased insular engagement correlated with aversive experiences (Long et al., 2020; Huang et al., 2017).

Finally, two structural studies recruited adults whose attachment style was measured with the strange situation procedure (SSP) at 18 and 19 months, respectively. Moutsiana et al. (2015) reported greater right amygdala volumes for insecure participants, as opposed to securely attached individuals, with the bilateral amygdala and hippocampus as regions of interest, whereas Lyons-Ruth et al. (2016) observed a significant effect of attachment disturbance in the left amygdala, but not in the hippocampus, caudate, or thalamus. Although the right amygdala failed to reach significance in the latter study, the effect size suggested that activity in the right amygdala would have yielded significant results with a larger sample, similar to Moutsiana et al. (2015).

#### Implicit measures with comorbidity

In this review, nine studies were incorporated that implicitly assessed attachment styles in participants with a mental health condition, as specified in the inclusion criteria. All of the studies employed fMRI. Three articles did not correct for multiple comparisons (Buchheim et al., 2012b,a; Zhang et al., 2011) or only corrected the main findings (Buchheim et al., 2008); see Table 4.

Bernheim et al. (2022) presented participants, half of whom were diagnosed with borderline personality disorder (BPD), with personalized vs. neutral sentences prior to the AAP stimuli. A greater percentage of unresolved attachment classifications was found among BPD patients. Notably, the study observed significant neural differences between patients and controls only for monadic stimuli depicting situations of loneliness that elicited feelings of abandonment, whereas no results were found for dyadic pictures showing two persons, evoking experiences of potential social rejection. Upon comparing the two groups, BPD patients showed enhanced recruitment of the bilateral anterior insula, anterior midcingulate cortex, and right amygdala, which are implicated in the aversion module of attachment (Long et al., 2020; White et al., 2020). These regions are also involved in empathy responses to physical pain in others, with the anterior midcingulate cortex being engaged in processing both physical and emotional pain (Bruneau et al., 2012). Previous studies have reported extensive connections of these regions with ToM networks during experiences of social pain (Bernheim et al., 2022; Bruneau et al., 2012; Müller-Pinzler et al., 2015). A similar pattern was observed in this study, exemplified by activation of the posterior superior temporal sulcus and ventromedial prefrontal cortex.

Furthermore, two related articles by Buchheim et al. (2012a,b) utilized an AAP design with personally relevant vs. neutral sentences. Half of the participants had major depressive disorder (MDD) prior to 15 months of psychotherapeutic treatment. A significantly greater number of MDD patients were classified as unresolved, while controls were mostly resolved. Both studies reported enhanced activation of the anterior hippocampus, ventral anterior cingulate cortex, and left amygdala in the patient group, possibly reflecting implicit aversive responses during attachment relationships (Long et al., 2020; White et al., 2020). Furthermore, an interaction effect was observed between symptom severity and activity in the medial prefrontal cortex and anterior cingulate after psychotherapy. The authors associate activity in these regions with mood dysregulation and re-regulation (Buchheim et al., 2012b). This interpretation was supported by prior studies that revealed decreased glucose metabolism in the left anterior cingulate after 10 sessions of cognitive behavioral therapy (CBT) (Beutel et al., 2010; Sakai et al., 2005; Drevets, 2001). Interestingly, only 4 out of the 11 originally unresolved participants remained unresolved, indicating that the observed changes were associated with altered attachment styles.

Buchheim et al. (2013) investigated a single unresolved participant with narcissistic tendencies through a personalized AAP paradigm. The participant underwent functional magnetic resonance imaging (fMRI) immediately after a psychotherapy session. This procedure was repeated monthly over a 12-month period, while tracking the patient's mood and the quality of therapy. Increased activity in the dorsolateral prefrontal cortex was interpreted as a result of a greater ability to reflect on personal attachment-related issues, irrespective of therapy quality or the participants' mood. This observation corresponds to results by Buchheim et al. (2008), showing that unresolved controls also recruited the dorsolateral prefrontal cortex, while unresolved borderline patients, who were less able to self-regulate under attachment distress, did not. Furthermore, the posterior cingulate cortex interacted with therapy quality prior to the scan, revealing internal and interpersonal affect. Finally, the precuneus and medial prefrontal cortex were noted as important regions for mentalizing behaviors (Frith and Frith, 2012).

The outcomes of three studies that controlled for comorbidities were discussed in the previous paragraph. These studies also reported findings from a sample with mental health comorbidities. Buchheim et al. (2008) and Labek et al. (2016) explored the neural correlates of the adult attachment system in participants with and without BPD through the AAP. In the first study, separate analyses were performed for monadic and dyadic stimuli, as the inability to tolerate aloneness is an important marker of BPD (Buchheim et al., 2008; Gunderson, 1996; Gunderson and Lyons-Ruth, 2008). Unresolved BPD patients showed the greatest recruitment of the anterior cingulate cortex, possibly indicating pain and fear of abandonment (Pargament and Hahn, 1986; Spilka et al., 1985). In contrast to Bernheim et al. (2022), the authors reported greater activity in the right superior temporal gyrus in BPD patients when viewing dyadic AAP pictures. Activation of this region was attributed to a hyper-vigilant mentalizing strategy that is commonly observed after trauma (Kleshchova et al., 2019). Moreover, Labek et al. (2016) demonstrated that the dorsolateral prefrontal cortex was not significantly implicated in resolved controls and BPD patients. The absence of a response in BPD patients reveals dysfunctional emotion regulation. Conversely, unresolved controls were still able to self-regulate, as demonstrated by prefrontal dorsolateral activity (Nejati et al., 2022). This interpretation was supported by the observation that both unresolved controls and patients, but not resolved controls, recruited the amygdala in response to the attachment stimuli, reflecting both fear and distress (Šimić et al., 2021). Furthermore, enhanced activity in the anterior cingulate cortex was observed in unresolved BPD patients, consistent with an aversive response (Carter and van Veen, 2007). In addition, activation of the superior temporal sulcus reflected a hyperactive mentalizing strategy common in attachment distress (White L. et al., 2023; Long et al., 2020; White et al., 2020).

Kobayashi et al. (2020) exposed healthy controls and depressed participants to pictures of their mother or strangers. Without controlling for MDD, enhanced activation of mentalizing areas was found as a main effect. Furthermore, the right inferior frontal gyrus was implicated, a region consistently associated with ToM (Molenberghs et al., 2016). Notable deactivated areas included the right insular cortex, possibly implying decreased aversion to positive stimuli, as well as the supramarginal gyrus, which is involved in ToM (Schurz et al., 2017; Arioli et al., 2018). Deactivation of the supramarginal gyrus does not align with activity in the IFG, which is also a ToM region. This discrepancy might be explained by distinct cognitive and affective mechanisms underlying ToM processing (Molenberghs et al., 2016). Notably, insecure attachment and depression were found to converge in the cortico-striate-thalamic circuit, consistent with Bowlby's hypothesis that disrupted attachment bonds relate to an increased risk of depression (Galynker et al., 2012; Buchheim et al., 2013; Rajkumar, 2021).

Flechsig et al. (2023) assigned an AAP attachment score to BPD patients and healthy controls. The lowest score signified unresolved attachment, progressing to preoccupied and dismissing styles, with secure attachment receiving the highest score. The patient group exhibited the lowest score, which correlated with increased activation of the left amygdala, demonstrating an intensified emotional response related to social pain (Herpertz et al., 2001). Accordingly, the authors attributed the augmented recruitment of the anterior midcingulate cortex in the more unresolved group to experiences of social exclusion and attachment anxiety (Dewall et al., 2012). It was noted that enhanced recruitment of the anterior midcingulate cortex prior to therapy predicted diminished efficacy of therapy.

Zhang et al. (2011) conducted a principal component analysis with results obtained from depressed subjects and healthy controls while viewing a photograph of a mother, compared to viewing a friend or stranger. Their aim was to predict AAI coherence of mind and depression scores based on brain activity, to distinguish MDD from other mental disorders. The anterior midcingulate cortex was identified as a region of interest through general linear model (GLM) analysis, which demonstrated a correlation with both AAI and depression scores. Attachment security could not be robustly predicted from the two principal components, although the moderate relationship indicates that a larger sample might allow for more accurate estimations. Nonetheless, increased activity in the anterior midcingulate cortex was associated with depression, characterized by higher insecurity scores. Enhanced recruitment of this region during the viewing of pictures of a mother compared to a stranger was interpreted as an effect of conflict resolution and overcompensation for decreased regulatory activity in the subgenual cingulate (Etkin et al., 2011; Zhang et al., 2011).

#### Explicit measures in healthy samples

We included seven studies conducted with mentally healthy participants that utilized self-report attachment questionnaires. Four studies corrected for multiple comparisons (Vrtička et al., 2012; Yaseen et al., 2016; Liu et al., 2017; Rognoni et al., 2008), two did not (Canterberry and Gillath, 2013; Krause et al., 2016), and one study did not specifically mention (Nash et al., 2014) (see Table 5).

Canterberry and Gillath (2013) employed explicit and implicit attachment (in)security and neutral primes prior to the presentation of neutral drawings to instill a sense of security. For clarity and brevity, Table 5 only presents the results of explicit primes. Main effects of security priming exhibited activity in the medial prefrontal cortex associated with emotion regulation, as well as activation of the fusiform gyrus and parahippocampal gyrus related to reading the name of a loved one and retrieving secure internal working models, respectively. Individuals classified as avoidant showed increased activation in the parahippocampal gyrus, indicating intensified efforts to access secure models. They also recruited the amygdala and insula, regions associated with aversive emotional stimuli. Enhanced recruitment of mentalizing areas, such as the superior temporal gyrus, was ascribed to failed deactivation resulting from sequential exposure to security-related stimuli. Participants with high anxiety exhibited augmented engagement of the posterior cingulate and paracentral lobule, which was consistent with a hypervigilant attachment strategy and increased effort to recruit secure internal working models. Although implicit primes were not correlated with participants' ratings of (in)security or neutral stimuli, implicit security primes did reveal an association with anxiety in the right inferior frontal gyrus, which is implicated in ToM behaviors (Hartwright et al., 2016). For avoidant individuals, this contrast did not yield significant results, which the authors attributed to effective unconscious deactivation.

Krause et al. (2016) sought to understand whether secure, avoidant, or preoccupied narratives of others, as well as one's own attachment style, influence approach and aversion behaviors. Seed regions included the dorsal anterior cingulate cortex, hippocampus, and amygdala, which are part of the social aversion network. The most pronounced neural engagement was observed when listening to dismissing narratives. Avoidant participants exhibited heightened connectivity between the dorsal anterior cingulate cortex and the anterior middle temporal gyrus, as well as the medial prefrontal cortex after the dismissing narrative, reflecting increased sensitivity to dismissing content. Attachment anxiety, on the other hand, was related to greater connectivity between the left dorsal anterior cingulate cortex and the right dorsolateral prefrontal cortex, representing an increased need for explicit regulation of affective states.

Liu et al. (2017) investigated whether avoidant and secure participants would show differences in attention bias to emotional stimuli through a cue-target paradigm. Their aim was to identify the neural substrates of emotion deactivation in avoidant individuals. Findings revealed a positive correlation between avoidance and attentional engagement with activity in the right superior temporal gyrus, middle occipital gyrus, left medial frontal gyrus, cingulate gyrus, and the supplementary motor area, indicating early attentional engagement in avoidant individuals that subsequently allows them to deactivate affective input. Interestingly, the bilateral fusiform gyrus and middle occipital gyrus, associated with the detection of emotional information, exhibited increased activity during positive—but not negative—emotional stimuli for avoidant as opposed to secure individuals, possibly reflecting the unexpectedness of positive emotions. Avoidant participants experienced greater difficulty in withdrawing from negative than from positive attachment stimuli, while secure individuals showed the opposite tendency.

Vrtička et al. (2012) contrasted viewing pleasant and unpleasant social and non-social emotional scenes from the International Affective Pictures System (IAPS). Participants were instructed either to watch the pictures naturally, to use cognitive reappraisal, or to suppress their emotions. Most results were found during natural appraisal, implying that more conscious tasks might be performed equally well across attachment styles, whereas attachment styles predominantly affect habitual responses. As hypothesized, the task engaged many areas implicated in mentalizing behaviors, such as the medial prefrontal cortex, the precuneus, the posterior cingulate cortex, the posterior superior temporal sulcus bilaterally, and the fusiform face area (White et al., 2020). A stronger upregulation in the amygdala and the dorsolateral prefrontal cortex was detected in avoidant individuals during reappraisal, highlighting that avoidant individuals prefer to suppress rather than re-evaluate and think about socially unpleasant emotions. Furthermore, avoidant individuals recruited the dorsolateral prefrontal cortex and anterior cingulate cortex, which are involved in emotion regulation and tracking emotional conflicts during natural viewing. Alternatively, attachment anxiety correlated with activity in the right amygdala and left parahippocampal cortex for pleasant stimuli, the latter indicating improved access to emotional memories corresponding to a hyperactivation strategy.

Yaseen et al. (2016) investigated the neural substrates of attachment through both implicit and explicit measures. One of the main effects is mentioned in the first results section. Explicitly measured attachment security was negatively associated with right superior and medial temporal activations, indicating less effortful explicit self-regulation. In contrast, implicit security coincided with greater activity in the midline regions, attributed to an empathetic response to viewing pictures of mothers. Dismissiveness negatively interacted with the right cingulate cortex, among others, which may reflect decreased explicit mentalizing behavior consistent with a deactivation strategy (White L. et al., 2023; Long et al., 2020). It is noteworthy that Yaseen et al. (2016) used natural images of mothers as a control condition, whereas Vrtička et al. (2012) found that attachment styles predominantly influenced responses to naturally viewed emotional stimuli. Thus, results should be interpreted with caution.

Two studies employed EEG. First, Nash et al. (2014) detected an error-related negativity (ERN) signal in participants after exposure to threatening insecurity-related thoughts. Prior to the threat, no differences were observed between individuals with insecure and secure attachment styles. However, insecure individuals exhibited an augmented ERN amplitude after the threat stimulus at the frontal and central midline. Their findings suggest that secure individuals recover more easily from insecurity threats. Conversely, the results indicate hypervigilance to insecurity threats in insecure individuals. The second EEG study was conducted by Rognoni et al. (2008), who hypothesized, based on prior research, that activity in the left hemisphere denotes approach behavior, whereas right frontal activity indicates withdrawal. Attachment avoidance correlated with greater right frontal asymmetry when viewing happy emotional scenes, highlighting that avoidant individuals overlook opportunities to satisfy attachment needs. Emotionally negative scenes were associated with higher left asymmetry, interpreted as inhibition of attachment behaviors and decreased salience of aversive experiences. Notably, the authors identified an inverse pattern for anxious individuals, who exhibited left hemispheric activity during happy emotional scenes due to an increased longing for closeness and proximity. In the case of negative stimuli, insecure participants displayed enhanced right hemispheric activity, attributed to sensitivity to rejection and separation. Likewise, fearful-avoidant individuals revealed a left-sided pattern aligned with a strong desire for interpersonal intimacy. It is noteworthy that they demonstrated a mismatch between self-reported arousal and frontal asymmetry: fear was consciously evaluated as less stimulating while simultaneously displaying an enhanced right-hemispheric fear response.

#### Structural neurobiological findings of attachment (VBM)

Seven studies were included that mapped the structural neurobiological findings through voxel-based morphometry (VBM), a method that compares gray matter volumes (GMV) across different brain regions. Two structural studies used implicit measures to assess attachment styles and were therefore discussed under “*implicit measures in healthy samples”*. Results of the remaining five studies were corrected for multiple comparisons (Acosta et al., 2018; Benetti et al., 2010; Jin et al., 2016; Redlich et al., 2015; Zhang et al., 2018a). Furthermore, only one study recruited participants with a mental health comorbidity (Jin et al., 2016). For specifications, see Table 6.

**Table 6:** Structural neurobiological findings of attachment (VBM).

**References**	**Sample size (female)**	**Imaging technique**	**Neuroimaging modality**	**Field of view**	**Attachment instrument**	**Attachment styles assessed**	**Main findings: volumetric results across attachment styles (R, right; L, left)**	**Corrected for multiple comparisons**
Acosta et al. (2018)	192 (F = 96)	sMRI	VBM	Whole brain	RSQ	**S**, Secure; **Av, Avoidant; Ax**, anxious; FA, fearful-avoidant newline Regression analysis with Av, Ax, FA, and GMV	**ME**, main effects of attachment stimuli including all classification; **CSAS**, correlates for separate attachment styles; **DBAS**, differences between attachment styles newline **CSAS, Av:** L STG, L cerebellum inferior semilunar lobule, decreased volumes in the R/L HC, L AINS **DBAS: Av** **>** **Ax:** L cerebellum, decreased volumes in the L AINS and the pars opercularis of the L IFG **CSAS, Ax:** left lateral orbital gyrus, decreased volumes in the R anterior TP, left HC	*P* < 0,05 voxel-wise FWE corrected
Benetti et al. (2010)	32 (F = 17)	sMRI	VBM	Whole brain	ECR-R	Regression analysis with Av, Ax, and GMV	**CSAS, Av:** increased GMV without correcting for multiple comparisons: L STG **CSAS, Av:** no suprathreshold voxels after correcting for multiple comparisons. **CSAS, Ax:** increased GMV in L LOG, decreased volumes in the R MTG, R IT	P <0,05 - FDR corrected
Jin et al. (2016)	68 (F = 36) HC 34 BPD 34	sMRI	VBM-DARTEL	Whole brain	ASQ	Gray matter volume and concentration differences were regressed on insecurity scores for BPD patients and healthy controls	**CSAS, insecurity in healthy controls:** decreased GMV in PREC, MCC, MOG **CSAS, insecurity in BPD patients:** no GMV differences were observed **DBAS, more insecurely attached BPD patients** **>** **healthy controls:** PREC, MCC, PCC	*P* < 0,1 Voxel-wise FDR corrected *P* < 0,05 Cluster size 50+ voxels, cluster-wise probability threshold
Lyons-Ruth et al. (2016)^*^	18 (F = 10)	sMRI	VBM, GMV analysis using FSL	ROI: AMY, HC, caudate and thalamus	SSP	Disorganized (12 participants or 67% of sample assessed in infancy) and 6 resolved.	**CSAS, attachment disturbance (disorganization and disruption in mother–infant interaction) at 18 months of age:** L AMY, but not in HC, caudate, and thalamus	Not mentioned
Moutsiana et al. (2015)^**^	59 (F = 29)	sMRI (T1)	VBM	ROI: hippocampus and amygdala	SSP	Secure vs. insecure and vice versa regressed on volumetric differences in the ROI's	**DBAS, insecurity** **>** **security:** greater R but not L AMY volume **DBAS, insecurity** **>** **security:** no significant differences in HC volume observed between secure and insecure individuals	Uncorrected
Redlich et al. (2015)	306 (F = 154)	sMRI	VBM	whole brain with ROI: bilateral amygdala	RSQ	Regression analysis with Ax and GMV	**CSAS, Ax, increased GMV in:** R/L AMY	*P* < 0,05 Voxel-wise FWE corrected
Zhang et al. (2018a)	106 (F = 57)	sMRI	VBM	Whole brain	ECR (Chinese version)	Regression analysis with Av, Ax, and GMV	**CSAS, Av, decreased GMV in:** L MTG, L STG, L TP, R PHG after controlling for attachment anxiety, sex, age, and global GMV **CSAS, Ax, decreased GMV in:** R ACC, MFG, OFC after controlling for attachment avoidance, sex, age, and global GMV	*P* < 0,001 Voxel-wise Monte Carlo simulations (1,000) to derive a *P* < 0,05 Cluster-wise threshold combining a *P* < 0,001 height threshold and a minimum cluster size of 527 contiguous voxels

AINS, anterior insula; ASQ, attachment style questionnaire; Av, avoidance; Ax, anxiety; BPD, borderline personality disorder; CSAS, correlates for separate attachment styles; DBAS, differences between attachment styles; DTI, diffusion tensor imaging; ECR, experiences in close relationships scale; ECR-R, experiences in close relationships revised; FA, fearful-avoidant; FWE, family-wise error GMV, gray matter volume; HC, healthy controls; hippocampus; IFG, inferior frontal gyrus; LOG, lateral orbital gyrus; MCC, middle cingulate cortex; ME, main effects; MFG, middle frontal gyrus; MOG, middle occipital gyrus; MTG, middle temporal gyrus; OFC, orbitofrontal cortex; PCC, posterior cingulate cortex; PHG, parahippocampal gyrus; PREC, precuneus; ROI, region of interest; RSQ, relationship scales questionnaire; sMRI, structural magnetic resonance imaging; SSP, strange situation procedure; STG, superior temporal gyrus; TP, temporal pole; S, secure; VBM, voxel-based morphometry.

^*^ and ^**^ mark the two studies that employed implicit measures for attachment classification. Other studies used self-report measures.

Fonagy and Luyten (2009) found an inverse correlation between attachment avoidance and gray matter volume in the insular lobe, which is linked with altered emotion processing and reduced subjective feelings compared to anxious individuals. Furthermore, the inferior frontal gyrus exhibited decreased activity in avoidant individuals (Fonseka et al., 2016). Conversely, anxious individuals showed heightened recruitment in both areas, which may reflect an intensification of negative emotions.

Benetti et al. (2010) assessed the relationship between attachment styles, brain structures, and affective loss. After controlling for multiple comparisons, the authors did not identify significant volumetric correlations with avoidance. However, attachment anxiety was positively related to gray matter volume in the left lateral orbital, which may reflect increased emotion regulation in both positive and negative conditions. Decreased volumes in the right temporal pole were linked to emotion processing. The authors noted that reduced metabolic activity was also found in this region during major depression.

Jin et al. (2023) sought to identify neural correlates of insecure attachment in healthy controls and borderline patients. Increased GMV in the posterior cingulate cortex and the precuneus in BPD patients was attributed to stronger emotional engagement, increased depersonalization, and disturbed self-referential processing. However, attachment insecurity did not correlate with any of the regions in BPD patients, whereas the posterior cingulate cortex and precuneus demonstrated lower gray matter volumes in insecure healthy controls. The authors therefore proposed that BPD may obscure the effects of insecurity on gray matter volume in the aforementioned regions.

Redlich et al. (2015) performed a whole brain analysis to identify potential structural changes based on attachment styles. A positive association between separation anxiety and bilateral amygdala gray matter volume was found, with the strongest effect observed in the right hemisphere. Findings were adjusted for general measures of depression level, anxiety, and sociodemographic factors. These outcomes indicate a hyper-reactive attachment response, which is commonly observed in preoccupied attachment.

Finally, Zhang et al. (2018b) identified a negative correlation between avoidance and gray matter volumes in the parahippocampal gyrus, as well as in the left middle and superior temporal gyrus. The results may reflect difficulties in the retrieval of emotional memories, as is also observed in maltreated children. Moreover, reduced left superior temporal structural volumes suggest impaired empathy in interpersonal interactions, consistent with decreased mentalizing behaviors in attachment avoidance. Anxiety negatively correlated with anterior cingulate gray matter volume, indicating a reduced ability to inhibit negative emotions.

#### Structural neurobiological findings of attachment (DTI)

Although one of the four articles using Diffusion Tensor Imaging (DTI) reported both DTI and VBM results, this study is discussed in the current section (Picerni et al., 2022). In contrast to VBM, DTI is commonly used to obtain information about the integrity of white matter structures in the brain (O'Donnell and Westin, 2011). In addition to DTI, one of the included studies assessed patients with a mental health comorbidity through Diffusion Weighted Imaging (DWI) (Bracht et al., 2022). All studies, except for one by Serra et al. (2015), corrected their results for multiple comparisons (Table 7; Picerni et al., 2022; Bracht et al., 2022; Quirin et al., 2010; Rigon et al., 2016).

**Table 7:** Structural neurobiological findings of attachment (DTI).

**References**	**Sample size (female)**	**Imaging technique**	**Neuroimaging modality**	**Field of view**	**Attachment instrument**	**Attachment styles assessed**	**Main findings: volumetric results across attachment styles (R, right; L, left)**	**Corrected for multiple comparisons**
Bracht et al. (2022)	66 (F/M ratio not significantly different across groups) HC 18 MDD 48	dMRI	Diffusion weighted MRI	ROI: CMPH	AAS-R	**S**, secure **Av**, avoidant; **Ax**, anxious; **FA**. fearful-avoidant newline Gray matter volume regressed for attachment anxiety and avoidance newline Low Ax: 28 High Ax: 20 Low Av: 21 High Av: 27	**ME**, main effects of attachment stimuli including all classifications; **CSAS**, correlates for separate attachment styles; **DBAS**, differences between attachment styles newline **CSAS, Av, Mean Diffusivity (MD):** decreased volumes in the HC and higher mean diffusivity in the PHC **CSAS, Ax, mean diffusivity (MD):** no volumetric or mean differences in HC and PHC	P <0,025 Bonferroni corrected
Picerni et al. (2022)	79 (F = 43)	sMRI	DTI and VBM on the cerebellum	ROI: R/L OFC, middle frontal area, INS, CC, and cerebellum	AAS	Ax	**CSAS, Ax, increased GMV in:** L cerebellum crus 2, R cerebellum lobule VI, R MOFC	P <0,05 Cluster size 50 voxels, Cluster-wise FWE corrected
Quirin et al. (2010)	22 (F = 11)	sMRI	DTI	ROI: R/L HC	ECR	Regression analysis with Av, Ax, and gray matter concentration	**CSAS, Av, decreased GMV in:** L HC, revealing a trend toward decreased GMV in R HC as well **CSAS, Ax, decreased GMV in:** L HC	Threshold of t = 3.53 (P <0.001, uncorrected) to identify significant cluster activations P-values at cluster level were determined using RFT
Rigon et al. (2016)	20 (F = 20)	sMRI	DTI	ROI: AMY, HC, Caudate	ECR-R	Av and Ax	**CSAS, Av, mean diffusivity (MD) controlled for anxiety:** lower structural integrity in R/L AMY but no correlation in HC and caudate **CSAS, Av, fractional anisotropy (FA) controlled for anxiety:** greater more organized and compact white matter fibers in the R UF **CSAS, Ax controlled for avoidance:** no significant correlations in the ROIs	P <0,008 Bonferroni corrected
Serra et al. (2015)	53 (F = 22)	sMRI	DTI	ROI: UF, CM, FM, IFOF, ILF, SLF	SS	S	**CSAS, security, mean diffusivity (MD):** no significant correlations in the ROIs **CSAS, security, fractional anisotropy (FA):** greater FA in L UF, L IFOF, L SLF, and L CMh **CSAS, security, axial diffusivity (AD):** No significant correlations in the ROIs **CSAS, security, radial diffusivity (RD):** lower RD in L UF, L IFOF, L SLF, L CMh	Not mentioned

AAS: adult attachment style AAS-R: revised adult attachment scale ACG: anterior cingulate gyrus AD: axial diffusivity Av: avoidance; Ax: anxiety; CC: cingulate cortex CMh: hippocampal region of the cingulum; CMPH: parahippocampal region of the cingulum; DWI: diffusion weighted imaging; FA: fearful-avoidant/fractional anisotropy; FM: forceps minor; IFOF: inferior frontal occipital fasciculus; ITG: inferior temporal gyrus; MDD: major depressive disorder; MD: mean diffusivity; RD: radial diffusivity; S: secure; SLF: superior longitudinal fasciculus; SS: Kerns security scale UF: uncinate fasciculus.

Bracht et al. (2022) explored the association between insecure attachment, depression, and structural changes in the hippocampal cortex and parahippocampal cingulum. Individuals with high attachment avoidance exhibited smaller hippocampal volumes, as well as higher mean diffusivity in the parahippocampal cingulum compared to those with low avoidance and healthy controls. The results reflect potential effects of insecure attachment on brain functioning that overlap with the effects of depression. No differences were observed for attachment anxiety or between patients with depression and healthy controls.

Picerni et al. (2022) did not detect a significant effect of attachment avoidance; however, an increase in cortical volume for the superior temporal gyrus was observed after lowering the statistical threshold, in accordance with Benetti et al. (2010). Anxiety correlated with enhanced cortical volume in the right middle orbitofrontal cortex and cerebellar areas, but not with other regions of interest that aligned with our hypothesis, such as the cingulate cortex and the insula. The authors suggest that the neural correlates of attachment should be expanded by including specific subdivisions of the cerebellum, consistent with prior studies that assign a role in mentalizing behaviors and self-related emotions to those areas (Van Overwalle et al., 2020).

Quirin et al. (2010) discovered that attachment avoidance and anxiety correlated with decreased left hippocampal volume. In addition, avoidance showed a trend toward significance in the right hippocampus. Their findings align with previous evidence indicating that attachment insecurity is related to other factors leading to reduced hippocampal volume, such as chronic stress, lower quality of parental care, and dysregulated glucocorticoid regulation (Quirin et al., 2010).

Rigon et al. (2016) contrasted attachment anxiety and avoidance by regressing the attachment styles against mean diffusivity in the amygdala. Lower structural integrity in the left, and to a lesser degree, right amygdala was found for attachment avoidance, whereas no differences were observed for the bilateral hippocampus and bilateral caudate. All findings remained significant after adjusting for the Big Five personality traits. The results indicated increased regulation of output from the amygdala. Increased organization of white matter fibers from the amygdala to prefrontal areas was also found. These observations were interpreted as reflecting a deactivating emotion regulation strategy. In contrast to previous studies, no findings reached significance for anxiety.

Serra et al. (2015) used diffusion tensor imaging to evaluate the integrity of white matter tracts. Consistent with their hypothesis, secure attachment was associated with greater integrity of four white matter tracts in the left hemisphere, linked to social competence, better affect regulation, and higher quality of maternal relationships. The apparent prevalence of left, but not right hemispheric tracts may support the importance of the left hemisphere in internal working models of security and attachment in general.

### Neural correlates of Christian prayer

The neural correlates of Christian prayer were reported in 13 studies. Only those studies that revealed activation during a prayer task compared to a control condition were included, while indirect assessments of the influence of religion and spirituality on brain functioning were excluded. Moreover, we excluded one study during the quality assessment. Six articles failed to correct for multiple comparisons (Silveira et al., 2015; Beauregard and Paquette, 2006, 2008; Azari et al., 2001, 2005; Surwillo and Hobson, 1978). See Tables 8, 9 for more details.

**Table 8:** Neural correlates of Christian prayer.

**References**	**Sample size (female)**	**Imaging technique**	**Field of view**	**Prayer task**	**Control task**	**Main findings**	**Corrected for multiple comparisons**
Azari et al. (2001)	6 (F = 2)	PET	Whole brain	Reading religious text (psalm 23) and reciting religious text with eyes closed	Reading nursery rhyme, reading instructions silently and resting state	**Religious-recite** **>** **resting state:** R/L DLPFC, dorsomedial frontal cortex, R medial parietal (PREC), L cerebellum	Uncorrected
Azari et al. (2005)	6 (F = 2)	PET	Whole brain	Reading religious text (psalm 23) and reciting religious text with eyes closed	Reading nursery rhyme, reading instructions silently, and resting state	**PC9 religious** **>** **happy state, positive correlations:** L IPL, L MTG, L SFG, R IFG, R lower premotor, R OFG, L lateral cerebellum, **PC9 religious** **>** **happy state, negative correlations:** R/L inferior/lateral frontal, L SFG, L PREC, L lingual gyrus **PC11 religious** **>** **rest, positive correlations:** R OFG, R SFG, R parietal operculum, L neocerebellum, R/L posterior cerebellum **PC11 religious** **>** **rest, negative correlations:** R SFG R ACC	Uncorrected
Elmholdt et al. (2017)	28 (F = 16)	fMRI	Whole brain	15 pain stimulus trials with prayer to God	15 pain stimulus trials with secular prayer to Mr. Hansen	**Prayer** **>** **secular prayer:** no positive correlations. **Secular prayer** **>** **prayer:** increased activation of R Frontal Eye Field, DLPFC, LOFC, PREC, R/L PCC	P <0,05 Voxel-wise FWE corrected
Beauregard and Paquette (2006)	15 (F = 15)	fMRI	Whole brain	Reliving the most intense mystical experience	Reliving the most intense state of union with another human ever felt after joining the monastery, as well as baseline activity	**Reliving mystical experience** **>** **control:** R MOFC, R MPFC, L IPL, R MTC, L SPL, R ACC **Reliving mystical experience** **>** **baseline:** R MOFC, R MTC, R/L IPL, R SPL, R/L caudate, R/L MOC, L MPFC, R LG, L brainstem, L INS, L ACC **Control** **>** **baseline:** R/L SPL, L caudate, R IOC, L ACC, L brainstem **Control** **>** **reliving mystical experience:** L putamen	Uncorrected
Beauregard and Paquette (2008)	15 (F = 15)	EEG	Scalp locations: FP1/FP2, F7/F8, F3/F4, FZ, T3/T4, C3/C4, CZ, P3/P4, T5/T6, PZ, O1/O2	Reliving the most intense mystical experience	Reliving the most intense state of union with another human ever felt after joining the monastery, as well as baseline activity	**Reliving mystical experience** **>** **control:** increased Theta power over left and central frontal and parietal regions **Reliving mystical experience** **>** **control:** increased gamma1 power in the right temporal and parietal regions **Reliving mystical experience** **>** **control:** increased theta connectivity between left frontal and central areas **Reliving mystical experience** **>** **control:** increased long-distant alpha connectivity between R frontal and R temporal and R parietal regions, and between R central and R parietal regions	Uncorrected
Galanter et al. (2017)	18 (F = 8)	fMRI	Whole brain	Prayer during viewing alcohol-craving-inducing images after reading prayers	Passive reading of instructions before watching alcohol-craving-inducing images and passive viewing	**Prayer** **>** **average of control conditions:** L AMFG, L SPL, R/L precuneus, R/L PMTG **Prayer** **>** **average of control conditions, additional areas of activation identified through PCA:** R lateralized INS, IPC, TPJ, L caudate nucleus	P <0,05 Cluster-wise FWE corrected
Kober et al. (2017)	40 (F = 22)	EEG	Scalp Locations: Cz	Neurofeedback performance by people who frequently prayed	Neurofeedback performance by individuals who did not frequently pray	**Participants reporting high prayer frequency:** linearly increased their sensorimotor (SMR)-to-theta ratio, indicating better neurofeedback performance **Participants reporting low prayer frequency:** did not linearly increase their SMR-to-theta ratio, as suggested by the slightly decreasing regression slopes, indicating degraded neurofeedback performance	P <0,05 Cluster-wise FWE corrected
Leighton (2021)	45 (F = 28)	EEG	ROI: IFG, SFG, OFG, ACC	Prayer of adoration and prayer of forgiveness	Baseline activity	**Prayer** **>** **baseline:** no significant differences between the three conditions (prayer of adoration, prayer of forgiveness, baseline control) were observed for PFC ROIs **Prayer** **>** **baseline:** no significant differences between the three conditions (prayer of adoration, prayer of forgiveness, baseline control) were observed for ACC ROI **Prayer** **>** **baseline:** increased alpha CSD in the STG and PCG	*P* < 0,05 Bonferroni for *post-hoc* pairwise comparison
Neubauer (2014)	14 (F = 8)	fMRI	Whole brain	Active prayer	Imagining to silently speak to a loved one, and imagining and naming animals	**Prayer** **>** **baseline:** increased activation in the R/L MPFC, L PCG, L PREC, left parietal lobe **Prayer** **>** **imaginatively speaking to a loved one:** increased activation of juxtapositional lobule and L insular cortex **Imaginatively speaking to a loved one** **>** **Prayer:** R Frontal Pole, R/L Precuneus	*P* < 0,05 Cluster-wise corrected after setting a voxel-wise threshold (z = 2.3)
Schjødt et al. (2008)	20 (F = 14)	fMRI	ROI: bilateral Caudate Nucleus	Participants prayed the Lord's prayer and a personal, improvised prayer	A well-known nursery rhyme and wishful praying to Santa Claus respectively	**The Lord's prayer** **>** **control:** R caudate **Personal improvised prayer** **>** **control:** R caudate	*P* < 0,003 FWE corrected
Schjoedt et al. (2009)	20 (F = 14)	fMRI	Whole brain	Participants prayed the Lord's prayer and a personal, improvised prayer	A well-known nursery rhyme and wishful praying to Santa Claus	**Personal prayer** **>** **making wishes to Santa Claus:** Increased activation of the L PREC, L TPJ, L temporopolar region, L MPFC **Making wishes to Santa Claus** **>** **personal prayer:** R/L DLPFC, R SMA, R posterior limbic cortex, R/L PREC, R OC, L motor cortex **Personal prayer** **>** **the Lord's prayer:** L DMPFC, L AMPFC, L temporopolar region, L TPJ, L PREC **The Lord's prayer** **>** **personal prayer:** R/L DLPFC, R parietal cortex, R/L cerebellum, R ITC, R PCC, L superior parietal cortex **The Lord's prayer** **>** **nursery rhyme:** No suprathreshold voxels **Nursery rhyme** **>** **the Lord's prayer:** No suprathreshold voxels	*P* < 0,05 Voxel-wise FDR corrected with extended threshold of 15 voxels
Silveira et al. (2015)	1 (F = 0)	fMRI	Whole brain	Praying the Lord's prayer	Resting state (baseline activity)	**The Lord's prayer and baseline activity:** both resting state and prayer manifested activations in the PREC, ACC, PCC, VMPFC, parieto-occipital junction	Uncorrected
Surwillo and Hobson (1978)	6 (F = 3)	EEG	Scalp locations: P3-O1 P4-O2 and P3-P4, O1-O2	Praying silently as the person is familiar with at home or in the church. Participants were asked to concentrate on prayers of adoration and praise	Resting state (baseline activity)	**Prayer** **>** **resting state:** a wavelength frequency shift from 9,6 Hz to 11, 2 Hz during prayer	Uncorrected

ACC, anterior cingulate cortex; AMFG, anterior middle frontal gyrus; AMPFC, anterior medial prefrontal cortex; CSD, current source density; DMN, default mode network; DLPFC, dorsolateral prefrontal cortex; DMPFC, dorsomedial prefrontal cortex; EEG, electroencephalogram; fMRI, functional magnetic resonance imaging; FWE, family-wise error; IFG, inferior frontal gyrus; INS, insula; IPL, inferior parietal lobule; IOC, inferior occipital cortex; LG, lateral geniculate; LOFC, lateral orbitofrontal cortex; MFC, medial frontal cortex; MOFC, medial orbitofrontal cortex; MOC, middle occipital cortex; MPFC, medial prefrontal cortex; MTG, middle temporal gyrus; MTC, middle temporal cortex; PC(A), principal component (analysis); PCC, posterior cingulate cortex; PCG, postcentral gyrus; PET, positron emission tomography; PFC, prefrontal cortex; PMTG, posterior medial temporal gyrus; PPC, posterior parietal cortex; PREC, precuneus; ROI, region of interest; SFG, superior frontal gyrus; SPECT, single photon emission computed tomography; SPL, superior parietal lobule; STG, superior temporal gyrus; TPJ, temporo-parietal junction; VMPFC, ventromedial prefrontal cortex.

**Table 9:** Structural findings of prayer.

**References**	**Sample size (female)**	**Imaging technique**	**Neuroimaging modality**	**Field of view**	**Distinguishment between groups**	**Main findings**	**Corrected for multiple comparisons**
Kober et al. (2017)	40 (F=22)	sMRI	VBM	ROI: R IFG, R INS, L PCG, L MOFC	Low or high prayer frequency on neurofeedback performance	**NF performance for participants reporting high prayer frequency:** decreased GMV in the L MOFC **NF performance for participants reporting low prayer frequency:** increased Orbital part of R IFG including R INS, decreased L PCG, L IPL	*P* < 0,05 Cluster-wise FWE corrected

FWE, family-wise error; GMV, gray matter volume; IFG, inferior frontal gyrus; INS, insula; IPL, inferior parietal lobe; MOFC, middle orbitofrontal cortex; NF, neurofeedback; PCG, postcentral gyrus; ROI, region of interest.

Two of the included studies conducted by Azari et al. (2001, 2005) compared the recitation of Psalm 23 with a resting state for six Christian participants. Neural engagement was observed within the bilateral dorsolateral prefrontal cortex, which the authors attributed to the activation of religious schemas, reflecting a readiness for religious experiences. Activities within the precuneus were interpreted as an effect of visual memory, possibly resulting from the visualization of imagery in the recited psalm. Notably, our own hypothesis indicates a role for this region in mental state representation (White et al., 2020). In contrast to Schjoedt et al. (2009), discussed below, the results held for all regions except the left dorsolateral prefrontal cortex when compared to the recital of a happy nursery rhyme. In a subsequent study, Azari et al. (2005) subjected the retrieved results to a principal component analysis (PCA) to distinguish neural representations of religious experiences from non-religious emotional states. They identified the negative loading patterns of PC9 and PC11 as being associated with religious experiences, the first of which negatively corresponded with the precuneus in a slightly more caudal position. PC11 is negatively associated with frontal regions, possibly related to self-referential mental activity. The fact that the limbic system was not implicated in the results may indicate that religious experiences are complex cognitive phenomena that are not predominantly grounded in automatic brain responses.

Elmholdt et al. (2017) found that prayer was correlated with reduced pain sensation through an expectation (non-opioid-linked) neural system that could aid participants in dissociating from negative aspects of pain. No significant positive associations with prayer compared to secular prayer were observed. The reverse contrast exhibited significant areas of activation in several regions, including the precuneus and the dorsolateral prefrontal cortex. This was interpreted as an indication of enhanced working memory, executive control, cognitive appraisal, and attentional modulation.

Beauregard and Paquette (2006) conducted two studies employing fMRI and EEG. When asking Carmelite nuns to relive their most intense experience of union with God, which was contrasted with experiencing the most intense union with another human being after joining the order, the authors were surprised to find neural differences during the two socio-emotional tasks. Contrary to our expectations that activity in the insula and anterior cingulate cortex would align with the aversion module of attachment, insular recruitment was attributed to the representation of somatovisceral reactions reflecting joy and unconditional love, whereas the anterior cingulate was implicated in the interoceptive detection of emotional signals (Lotze, 2024). In addition, recruitment of the left medial prefrontal cortex possibly served conscious awareness of these emotions. The possibility that the emotional valence of the prayer task might be more negative than accounted for is challenged by increased caudal activations related to happiness, romantic love, and maternal love. In the subsequent EEG study, Beauregard and Paquette (2008) found increased theta power in frontal regions during the mystical condition. These findings may demonstrate feelings of joy and unconditional love but are equally likely to represent sustained attention. Moreover, enhanced gamma activity in the right temporal region could signify the impression of union with God.

Galanter et al. (2017) recruited currently abstaining members of Alcoholics Anonymous who experienced a spiritual awakening. The participants viewed alcohol-craving-inducing images during prayer after reading AA prayers related to promoting abstinence. These outcomes were contrasted with passive viewing or reading unrelated information prior to the task. Increased activity in prefrontal areas indicated voluntary control of emotions, whereas posterior temporal areas manifested semantic reappraisal of emotions. Notably, a subsequent principal component analysis revealed the right insula, inferior prefrontal cortex, and temporoparietal junction to be involved during prayer, indicative of bottom–up control, empathy, and ToM, in line with our hypothesis.

Based on previous findings obtained through a brain–computer interface (BCI) paradigm, Kober et al. (2017) expected individuals who regularly pray to show improved performance during a neurofeedback task due to enhanced cognitive control. Indeed, participants with high prayer frequency were able to linearly increase their sensorimotor-rhythm waves over the course of nine trials, whereas neurofeedback performance levels decreased in participants with low prayer frequency, even though only a small percentage of participants prayed during the task. Better individual performance correlated with increased gray matter volume in the left medial orbitofrontal cortex, a region that the authors linked to performing unspecified tasks. Furthermore, structural results demonstrated that neurofeedback performance positively correlated with the right insula, which may coincide with focusing on the present moment.

Leighton (2021) did not find any significant differences in brain activity in prefrontal areas or the anterior cingulate cortex when comparing prayers of adoration and forgiveness to baseline activity. To provide a rationale, the author states that using EEG rather than other neuroimaging techniques may have skewed their results since the findings were incongruent with other prayer studies. Beyond the postulated regions of interest, the author observed an association between prayer and temporal lobe activity, suggestive of the recall of negative memories. Additionally, the postcentral gyrus was markedly active, possibly related to different components of language processing. Interestingly, the involvement of the temporal region aligns with our hypothesis, given its role in ToM and mentalizing behaviors, despite not being explicitly addressed in the discussion (Beauchamp, 2015).

Neubauer (2014) aimed to elucidate the nature of prayer by comparing personal prayer to imaginatively speaking to a loved one and a baseline control. The outcomes demonstrated enhanced activations in core areas of theory of mind and mentalizing behavior, such as the posterior cingulate cortex, the parietal lobe near the temporoparietal junction, and the medial prefrontal cortex. Those areas were also implicated in the default mode network, subserving introspective thoughts related to the self. Notably, the authors argue that the pattern of observed activation indicates that, for participants, God was experienced as just as real as their loved ones, since perceived interactions with fictional characters may depend on different neural substrates (Abraham et al., 2008).

Two studies conducted by Schjoedt et al. (2009) and Schjødt et al. (2008) contrasted personal improvised prayer and formalized prayer (the Lord's prayer) with making a wish list to Santa Claus or internal speech of a well-known rhyme. A main effect of right caudal activity during prayer as opposed to control conditions was observed after a region of interest analysis focusing on the bilateral caudate. These activations were interpreted as a function of both rewarding repetitive behaviors and the recruitment of the dopaminergic system as a manifestation of trust in God. The second study by Schjoedt et al. (2009) observed, through a whole brain analysis, activation in the anterior medial prefrontal cortex, the precuneus, the temporopolar region, and the temporoparietal junction, in accordance with observations by Neubauer (2014). The authors similarly attributed the stimulated areas to theory of mind as part of social cognition, supported by the default mode network, which may demonstrate the perceived reality of God and expected reciprocity with God. A possible explanation could be habituation, as reciting the Lord's prayer showed no significant differences from the nursery rhyme and demonstrated no meaningful associations with brain regions linked to social cognition.

Silveira et al. (2015) measured the Lord's prayer as well as resting-state connectivity in a Catholic bishop. After decomposing the results into independent components, eight connectivity networks were identified, including the default mode network. However, no significant differences in functional connectivity were observed, suggesting that the neuroscience of religious prayer resembles resting. Interestingly, the previous study could not identify the default mode network in formalized prayer, indicating that the formalized prayer employed by the bishop might involve experiencing social interaction with God beyond habituation effects.

Finally, Surwillo and Hobson (1978) were interested in potential overlap between transcendental meditation and prayer, as the former coincided with a slowing of electrocortical rhythms. Alternatively, they observed that Christian prayers of adoration and praise, compared to resting, correlated with increased bilateral EEG frequency signals between the parietal and occipital lobes, potentially reflecting heightened mental activity. The authors noted that the speeding of the signal might correspond with effects observed in professional yoga meditators.

## Discussion

This systematic review aimed to investigate whether the neural correlates of attachment relationships and prayer converge. The included studies were mostly of good quality, although one record was excluded during the quality assessment.

### Mentalizing module

**Figure 2:**
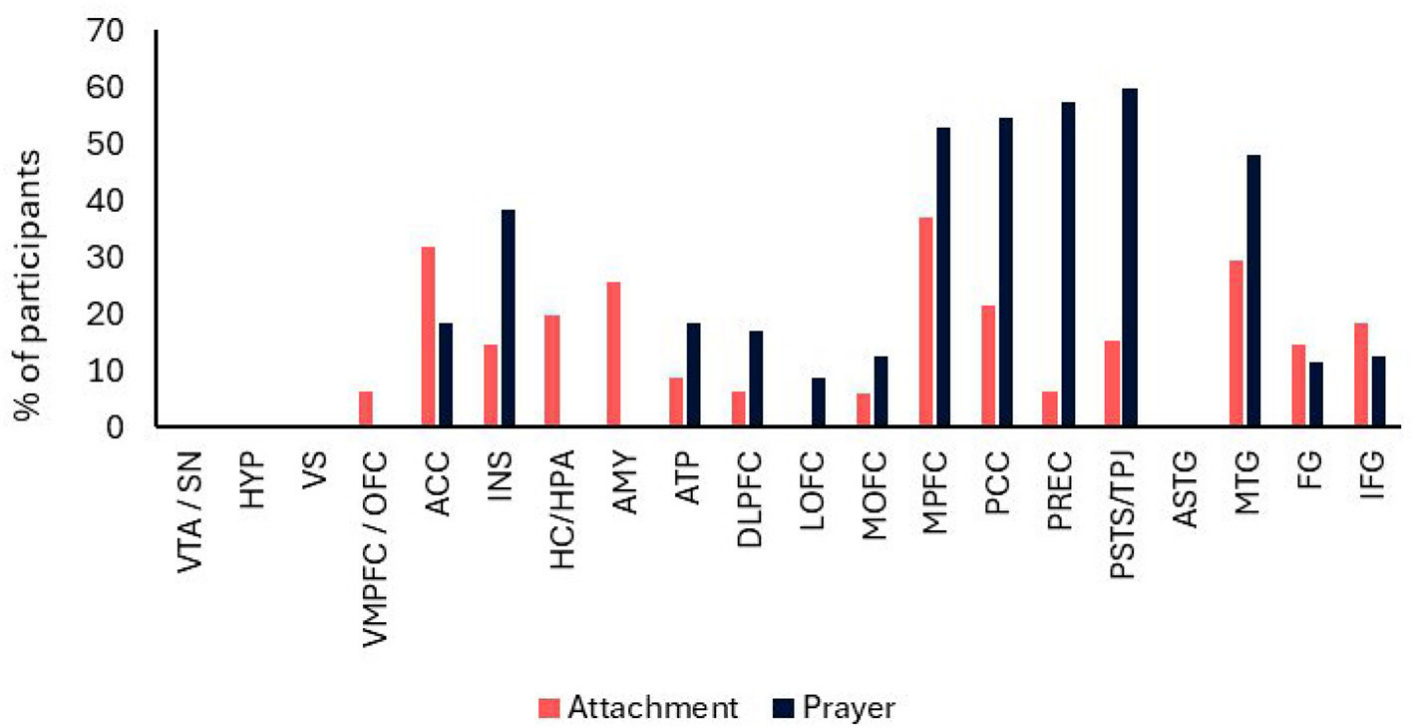
Activation of neural areas during prayer and attachment main effects as a percentage of the total number of participants observed for each region of interest. Approach Module, HYP, hypothalamus; SN, substantia nigra; VMPFC, ventromedial prefrontal cortex; OFC, orbitofrontal cortex; VS, ventral striatum; VTA, ventral tegmental area; Aversion Module, ACC, anterior cingulate cortex; INS, insula; HC/HPA, hippocampus / hypothalamic–pituitary–adrenal axis; AMY, amygdala; ATP, adenosine triphosphate; emotion (self-)regulation module, DLPFC, dorsolateral prefrontal cortex; LOFC, lateral orbitofrontal cortex; mentalizing module, MPFC, medial prefrontal cortex; PCC, posterior cingulate cortex; PREC, precuneus; PSTS/TPJ, posterior superior temporal sulcus/temporoparietal junction; ASTG, anterior superior temporal gyrus; FG, fusiform gyrus; additional areas, IFG, inferior frontal gyrus. MOFC, medial orbitofrontal cortex; MTG, middle temporal gyrus. Areas belonging to each module of attachment were adopted from two prior studies reviewing the neuroscience of attachment (White et al., 2020; Long et al., 2020).

We hypothesized that the neural correlates of prayer would correspond with those implicated in the mentalizing module of attachment, which includes the medial prefrontal cortex, precuneus, posterior superior temporal gyrus, temporo-parietal junction, and posterior cingulate, as well as the anterior superior temporal and fusiform gyrus (Vrticka, 2017; White et al., 2020; Long et al., 2020). Mentalization involves specific aspects of social cognition, broadly defined as the neurocognitive process of interpreting the behaviors of others. Social cognition has been further subdivided into social perception, understanding, and decision-making, with mentalizing behaviors primarily involved in social understanding (Arioli et al., 2018). The widespread activations observed in the medial prefrontal cortex, precuneus, posterior superior temporal gyrus, and temporo-parietal junction suggest that multiple regions commonly associated with mentalizing behaviors and theory of mind were engaged during prayer, as shown in Figure 2. This aligns with the definition of mentalizing as imaginative mental activity that interprets others' affective states and dispositions in terms of beliefs, desires, feelings, goals, and needs, as well as with William James' description of prayer as “inward communion or conversation with the power recognized as divine” (Fonagy and Luyten, 2009; Neubauer, 2014; White et al., 2020). Accordingly, believers may attribute different information, mental states, and motivations to God, necessitating the recruitment of the temporo-parietal junction, posterior superior temporal sulcus, precuneus, and medial prefrontal cortex, which are involved in ToM and mentalizing (Frith and Frith, 2012; Arioli et al., 2018). Not all forms of prayer equally engaged neural networks related to social processing, with the effect being most pronounced during improvised prayers (Schjoedt et al., 2009).

Included studies found enhanced activity in areas associated with resting state activity, commonly referred to as the DMN. This area shows extensive overlap with the mentalizing network (Neubauer, 2014; Silveira et al., 2015; Schjoedt et al., 2009). It has been suggested that the DMN facilitates social processing similar to the aforementioned functionality, reflecting the importance of contemplating social interactions or their absence (Schilbach et al., 2008). We would like to extend this interpretation by noting that the DMN has also been associated with the maintenance of certain aspects of internal working models (White et al., 2020; Alves et al., 2019). In keeping with attachment theory, the internal working models of the self and others are formed through early experiences with primary caregivers, primarily based on their availability and emotional sensitivity (Laurita et al., 2019). These working models were thought to remain stable after early childhood, but current research using both implicit and explicit measures suggests that they can be altered over time, as previously mentioned (Buchheim et al., 2012b; Dugan et al., 2024). In line with observed DMN activity, prayer may engage these internal working models of attachment, providing preliminary neuroscientific evidence for the connection between prayer to God and attachment-related goals, such as seeking a safe haven or departing from an internalized secure base from which to explore the world (Granqvist and Kirkpatrick, 2013; Cherniak et al., 2021; Hall et al., 2009; Kirkpatrick, 2005). Supporting this notion, individuals are more inclined to pray during unexpected aversive experiences (Lucchetti et al., 2021; Thomas and Barbato, 2020; Szałachowski and Tuszyńska-Bogucka, 2021; Sinding Bentzen, 2021). Such findings may indicate an attachment-related coping mechanism, consistent with the idea that the attachment system is primarily activated during negative circumstances (George and West, 2001).

Two observations did not align with the hypothesized convergence between the mentalizing module of attachment and prayer. First, prayer did not reveal significant interactions within the fusiform gyrus and anterior superior temporal gyrus. These areas relate to the interpretation of body language and facial expressions, supported by the extrastriate body area, the fronto-insular-temporal network, and the fusiform gyrus (Amoruso et al., 2011). Conceivably, perceived interaction with a supernatural entity precludes neural enhancement in areas related to the perceptual elements of social cognition (Arioli et al., 2018; Amoruso et al., 2011). Evidence from one of the included prayer studies indeed exhibited overlap between prayer and the DMN. An important caveat is that comparing prayer with speaking to a loved one revealed smaller areas of enhanced neural recruitment (Neubauer, 2014). Similar to different aspects of theory of mind, this might indicate that prayer can be differentiated from other forms of social behavior in terms of the additional neural areas that need not be engaged (Molenberghs et al., 2016). Secondly, it is noteworthy that the mentalizing module of attachment was less frequently implicated in the main effects of attachment compared to prayer. Nonetheless, prior studies indicate that mentalizing areas are significant to attachment experiences—findings that cannot be easily dismissed (Fonagy et al., 2023; Baskak et al., 2020). The disparity may have resulted from utilizing negative attachment stimuli such as the AAP or the threat primes (Labek et al., 2016; Lemche et al., 2006; Nash et al., 2014). These stimuli would not only activate neural areas pertaining to the mentalizing module of attachment but might also enhance other neural correlates in relation to the push-pull mechanism that situates mentalizing behaviors in the broader context of aversion, approach, and emotion (self-)regulation modules of attachment (Vrtička and Vuilleumier, 2012). Our interpretation is supported by the greater incidence of the amygdala in attachment compared to prayer studies, suggesting a greater fear response obtained through negative stimuli (Šimić et al., 2021; Lemche et al., 2006). In addition, pleasant stimuli could relate to enhanced recruitment in mentalizing areas, consistent with a primary attachment strategy observed in secure individuals (Galynker et al., 2012; Petrowski et al., 2019; Liu et al., 2017). If true, the prayer conditions might have been perceived as more engaging, resulting in greater recruitment of the mentalizing areas compared to the attachment stimuli. Although an aversive stimulus could possibly activate mentalizing regions in anxious participants due to their hypervigilant strategy, this effect might have been mitigated by the inclusion of avoidant and securely attached individuals in the main effects—despite initial hypervigilance in avoidant individuals to identify attachment information (Canterberry and Gillath, 2013). A complicating factor in this regard is that attachment studies recruited a significant number of insecurely attached individuals. The fact that non-clinical populations are characterized by more secure attachment styles is estimated to influence reported outcomes (Bakermans-Kranenburg and van IJzendoorn, 2009; van IJzendoorn and Bakermans-Kranenburg, 1996).

### Approach and aversion modules

The approach and aversion modules were expected to show enhanced activation only to a slight degree. The reason being that, although prayer does not necessitate the visible presence of another human being, a posture of openness to the possibility of (mental) interaction with God might require imaginative approach behaviors. Conversely, we hypothesized that participants with a negative God image would recruit the aversion module. However, the God images of participants included in the prayer studies were not assessed, warranting caution in making definitive claims. None of the approach-related areas was shown to be reliably activated in both the attachment and prayer conditions, with the exception of a prayer study that assessed only one Catholic bishop and a record on attachment using both pleasant and unpleasant pictures, as mentioned in Figure 2. Although the second study observed medial orbital frontal cortical activations, this was interpreted by the researchers as an evaluation of decision outcomes (Vrticka et al., 2013; Silveira et al., 2015). Despite not being implicated within the postulated approach module, three prayer studies reported enhanced activations in the caudate nucleus (Galanter et al., 2017; Beauregard and Paquette, 2006; Schjødt et al., 2008). The caudate has previously been identified as important in happiness as well as maternal and passionate love, and it is functionally associated with the limbic system through the ventral striatum, which includes the ventral caudate (Damasio, 2012; Bartels, 2006; Bartels and Zeki, 2004; Shih et al., 2022). Moreover, ventral and dorsal regions of the caudate play distinct roles in processing immediate and future rewards, respectively, with the dorsal caudate, for example, showing engagement during the assessment of future monetary rewards within social contexts (Tricomi et al., 2004; Duarte et al., 2020; Driscoll et al., 2025). In line with the included studies, we interpret the implication of the dorsal caudate as reflecting anticipation of future rewards during the experience of interpersonal interaction with God, indicating trust in God to reciprocate prayer (Schjødt et al., 2008; Graff-Radford et al., 2017). These connections with love and (future) reward may align with the literature on attachment. First, two studies reported activity in the caudate nucleus as a main effect of watching a picture of a participant's mother and the AAP (Galynker et al., 2012; Petrowski et al., 2019; Liu et al., 2017). Recruitment of the caudate during the latter might result from depictions of a goal-corrected partnership within the attachment-caregiving dyad (Bowlby, 1982). Importantly, greater insecurity was related to enhanced left caudal activations as well, indicating augmented reward processing for employing a secondary strategy of “clinging” to attachment figures to assuage or prevent distress (Yaseen et al., 2016). Although not within the postulated approach network, these regions might provide some indication of expected social reciprocity in both prayer and attachment behaviors.

Neural correlates associated with the aversion module of attachment displayed widespread differences between attachment and prayer. First, a greater incidence of the amygdala, anterior cingulate cortex, and hippocampus during the attachment tasks was observed. The amygdala is mainly involved in fear processing, whereas the hippocampal and anterior cingulate activations may reflect experiences of social pain and emotional recall, respectively (Buchheim et al., 2012a; Acosta et al., 2018; Bracht et al., 2022; Quirin et al., 2010; Buchheim et al., 2012b; Norman et al., 2015; Apkarian et al., 2005). We attribute these differences to the negative nature of the attachment stimuli employed, as well as to a purportedly greater number of insecure individuals within attachment studies. Second, the insula and anterior temporal pole, designated as part of the aversion module of attachment, were primarily recruited during prayer. This was unexpected, as prayer studies did not employ emotionally arousing stimuli, consistent with decreased involvement of the amygdala. In addition, the samples in prayer studies likely consisted of a greater number of securely attached individuals (Bakermans-Kranenburg and van IJzendoorn, 2009). In line with observations from the attachment studies included in this review, we therefore propose that insular involvement during prayer reflects interoceptive awareness, more effective emotion recognition, and improved emotion regulation (Galanter et al., 2017; Petrowski et al., 2019; Menon and Uddin, 2010; Molnar-Szakacs and Uddin, 2022; Terasawa et al., 2015; Damasio et al., 2013). Although insular enhancement indeed depends on attachment style, its role may also include regulating the level of access to information entering (un)conscious thought, self-reflective behavior, and salience detection, in addition to revealing an aversive response (White et al., 2020; Long et al., 2020; Menon and Uddin, 2010; Terasawa et al., 2015; Chong et al., 2017; Gibson, 2024; Modinos et al., 2009). In accordance with regions implicated in the mentalizing module of attachment, stronger insular engagement was found in anxious individuals, potentially indicating better access to attachment information, suggestive of a hypervigilant strategy (Lemche et al., 2006; Dewall et al., 2012; Vrticka et al., 2008; Vrtička et al., 2014). Consequently, the hyperarousal strategy typical of attachment avoidance was demonstrated by deactivations in the insula during social rejection, alongside decreased gray matter volume in the anterior insula, with the exception of insular activity in a priming study, as discussed in the mentalizing paragraph (Dewall et al., 2012; Canterberry and Gillath, 2013; Acosta et al., 2018). Therefore, our findings indicate that the insula is not only central to the aversion module of attachment but may also contribute to interoceptive awareness, access to emotional information in response to positive attachment experiences, salience detection, and self-reflective behavior, alongside its previously established role in processing social and physical pain (Yaseen et al., 2016; Petrowski et al., 2019; Kross et al., 2011; Uddin et al., 2017; Coan et al., 2006; Eisenberger et al., 2011). In brief, the neural correlates of the aversion module show mixed results in their overlap with prayer, with insular recruitment in prayer studies serving one of the aforementioned functions.

### Emotion (self-)regulation module

The neural correlates of the self-regulation module were expected to be mobilized during prayer and attachment relationships. This expectation was based on prior studies that associated prayer with improved self-regulation, similar to various types of meditation (McCullough and Carter, 2013; Friese and Wänke, 2014; Tang et al., 2015, 2014). Although the self-regulation strategies employed to discard emotionally neutral events might be functionally distinct from those used to regulate emotions in attachment experiences, neural correspondence between prayer and attachment in these areas could signify a shared mechanism. This is especially true for improvised prayers that more closely reflect attachment experiences (Schjoedt et al., 2009). Two studies reported enhanced recruitment of the dorsolateral prefrontal cortex after contrasting formalized and improvised prayers, implying conscious monitoring of thoughts (Schjoedt et al., 2009; Azari et al., 2001; Raccah et al., 2021; McIntosh et al., 1999). Differences across prayer types likely indicate variations in religious experiences and their corresponding neuropsychological correlates, as well as the fact that formalized prayers are more prone to habituation effects. However, several outcomes suggest that formalized prayers might similarly recruit mentalizing areas (Silveira et al., 2015). Notably, in two prayer records, the condition of reference exhibited enhanced dorsolateral prefrontal and lateral orbitofrontal cortex activations, resulting from the cognitive control required when altering a habitual practice by praying to Santa Claus instead of God (Schjoedt et al., 2009; Elmholdt et al., 2017; Hertrich et al., 2021). In attachment studies, the dorsolateral prefrontal cortex was consistently involved in attachment conditions that evoked negative emotions (Vrtička et al., 2012; Buchheim et al., 2008; Lemche et al., 2006; Buchheim et al., 2013; Krause et al., 2016; Buchheim et al., 2016). This effect was most pronounced in unresolved participants, whereas unresolved borderline patients did not engage the dorsolateral prefrontal cortex, signifying their inability to self-regulate during heightened distress (Ochsner and Gross, 2005). Currently, we cannot determine whether conscious monitoring of thoughts during formalized prayer bears a functional similarity to the self-regulation module of attachment, since it is unclear to what degree the attachment system was engaged during structured forms of prayer and whether similar mechanisms were at play. In addition, both improvised prayer conditions and positive attachment stimuli did not reveal significant activations in the hypothesized areas. In brief, there are no substantive differences between prayer and attachment regarding the recruitment of areas associated with the emotion (self-)regulation module.

### Other faith traditions

Although not discussed in the main body of our study, three of the included studies investigated prayer in other faith traditions (Baykara et al., 2023; AlMahrouqi and Mostafa, 2023; Perez-Diaz et al., 2023). Islamic prayer was explored through structural MRI, highlighting different corpus callosum shapes compared to non-praying controls. The observed changes were not ascribed to specific behavioral or psychological differences. Furthermore, the corpus callosum lies beyond the scope of the neural substrates associated with attachment (Baykara et al., 2023). A second study employing fMRI found that listening to Quran recitals by Muslims, compared to the same condition in non-Muslims, exhibited enhanced activation in the anterior cingulate cortex and insula, as well as the medial prefrontal and superior temporal cortex. From an attachment perspective, the amygdala might be related to aversive experiences, while the latter two regions are implicated in social interactions and ToM, coinciding with findings reported during Christian prayer partial (Ghobary Bonab et al., 2013; AlMahrouqi and Mostafa, 2023). This correspondence between Christian and Muslim prayer was also supported by two EEG recordings that were later identified, revealing increased gamma frequency in parietal and occipital regions (Surwillo and Hobson, 1978; Doufesh et al., 2014, 2012). Future research is warranted to compare Quranic recitals with more formal types of Christian prayer. Conversely, praying in Sahaja Yoga meditation was associated with inner concentration, reduced social judgment, and decreased mentalizing, evident from medial prefrontal deactivations (Perez-Diaz et al., 2023). This observation further supports the notion that religious practices across cultures may vary in terms of functionality and their neural underpinnings (Schjoedt et al., 2009; Newberg, 2014; Wuthnow, 2008).

### Limitations and future research

Several strengths must be acknowledged. This is the first systematic review devoted to comparing the neuroscience of prayer and attachment relationships, as well as the first systematic review to compare a specific social cognitive function with a religious experience. As such, this systematic review provides empirically verifiable hypotheses on the mechanisms by which mental health and spirituality may interact.

When considering the extant body of research on attachment and prayer, a primary limitation to address is the relatively small sample sizes employed, particularly in prayer studies. Consequently, the results might be affected by insufficient statistical power, biased effect sizes, and inconsistent findings (Button et al., 2013). Moreover, our inability to analyze the data in a meta-analytic fashion could have resulted in a loss of precision regarding our overview of the neural correlates of prayer and attachment Figure 2. Secondly, the generalizability of our findings is limited by the predominance of female participants in the included populations. This issue was especially problematic in articles using implicit attachment measures, a concern also highlighted in an earlier systematic review (Eilert and Buchheim, 2023). Notably, only one sMRI study conducted in China examined sex differences. The authors observed differences in middle occipital gyrus volumes in relation to attachment avoidance and the biological sex of the participants. This result was attributed to the one-child policy, particularly the overprotection of male children (Zhang et al., 2011). Nonetheless, the extent to which our findings regarding attachment can be extrapolated to male populations remains uncertain. Thirdly, our scope was limited by the inclusion of studies from Europe and North America, with the exception of three attachment-related records from Asia. This increases the risk of selection bias resulting from cultural influences (Wuthnow, 2008). Finally, the assumption that prayer in an fMRI or neuroimaging environment corresponds to everyday prayer practices has been challenged by prior research in a mock-fMRI setting (Ladd et al., 2015). Although some of the included studies used debriefing protocols to assess the validity of prayer experiences, this was not standard practice and may have resulted in measurement bias. Future research within the neuroscience of prayer should include such questionnaires and aim to facilitate authentic prayer experiences (Ladd et al., 2015).

With much yet to uncover in the social neuroscience of attachment and the neuroscience of prayer, there are a few considerations for future research. First, none of the implicit attachment studies separately measured the activated attachment system in preoccupied individuals or compared implicitly assessed preoccupation with avoidance (George and West, 2001). Such findings would be crucial for distinguishing patterns of hypo- and hyperarousal in attachment literature more broadly and for the relationship between prayer and attachment more specifically (Long et al., 2020). Importantly, secondary strategies of attachment were primarily assessed through explicit measures that reflect more conscious forms of processing, necessitating more research in this area (Yaseen et al., 2016). In the previous sections, observed divergence in the mentalizing and aversion modules was partly attributed to the negative influence of adult attachment stimuli. It should be examined whether incorporating negative stimuli prior to prayer yields a greater correspondence between prayer and attachment outcomes. Subsequently, these effects may be assessed at the level of main effects, as well as at the level of Correlates for Separate Attachment Styles (CSAS) and Differences Between Attachment Styles (DBAS), as mentioned in Tables 3–7. If convergence is indeed observed, it may highlight a physiological return to homeostasis through internal working models recruited during prayer, depending on one's representation of others and of God (Granqvist and Kirkpatrick, 2013; Porges, 2022). In addition, neuroimaging research offers the unique opportunity to bypass explicit beliefs and investigate whether individuals with insecure attachment styles implicitly *compensate* for their insecurity by having a more secure relationship with God, or whether there is *correspondence* between attachment to God and to others (Cherniak et al., 2021). Finally, future studies are warranted to explore how different religious practices within one religion relate to one another, for instance, investigating glossolalia: a type of prayer not addressed in this study (Walter, 2021; Chouiter and Annoni, 2017; Newberg et al., 2006).

To conclude, this systematic review lends further support to the previously established role of the default mode network in prayer, while adding that this network corresponds with the mentalizing module of attachment, involving the recruitment of internal working models of attachment. Furthermore, no significant differences were observed when comparing prayer to attachment within neural areas pertaining to the approach and self-regulation modules, with caudal activations potentially reflecting experienced social reciprocity related to trust in others and trust in God. A pronounced disparity between attachment and prayer was observed in areas of the aversion module. It was particularly unexpected that the insula exhibited enhanced activation during prayer practices: a finding attributed to the potentially multifaceted nature of the insula beyond its previously established role in aversive experiences. Future research is warranted to verify the hypothesized interaction between attachment and prayer for individuals with different attachment styles. Subsequently, the outcomes may inspire randomized controlled trials (RCTs) on the impact of prayer on attachment, to develop strategies for integrating religious beliefs in psychotherapy and ultimately improve therapeutic outcomes for clients.

## Data Availability

The raw data supporting the conclusions of this article will be made available by the authors, without undue reservation.

## References

[B1] Abraham A. von Cramon D. Y. Schubotz R. I. (2008). Meeting George Bush versus meeting Cinderella: the neural response when telling apart what is real from what is fictional in the context of our reality. J. Cogn. Neurosci. 20, 965–976. 10.1162/jocn.2008.20059 18211244

[B2] Acosta H. Jansen A. Nuscheler B. Kircher T. A. (2018). voxel-based morphometry study on adult attachment style and affective loss. Neuroscience 392, 219–229. 10.1016/j.neuroscience.2018.06.045 30005995

[B3] Ainsworth M. D. S. Blehar M. C. Waters E. Wall S. N. (2015). Patterns of Attachment: A Psychological Study of the Strange Situation. New York: Psychology Press, 466.

[B4] Aletti M. (2005). Religion as an illusion: prospects for and problems with a psychoanalytical model. Arch Psychol Relig. 27, 1–18. 10.1163/008467206774355367

[B5] AlMahrouqi K. Mostafa M. M. (2023). Neural correlates of Quran recitals: a functional magnetic resonance imaging (fMRI) analysis. Multimed. Tools Appl. 82, 47719–47732. 10.1007/s11042-023-15588-3

[B6] Alves P. N. Foulon C. Karolis V. Bzdok D. Margulies D. S. Volle E. . (2019). An improved neuroanatomical model of the default-mode network reconciles previous neuroimaging and neuropathological findings. Commun Biol. 2:370. 10.1038/s42003-019-0611-3 31633061 PMC6787009

[B7] Amoruso L. Couto J. B. Ibanez A. (2011). Beyond Extrastriate Body Area (EBA) and Fusiform Body Area (FBA): context integration in the meaning of actions. Front. Hum. Neurosci. 5:124. 10.3389/fnhum.2011.00124 22053154 PMC3205482

[B8] Anderson J. W. Nunnelley P. A. (2016). Private prayer associations with depression, anxiety and other health conditions: an analytical review of clinical studies. Postgrad. Med. 128, 635–641. 10.1080/00325481.2016.1209962 27452045

[B9] Apkarian A. V. Bushnell M. C. Treede R. D. Zubieta J. K. (2005). Human brain mechanisms of pain perception and regulation in health and disease. Eur. J. Pain Lond. Engl. 9, 463–484. 10.1016/j.ejpain.2004.11.001 15979027

[B10] Arioli M. Crespi C. Canessa N. (2018). Social cognition through the lens of cognitive and clinical neuroscience. Biomed Res. Int. 2018:4283427. 10.1155/2018/4283427 30302338 PMC6158937

[B11] Azari N. P. Missimer J. Seitz R. J. (2005). Religious experience and emotion: evidence for distinctive cognitive neural patterns. Int. J. Psychol. Relig. 15, 263–281. 10.1207/s15327582ijpr1504_1

[B12] Azari N. P. Nickel J. Wunderlich G. Niedeggen M. Hefter H. Tellmann L. . (2001). Neural correlates of religious experience. Eur. J. Neurosci. 13, 1649–1652. 10.1046/j.0953-816x.2001.01527.x 11328359

[B13] Bakermans-Kranenburg M. J. van IJzendoorn M. H. (2009). The first 10,000 Adult Attachment Interviews: distributions of adult attachment representations in clinical and non-clinical groups. Attach. Hum. Dev. 11, 223–263. 10.1080/14616730902814762 19455453

[B14] Bartels A. (2006). Neurobiological foundations of mate choice and love. Sexuologie 13, 118–129. 10.61387/S.2006.24.15

[B15] Bartels A. Zeki S. (2000). The neural basis of romantic love. Neuroreport 11, 3829–3834. 10.1097/00001756-200011270-00046 11117499

[B16] Bartels A. Zeki S. (2004). The neural correlates of maternal and romantic love. Neuroimage 21, 1155–1166. 10.1016/j.neuroimage.2003.11.003 15006682

[B17] Baskak B. Klr Y. Sedes N. Kuşman A. Türk E. G. Baran Z. . (2020). Attachment style predicts cortical activity in temporoparietal junction (TPJ): an fNIRS study using a theory of mind (ToM) task in healthy university students. J. Psychophysiol. 34, 99–109. 10.1027/0269-8803/a000240 33840879 PMC8034263

[B18] Baykara S. Baykara M. Atmaca M. (2023). Regular Islamic prayers have different corpus callosum: a shape analysis study. Egypt J. Neurol. Psychiatry Neurosurg. 59:81. 10.1186/s41983-023-00683-x

[B19] Beauchamp M. S. (2015). The social mysteries of the superior temporal sulcus. Trends Cogn. Sci. 19, 489–490. 10.1016/j.tics.2015.07.002 26208834 PMC4556565

[B20] Beauregard M. Paquette V. (2006). Neural correlates of a mystical experience in Carmelite nuns. Neurosci. Lett. 405, 186–190. 10.1016/j.neulet.2006.06.060 16872743

[B21] Beauregard M. Paquette V. E. E. G. (2008). activity in Carmelite nuns during a mystical experience. Neurosci. Lett. 444, 1–4. 10.1016/j.neulet.2008.08.028 18721862

[B22] Benetti S. McCrory E. Arulanantham S. De Sanctis T. McGuire P. Mechelli A. . (2010). Attachment style, affective loss and gray matter volume: a voxel-based morphometry study. Hum. Brain Mapp. 31, 1482–1489. 10.1002/hbm.20954 20127871 PMC6870872

[B23] Bernheim D. Buchheim A. Domin M. Mentel R. Lotze M. (2022). Neural correlates of attachment representation in patients with borderline personality disorder using a personalized functional magnet resonance imaging task. Front. Hum. Neurosci. 16:810417. 10.3389/fnhum.2022.810417 35280201 PMC8908102

[B24] Bettmann J. E. Jasperson R. A. (2010). Anxiety in adolescence: the integration of attachment and neurobiological research into clinical practice. Clin. Soc. Work J. 38, 98–106. 10.1007/s10615-009-0235-z

[B25] Beutel M. E. Stark R. Pan H. Silbersweig D. Dietrich S. (2010). Changes of brain activation pre- post short-term psychodynamic inpatient psychotherapy: an fMRI study of panic disorder patients. Psychiatry Res Neuroimaging. 184, 96–104. 10.1016/j.pscychresns.2010.06.005 20933374

[B26] Black S. W. Pössel P. Jeppsen B. D. Tariq A. Rosmarin D. H. (2015). Poloma and Pendleton's (1989) Prayer Types Scale in Christian, Jewish, and Muslim praying adults: one scale or a family of scales? Psychol Relig Spiritual. 7, 205–216. 10.1037/rel0000018

[B27] Bowlby J. (1980). “Attachment and loss: Vol. 3,” in Loss, Sadness and Depression (London: Hogarth Press).

[B28] Bowlby J. (1982). Attachment and loss: retrospect and prospect. Am. J. Orthopsychiatry. 52, 664–678. 10.1111/j.1939-0025.1982.tb01456.x 7148988

[B29] Braam A. W. Deeg D. J. H. Poppelaars J. L. Beekman A. T. F. van Tilburg W. (2007). Prayer and depressive symptoms in a period of secularization: patterns among older adults in the Netherlands. Am. J. Geriatr. Psychiatry. 15, 273–81. 10.1097/JGP.0b013e31802d0ae8 17384312

[B30] Bracht T. Denier N. Wallimann M. Walther S. Mertse N. Breit S. . (2022). Hippocampal volume and parahippocampal cingulum alterations are associated with avoidant attachment in patients with depression. J Affect Disord Rep. 10:100435. 10.1016/j.jadr.2022.100435

[B31] Bradshaw M. Ellison C. G. Marcum J. P. (2010). Attachment to God, images of God, and psychological distress in a nationwide sample of Presbyterians. Int. J. Psychol. Relig. 20, 130–147. 10.1080/10508611003608049 23329878 PMC3545685

[B32] Bramer W. M. Giustini D. de Jonge G. B. Holland L. Bekhuis T. (2016). De-duplication of database search results for systematic reviews in EndNote. J. Med. Libr. Assoc. 104, 240–3. 10.3163/1536-5050.104.3.014 27366130 PMC4915647

[B33] Breslin M. J. Lewis C. A. (2008). Theoretical models of the nature of prayer and health: a review. Ment. Health Relig. Cult. 11, 9–21. 10.1080/136746707014914498795874

[B34] Brewer J. Garrison K. Whitfield-Gabrieli S. (2013). What about the “self” is processed in the posterior cingulate cortex? Front. Hum. Neurosci. 7:647. 10.3389/fnhum.2013.00647 24106472 PMC3788347

[B35] Bruneau E. G. Pluta A. Saxe R. (2012). Distinct roles of the ‘Shared Pain' and ‘Theory of Mind' networks in processing others' emotional suffering. Neuropsychologia 50, 219–231. 10.1016/j.neuropsychologia.2011.11.008 22154962

[B36] Buchheim A. Erk S. George C. Kächele H. Kircher T. Martius P. . (2008). Neural correlates of attachment trauma in borderline personality disorder: a functional magnetic resonance imaging study. Psychiatry Res. Neuroimag. 163, 223–235. 10.1016/j.pscychresns.2007.07.001 18635342

[B37] Buchheim A. Erk S. George C. Kächele H. Martius P. Pokorny D. . (2016). Neural response during the activation of the attachment system in patients with borderline personality disorder: an fMRI study. Front. Hum. Neurosci. 10:389. 10.3389/fnhum.2016.00389 27531977 PMC4969290

[B38] Buchheim A. Erk S. George C. Kächele H. Ruchsow M. Spitzer M. . (2006). Measuring attachment representation in an fMRI environment: a pilot study. Psychopathology 39, 144–152. 10.1159/000091800 16531690

[B39] Buchheim A. George C. West M. (2003). The Adult Attachment Projective (AAP) - Psychometric properties and new research results. PPmP Psychother Psychosom. Med. Psychol. 53, 419–426. 10.1055/s-2003-42170 14528412

[B40] Buchheim A. Labek K. Walter S. Viviani R. A. (2013). clinical case study of a psychoanalytic psychotherapy monitored with functional neuroimaging. Front. Hum. Neurosci. 7:677. 10.3389/fnhum.2013.00677 24167481 PMC3805951

[B41] Buchheim A. Viviani R. Kessler H. Kächele H. Cierpka M. Roth G. . (2012a). Neuronal changes in chronic depressed patients during psychoanalytic psychotherapy. Functional magnetic resonance imaging study with an attachment paradigm. Psychotherapeut. 57, 219–226. 10.1007/s00278-012-0909-9

[B42] Buchheim A. Viviani R. Kessler H. Kächele H. Cierpka M. Roth G. . (2012b). Changes in prefrontal-limbic function in major depression after 15 months of long-term psychotherapy. PLoS ONE 7:e33745. 10.1371/journal.pone.0033745 22470470 PMC3314671

[B43] Button K. S. Ioannidis J. P. A. Mokrysz C. Nosek B. A. Flint J. Robinson E. S. J. . (2013). Power failure: why small sample size undermines the reliability of neuroscience. Nat. Rev. Neurosci. 14, 365–376. 10.1038/nrn3475 23571845

[B44] Canterberry M. Gillath O. (2013). Neural evidence for a multifaceted model of attachment security. Int. J. Psychophysiol. 88, 232–240. 10.1016/j.ijpsycho.2012.08.013 22940284

[B45] Captari L. E. Hook J. N. Hoyt W. Davis D. E. McElroy-Heltzel S. E. Worthington J.r. . (2018). Integrating clients' religion and spirituality within psychotherapy: a comprehensive meta-analysis. J. Clin. Psychol. 74, 1938–1951. 10.1002/jclp.22681 30221353

[B46] Captari L. E. Sandage S. J. Vandiver R. A. (2022). Spiritually integrated psychotherapies in real-world clinical practice: synthesizing the literature to identify best practices and future research directions. Psychotherapy 59, 307–320. 10.1037/pst0000407 34843316

[B47] Carter C. S. van Veen V. (2007). Anterior cingulate cortex and conflict detection: an update of theory and data. Cogn. Affect. Behav. Neurosci. 7, 367–379. 10.3758/CABN.7.4.367 18189010

[B48] Cascio C. N. Konrath S. H. Falk E. B. (2013). Narcissists' social pain seen only in the brain. Soc. Cogn. Affect. Neurosci. 10, 335–341. 10.1093/scan/nsu072 24860084 PMC4350489

[B49] Chen Y. VanderWeele T. J. (2018). Associations of religious upbringing with subsequent health and well-being from adolescence to young adulthood: an outcome-wide analysis. Am. J. Epidemiol. 187, 2355–2364. 10.1093/aje/kwy142 30215663 PMC6211237

[B50] Cherniak A. D. Mikulincer M. Shaver P. R. Granqvist P. (2021). Attachment theory and religion. Curr. Opin. Psychol. 40, 126–130. 10.1016/j.copsyc.2020.08.020 33075624

[B51] Chong J. S. X. Ng G. J. P. Lee S. C. Zhou J. (2017). Salience network connectivity in the insula is associated with individual differences in interoceptive accuracy. Brain Struct. Funct. 222, 1635–1644. 10.1007/s00429-016-1297-7 27573028

[B52] Chouiter L. Annoni J. M. (2017). Glossolalia and aphasia: related but different worlds. Front. Neurol. Neurosci. 42, 96–105. 10.1159/000475694 29151094

[B53] Christopoulos G. I. Tobler P. N. Bossaerts P. Dolan R. J. Schultz W. (2009). Neural correlates of value, risk, and risk aversion contributing to decision making under risk. J. Neurosci. 29, 12574–12583. 10.1523/JNEUROSCI.2614-09.2009 19812332 PMC2794196

[B54] Coan J. A. (2008). “Toward a neuroscience of attachment,” in Handbook of Attachment: Theory, Research, and Clinical Applications, eds. J. Cassidy, and P. R. Shaver (New York, NY: The Guilford Press), 241–65.

[B55] Coan J. A. Schaefer H. S. Davidson R. J. (2006). Lending a hand: social regulation of the neural response to threat. Psychol. Sci. 17, 1032–1039. 10.1111/j.1467-9280.2006.01832.x 17201784

[B56] Cook C. (2011). The faith of the psychiatrist. Ment. Health Relig. Cult. 14, 9–17. 10.1080/13674671003622673

[B57] Counted V. (2016). God as an attachment figure: a case study of the God attachment language and God concepts of anxiously attached Christian youths in South Africa. J. Spiritual Ment. Health 18, 316–346. 10.1080/19349637.2016.1176757

[B58] Currier J. M. Foster J. D. Witvliet C. Abernethy A. D. Root Luna L. M. Schnitker S. A. . (2019). Spiritual struggles and mental health outcomes in a spiritually integrated inpatient program. J. Affect. Disord. 249, 127–135. 10.1016/j.jad.2019.02.012 30772739

[B59] Damasio A. Damasio H. Tranel D. (2013). Persistence of feelings and sentience after bilateral damage of the insula. Cereb. Cortex. 23, 833–846. 10.1093/cercor/bhs077 22473895 PMC3657385

[B60] Damasio A. R. (2012). “A note on the neurobiology of emotions,” in Altruism and Altruistic Love: Science, Philosophy, and Religion in Dialogue (Oxford: Oxford University Press).

[B61] De Filippi E. Escrichs A. Càmara E. Garrido C. Marins T. Sánchez-Fibla M. . (2022). Meditation-induced effects on whole-brain structural and effective connectivity. Brain Struct. Funct. 227, 2087–2102. 10.1007/s00429-022-02496-9 35524072 PMC9232427

[B62] Dewall C. N. Masten C. L. Powell C. Combs D. Schurtz D. R. Eisenberger N. I. . (2012). Do neural responses to rejection depend on attachment style? An fMRI study. Soc. Cogn. Affect. Neurosci. 7, 184–192. 10.1093/scan/nsq107 21467049 PMC3277372

[B63] Doufesh H. Faisal T. Lim K. S. Ibrahim F. E. E. G. (2012). spectral analysis on Muslim prayers. Appl. Psychophysiol. Biofeedback. 37, 11–18. 10.1007/s10484-011-9170-1 21965118

[B64] Doufesh H. Ibrahim F. Ismail N. A. Ahmad W. A. W. (2014). Effect of Muslim prayer (Salat) on α electroencephalography and its relationship with autonomic nervous system activity. J. Altern. Complement. Med. 20, 558–562. 10.1089/acm.2013.0426 24827587 PMC4086364

[B65] Drevets W. C. (2001). Neuroimaging and neuropathological studies of depression: implications for the cognitive-emotional features of mood disorders. Curr. Opin. Neurobiol. 11, 240–249. 10.1016/S0959-4388(00)00203-8 11301246

[B66] Driscoll M. E. Bollu P. C. Tadi P. (2025). “Neuroanatomy, nucleus caudate,” in StatPearls (Treasure Island (FL): StatPearls Publishing). 32491339

[B67] Duarte I. C. Coelho G. Brito-Costa S. Cayolla R. Afonso S. Castelo-Branco M. . (2020). Ventral caudate and anterior insula recruitment during value estimation of passionate rewarding cues. Front. Neurosci. 14:678. 10.3389/fnins.2020.00678 32848534 PMC7403482

[B68] Dugan K. A. Fraley R. C. Jones J. D. Stern J. A. Shaver P. R. Lejuez C. W. . (2024). Coordination of parent and adolescent attachment across time. Dev. Psychol. 1–18. 10.1037/dev0001835 [Epub ahead of print]. 39480314 PMC12461888

[B69] Eilert D. W. Buchheim A. (2023). Attachment-related differences in emotion regulation in adults: a systematic review on attachment representations. Brain Sci. 13:884. 10.3390/brainsci13060884 37371364 PMC10296607

[B70] Eisenberger N. I. Master S. L. Inagaki T. K. Taylor S. E. Shirinyan D. Lieberman M. D. . (2011). Attachment figures activate a safety signal-related neural region and reduce pain experience. Proc. Natl. Acad. Sci. USA. 108, 11721–11726. 10.1073/pnas.1108239108 21709271 PMC3136329

[B71] Ellison C. G. Bradshaw M. Flannelly K. J. Galek K. C. (2014). Prayer, attachment to God, and symptoms of anxiety-related disorders among U.S. adults. Sociol Relig. 75, 208–233. 10.1093/socrel/srt079

[B72] Elmholdt E. M. Skewes J. Dietz M. Møller A. Jensen M. S. Roepstorff A. . (2017). Reduced pain sensation and reduced BOLD signal in parietofrontal networks during religious prayer. Front. Hum. Neurosci. 11:337. 10.3389/fnhum.2017.00337 28701940 PMC5487465

[B73] Etkin A. Egner T. Kalisch R. (2011). Emotional processing in anterior cingulate and medial prefrontal cortex. Trends Cogn. Sci. 15, 85–93. 10.1016/j.tics.2010.11.004 21167765 PMC3035157

[B74] Flechsig A. Bernheim D. Buchheim A. Domin M. Mentel R. Lotze M. . (2023). One year of outpatient dialectical behavioral therapy and its impact on neuronal correlates of attachment representation in patients with borderline personality disorder using a personalized fMRI task. Brain Sci. 13:1001. 10.3390/brainsci13071001 37508932 PMC10377139

[B75] Fonagy P. Bateman A. W. Lorenzini N. Campbell C. (2014). “Development, attachment, and childhood experience,” in The American Psychiatric Publishing Textbook of Personality Disorders, eds. J. M. Oldham, A. E. Skodol, and D. S. Bender (Arlington, VA, US: American Psychiatric Publishing, Inc), 55–77.

[B76] Fonagy P. Campbell C. Luyten P. (2023). Attachment, mentalizing and trauma: then (1992) and now (2022). Brain Sci. 13:459. 10.3390/brainsci13030459 36979268 PMC10046260

[B77] Fonagy P. Luyten P. A. (2009). developmental, mentalization-based approach to the understanding and treatment of borderline personality disorder. Dev. Psychopathol. 21, 1355–1381. 10.1017/S0954579409990198 19825272

[B78] Fonseka B. A. Jaworska N. Courtright A. MacMaster F. P. MacQueen G. M. (2016). Cortical thickness and emotion processing in young adults with mild to moderate depression: a preliminary study. BMC Psychiatry. 16:8. 10.1186/s12888-016-0750-8 26911621 PMC4765096

[B79] Francis L. Robbins M. Lewis C. (2008). Prayer and psychological health: a study among sixth-form pupils attending Catholic and Protestant schools in Northern Ireland. *Ment. Health Relig*. Cult. 11:9055. 10.1080/13674670701709055

[B80] Friese M. Wänke M. (2014). Personal prayer buffers self-control depletion. J. Exp. Soc. Psychol. 51, 56–59. 10.1016/j.jesp.2013.11.006

[B81] Frith C. D. Frith U. (2012). Mechanisms of social cognition. Annu. Rev. Psychol. 63, 287–313. 10.1146/annurev-psych-120710-100449 21838544

[B82] Froese P. Bonhag R. Uecker J. Andersson M. Upenieks L. (2024). Prayer and mental well-being in the United States: an overview of original and comprehensive prayer data. J. Relig. Health. 63, 4745–4772. 10.1007/s10943-024-02121-5 39245703

[B83] Galante J. Friedrich C. Dawson A. F. Modrego-Alarcón M. Gebbing P. Delgado-Suárez I. . (2021). Mindfulness-based programs for mental health promotion in adults in nonclinical settings: A systematic review and meta-analysis of randomized controlled trials. PLoS Med. 18:e1003481. 10.1371/journal.pmed.1003481 33428616 PMC7799763

[B84] Galanter M. Josipovic Z. Dermatis H. Weber J. Millard M. A. (2017). An initial fMRI study on neural correlates of prayer in members of Alcoholics Anonymous. Am. J. Drug Alcohol Abuse. 43, 44–54. 10.3109/00952990.2016.1141912 27015258

[B85] Galynker I. I. Yaseen Z. S. Katz C. Zhang X. Jennings-Donovan G. Dashnaw S. . (2012). Distinct but overlapping neural networks subserve depression and insecure attachment. Soc. Cogn. Affect. Neurosci. 7, 896–908. 10.1093/scan/nsr074 22037687 PMC3501706

[B86] George C. Solomon J. (1996). Representational models of relationships: links between caregiving and attachment. Infant Ment. Health J. 17, 198–216.

[B87] George C. Solomon J. (2008). “The caregiving system: a behavioral systems approach to parenting,” in Handbook of Attachment: Theory, Research, and Clinical Applications, ed. J. Cassidy, and P. R. Shaver (New York, NY, US: The Guilford Press), 833–56.

[B88] George C. West M. (2001). The development and preliminary validation of a new measure of adult attachment: the adult attachment projective. Attach. Hum. Dev. 3, 30–61. 10.1080/14616730010024771 11708383

[B89] Ghafari M. Nadi T. Bahadivand-Chegini S. Doosti-Irani A. (2022). Global prevalence of unmet need for mental health care among adolescents: a systematic review and meta-analysis. Arch. Psychiatr. Nurs. 36, 1–6. 10.1016/j.apnu.2021.10.008 35094819

[B90] Ghobary Bonab B. Miner M. Proctor M. T. (2013). Attachment to God in Islamic spirituality. J. Muslim Ment. Health. 7:205. 10.3998/jmmh.10381607.0007.205

[B91] Gibson J. E. (2024). Meditation and interoception: a conceptual framework for the narrative and experiential self. Front. Psychol. 15:1393969. 10.3389/fpsyg.2024.1393969 39478794 PMC11521916

[B92] Gkintoni E. Vassilopoulos S. P. Nikolaou G. (2025). Mindfulness-based cognitive therapy in clinical practice: a systematic review of neurocognitive outcomes and applications for mental health and well-being. J Clin Med. 14:1703. 10.3390/jcm14051703 40095733 PMC11900371

[B93] Goyal M. Singh S. Sibinga E. M. S. Gould N. F. Rowland-Seymour A. Sharma R. . (2014). Meditation programs for psychological stress and well-being: a systematic review and meta-analysis. JAMA Intern. Med. 174, 357–368. 10.1001/jamainternmed.2013.13018 24395196 PMC4142584

[B94] Graça L. Brandão T. (2024). Religious/spiritual coping, emotion regulation, psychological well-being, and life satisfaction among university students. J. Psychol. Theol. 52, 342–358. 10.1177/00916471231223920

[B95] Graff-Radford J. Williams L. Jones D. T. Benarroch E. E. (2017). Caudate nucleus as a component of networks controlling behavior. Neurology 89, 2192–2197. 10.1212/WNL.0000000000004680 29070661 PMC5696645

[B96] Granqvist P. (2006). “Religion as a by-product of evolved psychology: the case of attachment and implications for brain and religion research,” in Where God and Science Meet: How Brain and Evolutionary Studies Alter our Understanding of Religion, eds. P. McNamara, E. Harris (Westport, Conn: APA), 105–50.

[B97] Granqvist P. Kirkpatrick L. A. (2013). “Religion, spirituality, and attachment,” in APA Handbook of Psychology, Religion, and Spirituality (Vol 1): Context, Theory, and Research (Washington, DC, US: American Psychological Association), 139–55.

[B98] Grecucci A. Giorgetta C. Bonini N. Sanfey A. G. (2013). Reappraising social emotions: the role of inferior frontal gyrus, temporo-parietal junction and insula in interpersonal emotion regulation. Front. Hum. Neurosci. 7:523. 10.3389/fnhum.2013.00523 24027512 PMC3759791

[B99] Gunderson J. Lyons-Ruth K. (2008). BPD'S interpersonal hypersensitivity phenotype: a gene-environment-developmental model. J Personal Disord. 22, 22–41. 10.1521/pedi.2008.22.1.22 18312121 PMC2596628

[B100] Gunderson J. G. (1996). The borderline patient's intolerance of aloneness: insecure attachments and therapist availability. Am. J. Psychiatry. 153, 752–758. 10.1176/ajp.153.6.752 8633685

[B101] Haddaway N. R. Page M. J. Pritchard C. C. McGuinness L. A. (2022). PRISMA2020: An R package and Shiny app for producing PRISMA 2020-compliant flow diagrams, with interactivity for optimised digital transparency and Open Synthesis. Campbell Syst Rev. 18:e1230. 10.1002/cl2.1230 36911350 PMC8958186

[B102] Hall T. W. Fujikawa A. Halcrow S. R. Hill P. C. Delaney H. (2009). Attachment to God and implicit spirituality: clarifying correspondence and compensation models. J. Psychol. Theol. 37, 227–44. 10.1177/009164710903700401

[B103] Hartwright C. E. Hansen P. C. Apperly I. A. (2016). Current knowledge on the role of the inferior frontal gyrus in theory of mind - a commentary on Schurz and Tholen (2016). Cortex 85, 133–136. 10.1016/j.cortex.2016.10.005 27829498

[B104] Haverkamp E. (2024). The Convergent Neuroscience of Christian Prayer and Attachment Relationships: a Systematic Review [Internet]. Charlottesville, VA: Open Science Foundation. Available online at: https://osf.io/hyzpn

[B105] Hebert R. S. Dang Q. Schulz R. (2007). Religious beliefs and practices are associated with better mental health in family caregivers of patients with dementia: findings from the REACH study. Am. J. Geriatr. Psychiatry. 15, 292–300. 10.1097/01.JGP.0000247160.11769.ab 17158632

[B106] Herpertz S. C. Dietrich T. M. Wenning B. Krings T. Erberich S. G. Willmes K. . (2001). Evidence of abnormal amygdala functioning in borderline personality disorder: a functional MRI study. Biol. Psychiatry. 50, 292–298. 10.1016/S0006-3223(01)01075-7 11522264

[B107] Hertrich I. Dietrich S. Blum C. Ackermann H. (2021). The role of the dorsolateral prefrontal cortex for speech and language processing. Front. Hum. Neurosci. 15:645209. 10.3389/fnhum.2021.645209 34079444 PMC8165195

[B108] Hodge D. R. Gebler-Wolfe M. M. (2022). Understanding technology's impact on youth: Attachment theory as a framework for conceptualizing adolescents' relationship with their mobile devices. Child. Sch. 44, 153–162. 10.1093/cs/cdac007

[B109] Holmes J. Slade A. (2019). The neuroscience of attachment: implications for psychological therapies. Br. J. Psychiatry. 214, 318–319. 10.1192/bjp.2019.7 31088592

[B110] Hou J. Chen X. Liu J. Yao F. Huang J. Ndasauka Y. . (2016). How does adult attachment affect human recognition of love-related and sex-related stimuli: an ERP study. Front. Psychol. 7. 10.3389/fpsyg.2016.00596 27199830 PMC4852297

[B111] Huang H. Nguyen P. T. Schwab N. A. Tanner J. J. Price C. C. Ding M. . (2017). Mapping dorsal and ventral caudate in older adults: method and validation. Front. Aging Neurosci. 9:91. 10.3389/fnagi.2017.00091 28420985 PMC5378713

[B112] Iarrobino I. Bongiardina A. Dal Monte O. Sarasso P. Ronga I. Neppi-Modona M. . (2021). Right and left inferior frontal opercula are involved in discriminating angry and sad facial expressions. Brain Stimulat. 14, 607–615. 10.1016/j.brs.2021.03.014 33785407

[B113] Jalali A. Ziapour A. Karimi Z. Rezaei M. Emami B. Kalhori R. P. . (2024). Global prevalence of depression, anxiety, and stress in the elderly population: a systematic review and meta-analysis. BMC Geriatr. 24:809. 10.1186/s12877-024-05311-8 39367305 PMC11451041

[B114] James W. (1902). The Varieties of Religious Experience: a Study in Human Nature [Internet]. New York: Longmans Green and co. Available online at https://www.gutenberg.org/ebooks/621 (accessed March 5, 2025).

[B115] Jin C. Qi S. Yang L. Teng Y. Li C. Yao Y. . (2023). Abnormal functional connectivity density involvement in freezing of gait and its application for subtyping Parkinson's disease. Brain Imaging Behav. 17, 375–385. 10.1007/s11682-023-00765-7 37243751

[B116] Jin X. Zhong M. Yao S. Cao X. Tan C. Gan J. . (2016). A voxel-based morphometric MRI study in young adults with borderline personality disorder. PLoS ONE. 11:e0147938. 10.1371/journal.pone.0147938 26808504 PMC4726531

[B117] Kaisti I. Kulmala P. Hintsanen M. Hurtig T. Repo S. Paunio T. . (2024). The effects of mindfulness-based interventions in medical students: a systematic review. Adv Health Sci Educ. 29, 245–271. 10.1007/s10459-023-10231-0 37227541 PMC10927869

[B118] Kirkpatrick L. A. (2005). Attachment, Evolution, and the Psychology of Religion. New York, NY: The Guilford Press.

[B119] Kleshchova O. Rieder J. K. Grinband J. Weierich M. R. (2019). Resting amygdala connectivity and basal sympathetic tone as markers of chronic hypervigilance. Psychoneuroendocrinology 102, 68–78. 10.1016/j.psyneuen.2018.11.036 30529716 PMC6605037

[B120] Kobayashi D. First M. B. Shimbo T. Kanba S. Hirano Y. (2020). Association of self-reported religiosity with the development of major depression in multireligious country Japan. Psychiatry Clin. Neurosci. 74, 535–541. 10.1111/pcn.13087 32618044 PMC7586836

[B121] Kober S. E. Witte M. Ninaus M. Koschutnig K. Wiesen D. Zaiser G. . (2017). Ability to gain control over one's own brain activity and its relation to spiritual practice: a multimodal imaging study. Front. Hum. Neurosci. 11:271. 10.3389/fnhum.2017.00271 28596726 PMC5442174

[B122] Koenig H. G. (2007). Religion and depression in older medical inpatients. Am. J. Geriatr. Psychiatry. 15, 282–291. 10.1097/01.JGP.0000246875.93674.0c 17384313

[B123] Krause A. L. Borchardt V. Li M. van Tol M. J. Demenescu L. R. Strauss B. . (2016). Dismissing attachment characteristics dynamically modulate brain networks subserving social aversion. Front. Hum. Neurosci. 10:77. 10.3389/fnhum.2016.00077 27014016 PMC4783398

[B124] Kross E. Berman M. G. Mischel W. Smith E. E. Wager T. D. (2011). Social rejection shares somatosensory representations with physical pain. Proc. Natl. Acad. Sci. USA. 108, 6270–6275. 10.1073/pnas.1102693108 21444827 PMC3076808

[B125] Labek K. Viviani R. Gizewski E. R. Verius M. Buchheim A. (2016). Neural correlates of the appraisal of attachment scenes in healthy controls and social cognition—an fMRI study. Front. Hum. Neurosci. 10:345. 10.3389/fnhum.2016.00345 27458363 PMC4932100

[B126] Ladd K. L. Cook C. A. Foreman K. M. Ritter E. A. (2015). Neuroimaging of prayer: questions of validity. Psychol. Relig. Spiritual. 7, 100–108. 10.1037/a0039124

[B127] Laurita A. C. Hazan C. Spreng R. N. (2019). An attachment theoretical perspective for the neural representation of close others. Soc. Cogn. Affect. Neurosci. 14, 237–251. 10.1093/scan/nsz010 30715524 PMC6399606

[B128] Leighton J. S. (2021). The Effects of Different Types of Prayer on the Neurophysiological Functions among Evangelical Christians (dissertation on the Internet). Grand Canyon University, Phoenix, AZ, United States.

[B129] Leman J. Hunter W. Fergus T. Rowatt W. (2018). Secure attachment to God uniquely linked to psychological health in a national, random sample of American adults. Int. J. Psychol. Relig. 28, 162–173. 10.1080/10508619.2018.1477401

[B130] Lemche E. Giampietro V. P. Surguladze S. A. Amaro E. J. Andrew C. M. Williams S. C. R. . (2006). Human attachment security is mediated by the amygdala: evidence from combined fMRI and psychophysiological measures. Hum. Brain Mapp. 27, 623–635. 10.1002/hbm.20206 16284946 PMC6871466

[B131] Levy K. N. Meehan K. B. Kelly K. M. Reynoso J. S. Weber M. Clarkin J. F. . (2006). Change in attachment patterns and reflective function in a randomized control trial of transference-focused psychotherapy for borderline personality disorder. J. Consult. Clin. Psychol. 74, 1027–1040. 10.1037/0022-006X.74.6.1027 17154733

[B132] Li W. Mai X. Liu C. (2014). The default mode network and social understanding of others: what do brain connectivity studies tell us. Front. Hum. Neurosci. 8:74. 10.3389/fnhum.2014.00074 24605094 PMC3932552

[B133] Liu Y. Ding Y. Lu L. Chen X. (2017). Attention bias of avoidant individuals to attachment emotion pictures. Sci. Rep. 7:41631. 10.1038/srep41631 28128347 PMC5269715

[B134] Long M. Verbeke W. Ein-Dor T. Vrticka P. A. (2020). functional neuro-anatomical model of human attachment (NAMA): insights from first- and second-person social neuroscience. Cortex. 126, 281–321. 10.1016/j.cortex.2020.01.010 32092496

[B135] Lotze M. (2024). Emotional processing impairments in patients with insula lesions following stroke. Neuroimage. 291:120591. 10.1016/j.neuroimage.2024.120591 38552812

[B136] Lucchetti G. Koenig H. G. Lucchetti A. L. G. (2021). Spirituality, religiousness, and mental health: a review of the current scientific evidence. World J. Clin. Cases 9, 7620–7631. 10.12998/wjcc.v9.i26.7620 34621814 PMC8462234

[B137] Lyons-Ruth K. Pechtel P. Yoon S. A. Anderson C. M. Teicher M. H. (2016). Disorganized attachment in infancy predicts greater amygdala volume in adulthood. Behav. Brain Res. 308, 83–93. 10.1016/j.bbr.2016.03.050 27060720 PMC5017306

[B138] Maier M. A. Bernier A. Pekrun R. Zimmermann P. Grossmann K. E. (2004). Attachment working models as unconscious structures: An experimental test. Int. J. Behav. Dev. 28, 180–189. 10.1080/01650250344000398

[B139] Main M. (1991). “Metacognitive knowledge, metacognitive monitoring, and singular (coherent) vs. multiple (incoherent) model of attachment: findings and directions for future research,” in Attachment Across the Life Cycle, eds. P. Harris, J. Stevenson-Hinde, and C. Parkes (New York, NY, US: Tavistock/Routledge), 127–59.

[B140] McCullough M. E. Carter E. C. (2013). “Religion, self-control, and self-regulation: how and why are they related?,” in APA Handbook of Psychology, Religion, and Spirituality (Vol 1): Context, Theory, and Research (Washington, DC, US: American Psychological Association), 123–38.

[B141] McIntosh A. R. Rajah M. N. Lobaugh N. J. (1999). Interactions of prefrontal cortex in relation to awareness in sensory learning. Science 284, 1531–1533. 10.1126/science.284.5419.1531 10348741

[B142] McNamara P. (2009). The Neuroscience of Religious Experience. Cambridge: Cambridge University Press.

[B143] Meisenhelder J. Chandler E. (2002). Frequency of prayer and functional health in Presbyterian pastors. J. Sci. Study Relig. 40, 323–330. 10.1111/0021-8294.00059

[B144] Meisenhelder J. B. Chandler E. N. (2000). Prayer and health outcomes in church lay leaders. West. J. Nurs. Res. 22, 706–716. 10.1177/01939450022044692 11094574

[B145] Menon V. Uddin L. Q. (2010). Saliency, switching, attention and control: a network model of insula function. Brain Struct. Funct. 214, 655–667. 10.1007/s00429-010-0262-0 20512370 PMC2899886

[B146] Mikulincer M. Shaver P. R. (2003). “The attachment behavioral system in adulthood: activation, psychodynamics, and interpersonal processes,” in Advances in Experimental Social Psychology, ed. Z. P. Zanna (San Diego, CA, US: Elsevier Academic Press), 53–152.

[B147] Modinos G. Ormel J. Aleman A. (2009). Activation of anterior insula during self-reflection. PLoS ONE. 4:e4618. 10.1371/journal.pone.0004618 19242539 PMC2643476

[B148] Molenberghs P. Johnson H. Henry J. D. Mattingley J. B. (2016). Understanding the minds of others: a neuroimaging meta-analysis. Neurosci. Biobehav. Rev. 65, 276–291. 10.1016/j.neubiorev.2016.03.020 27073047

[B149] Molnar-Szakacs I. Uddin L. Q. (2022). Anterior insula as a gatekeeper of executive control. Neurosci. Biobehav. Rev. 139:104736. 10.1016/j.neubiorev.2022.104736 35700753

[B150] Moreira-Almeida A. Sharma A. van Rensburg B. J. Verhagen P. J. Cook C. C. H. (2016). WPA position statement on spirituality and religion in psychiatry. World Psychiatry. 15, 87–8. 10.1002/wps.20304 26833620 PMC4780301

[B151] Moutsiana C. Johnstone T. Murray L. Fearon P. Cooper P. J. Pliatsikas C. . (2015). Insecure attachment during infancy predicts greater amygdala volumes in early adulthood. J. Child Psychol. Psychiatry. 56, 540–548. 10.1111/jcpp.12317 25156392 PMC4407912

[B152] Müller-Pinzler L. Gazzola V. Keysers C. Sommer J. Jansen A. Frässle S. . (2015). Neural pathways of embarrassment and their modulation by social anxiety. Neuroimage 119, 252–261. 10.1016/j.neuroimage.2015.06.036 26093329 PMC5008438

[B153] Murphy S. T. Zajonc R. B. (1993). Affect, cognition, and awareness: Affective priming with optimal and suboptimal stimulus exposures. J. Pers. Soc. Psychol. 64, 723–739. 10.1037/0022-3514.64.5.723 8505704

[B154] Nash K. Prentice M. Hirsh J. McGregor I. Inzlicht M. (2014). Muted neural response to distress among securely attached people. Soc. Cogn. Affect. Neurosci. 9, 1239–1245. 10.1093/scan/nst099 23887815 PMC4127024

[B155] Nejati V. Majidinezhad M. Nitsche M. (2022). The role of the dorsolateral and ventromedial prefrontal cortex in emotion regulation in females with major depressive disorder (MDD): a tDCS study. J. Psychiatr. Res. 148, 149–158. 10.1016/j.jpsychires.2022.01.030 35124394

[B156] Neubauer R. L. (2014). Prayer as an interpersonal relationship: a neuroimaging study. Relig. Brain Behav. 4, 92–103. 10.1080/2153599X.2013.768288

[B157] Newberg A. (2018). Neurotheology: How Science can Enlighten us About Spirituality. New York, NY, US: Columbia University Press, 321.

[B158] Newberg A. B. (2014). The neuroscientific study of spiritual practices. Front. Psychol. 18:215. 10.3389/fpsyg.2014.00215 24672504 PMC3957224

[B159] Newberg A. B. Wintering N. A. Morgan D. Waldman M. R. (2006). The measurement of regional cerebral blood flow during glossolalia: a preliminary SPECT study. Psychiatry Res. Neuroimag. 148, 67–71. 10.1016/j.pscychresns.2006.07.001 17046214

[B160] Newman D. B. Nezlek J. B. Thrash T. M. (2023). The dynamics of prayer in daily life and implications for well-being. J. Pers. Soc. Psychol. 124, 1299–1313. 10.1037/pspp0000454 36622704 PMC10191893

[B161] Norenzayan A. Gervais W. M. Trzesniewski K. H. (2012). Mentalizing deficits constrain belief in a personal God. PLoS ONE 7:e36880. 10.1371/journal.pone.0036880 22666332 PMC3364254

[B162] Norman L. Lawrence N. Iles A. Benattayallah A. Karl A. (2015). Attachment-security priming attenuates amygdala activation to social and linguistic threat. Soc. Cogn. Affect. Neurosci. 10, 832–839. 10.1093/scan/nsu127 25326039 PMC4448028

[B163] Nowicki G. J. Schneider-Matyka D. Godlewska I. Tytuła A. Kotus M. Walec M. . (2023). The relationship between the strength of religious faith and spirituality in relation to post-traumatic growth among nurses caring for COVID-19 patients in eastern Poland: a cross-sectional study. Front. Psychiatry. 14:1331033. 10.3389/fpsyt.2023.1331033 38260777 PMC10800582

[B164] Ochsner K. N. Gross J. J. (2005). The cognitive control of emotion. Trends Cogn. Sci. 9, 242–249. 10.1016/j.tics.2005.03.010 15866151

[B165] O'Donnell L. J. Westin C. F. (2011). An introduction to diffusion tensor image analysis. Neurosurg. Clin. N. Am. 22:185. 10.1016/j.nec.2010.12.004 21435570 PMC3163395

[B166] Otten R. de Vries R. Schoonmade L. (2019). Deduplication of database search results for systematic reviews using EndNote. Zenodo. 10.5281/zenodo.3582928

[B167] Ouzzani M. Hammady H. Fedorowicz Z. Elmagarmid A. (2016). Rayyan—a web and mobile app for systematic reviews. Syst. Rev. 5:210. 10.1186/s13643-016-0384-4 27919275 PMC5139140

[B168] Page M. J. McKenzie J. E. Bossuyt P. M. Boutron I. Hoffmann T. C. Mulrow C. D. . (2021). The PRISMA 2020 statement: an updated guideline for reporting systematic reviews. BMJ 372:n71. 10.1136/bmj.n71 33782057 PMC8005924

[B169] Pargament K. I. (1997). The Psychology of Religion and Coping: Theory, Research, Practice. New York, NY, US: Guilford Press, 548.

[B170] Pargament K. I. (2007). Spiritually Integrated Psychotherapy: Understanding and Addressing the Sacred. New York: Guilford Press.

[B171] Pargament K. I. Ano G. G. Wachholtz A. B. (2005). “The religious dimension of coping: advances in theory, research, and practice,” in Handbook of the Psychology of Religion and Spirituality, ed. R. F. Paloutzian, C. L. Park (New York, NY, US: The Guilford Press), 479–495.

[B172] Pargament K. I. Hahn J. (1986). God and the just world: causal and coping attributions to God in health situations. J. Sci. Study Relig. 25, 193–207. 10.2307/1385476

[B173] Pargament K. I. Koenig H. G. Tarakeshwar N. Hahn J. (2004). Religious coping methods as predictors of psychological, physical, and spiritual outcomes among medically ill elderly patients: a two-year longitudinal study. J. Health Psychol. 9, 713–730. 10.1177/1359105304045366 15367751

[B174] Perez-Diaz O. Barrós-Loscertales A. Schjoedt U. González-Mora J. L. Rubia K. Suero J. . (2023). Monitoring the neural activity associated with praying in Sahaja Yoga meditation. BMC Neurosci. 24:61. 10.1186/s12868-023-00828-x 37957605 PMC10642040

[B175] Petrowski K. Wintermann G. B. Hübner T. Smolka M. N. Donix M. (2019). Neural responses to faces of attachment figures and unfamiliar faces: associations with organized and disorganized attachment representations. J. Nerv. Ment. Dis. 207, 112–120. 10.1097/NMD.0000000000000931 30688832

[B176] Picerni E. Laricchiuta D. Piras F. Petrosini L. Spalletta G. Cutuli D. . (2022). Cerebellar engagement in the attachment behavioral system. Sci. Rep. 12:13571. 10.1038/s41598-022-17722-x 35945247 PMC9363408

[B177] Porges S. W. (2022). Polyvagal theory: a science of safety. Front. Integr. Neurosci. 16:871227. 10.3389/fnint.2022.871227 35645742 PMC9131189

[B178] Quirin M. Gillath O. Pruessner J. C. Eggert L. D. (2010). Adult attachment insecurity and hippocampal cell density. Soc. Cogn. Affect. Neurosci. 5, 39–47. 10.1093/scan/nsp042 20007241 PMC2840841

[B179] Raccah O. Block N. Fox K. C. R. (2021). Does the prefrontal cortex play an essential role in consciousness? Insights from intracranial electrical stimulation of the human brain. J. Neurosci. 41, 2076–2087. 10.1523/JNEUROSCI.1141-20.2020 33692142 PMC8018764

[B180] Rajkumar R. P. A. (2021). biopsychosocial approach to understanding panic buying: integrating neurobiological, attachment-based, and social-anthropological perspectives. Front. Psychiatry. 12:652353. 10.3389/fpsyt.2021.652353 33716838 PMC7943846

[B181] Ravitz P. Maunder R. Hunter J. Sthankiya B. Lancee W. (2010). Adult attachment measures: a 25-year review. J. Psychosom. Res. 69, 419–432. 10.1016/j.jpsychores.2009.08.006 20846544

[B182] Redlich R. Grotegerd D. Opel N. Kaufmann C. Zwitserlood P. Kugel H. . (2015). Are you gonna leave me? Separation anxiety is associated with increased amygdala responsiveness and volume. Soc. Cogn. Affect. Neurosci. 10, 278–284. 10.1093/scan/nsu055 24752071 PMC4321627

[B183] Rigon A. Duff M. C. Voss M. W. (2016). Structural and functional neural correlates of self-reported attachment in healthy adults: evidence for an amygdalar involvement. Brain Imaging Behav. 10, 941–952. 10.1007/s11682-015-9446-9 26334650

[B184] Rognoni E. Galati D. Costa T. Crini M. (2008). Relationship between adult attachment patterns, emotional experience and EEG frontal asymmetry. Personal Individ Differ. 44, 909–920. 10.1016/j.paid.2007.10.021

[B185] Roisman G. I. Holland A. Fortuna K. Fraley R. C. Clausell E. Clarke A. . (2007). The Adult Attachment Interview and self-reports of attachment style: an empirical rapprochement. J. Pers. Soc. Psychol. 92, 678–697. 10.1037/0022-3514.92.4.678 17469952

[B186] Sakai Y. Kumano H. Nishikawa M. Sakano Y. Kaiya H. Imabayashi E. . (2005). Cerebral glucose metabolism associated with a fear network in panic disorder. Neuroreport. 16:927. 10.1097/00001756-200506210-00010 15931063

[B187] Schaap-Jonker H. (2020). Hebben psychiatrische patiënten wezenlijk andere godsrepresentaties dan niet-patiënten? Psyche. Geloof. 32, 19–32. Available online at: https://www.hannekeschaap.nl/media/PG_32_1_Hanneke%20Schaap-Jonker_2.pdf

[B188] Schaap-Jonker H. Corveleyn J. M. T. (2014). Mentalizing and religion: a promising combination for psychology of religion, illustrated by the case of prayer. Arch Psychol Relig. 36, 303–322. 10.1163/15736121-12341292

[B189] Schilbach L. Eickhoff S. B. Rotarska-Jagiela A. Fink G. R. Vogeley K. (2008). Minds at rest? Social cognition as the default mode of cognizing and its putative relationship to the “default system” of the brain. Conscious. Cogn. 17, 457–467. 10.1016/j.concog.2008.03.013 18434197

[B190] Schjødt U. Stødkilde-Jørgensen H. Geertz A. W. Roepstorff A. (2008). Rewarding prayers. Neurosci. Lett. 443, 165–168. 10.1016/j.neulet.2008.07.068 18682275

[B191] Schjoedt U. Stødkilde-Jørgensen H. Geertz A. W. Roepstorff A. (2009). Highly religious participants recruit areas of social cognition in personal prayer. Soc. Cogn. Affect. Neurosci. 4, 199–207. 10.1093/scan/nsn050 19246473 PMC2686228

[B192] Schurz M. Tholen M. G. Perner J. Mars R. B. Sallet J. (2017). Specifying the brain anatomy underlying temporo-parietal junction activations for theory of mind: a review using probabilistic atlases from different imaging modalities. Hum. Brain Mapp. 38, 4788–4805. 10.1002/hbm.23675 28608647 PMC6867045

[B193] Seibert J. (2023). The unity of religious experience: an analytic reading of Friedrich Schleiermacher's second speech on religion. Kriter – J Philos. 37, 123–145. 10.1515/krt-2023-0008

[B194] Serra M. De Pisapia N. Rigo P. Papinutto N. Jager J. Bornstein M. H. . (2015). Secure attachment status is associated with white matter integrity in healthy young adults. Neuroreport 26, 1106–1111. 10.1097/WNR.0000000000000479 26559724 PMC4646732

[B195] Shih H. C. Kuo M. E. Wu C. W. Chao Y. P. Huang H. W. Huang C. M. . (2022). The neurobiological basis of love: a meta-analysis of human functional neuroimaging studies of maternal and passionate love. Brain Sci. 12:830. 10.3390/brainsci12070830 35884637 PMC9313376

[B196] Silveira S. Bao Y. Wang L. Pöppel E. Avram M. Simmank F. . (2015). Does a bishop pray when he prays? And does his brain distinguish between different religions? PsyCh J. 4, 199–207. 10.1002/pchj.116 26663626

[B197] Šimić G. Tkalčić M. Vukić V. Mulc D. Španić E. Šagud M. . (2021). Understanding emotions: origins and roles of the amygdala. Biomolecules. 11, 823. 10.3390/biom11060823 34072960 PMC8228195

[B198] Simpson J. A. Steven Rholes W. (2017). Adult attachment, stress, and romantic relationships. Curr. Opin. Psychol. 13, 19–24. 10.1016/j.copsyc.2016.04.006 PMC4845754 27135049

[B199] Sinding Bentzen J. (2021). In crisis, we pray: religiosity and the COVID-19 pandemic. J. Econ. Behav. Organ. 192, 541–83. 10.1016/j.jebo.2021.10.014 34744223 PMC8557987

[B200] Solomon J. George C. (1999). “The place of disorganization in attachment theory: linking classic observations with contemporary findings,” in Attachment Disorganization, eds. J. Solomon, C. George (New York, NY, US: The Guilford Press), 3–32.

[B201] Solomon J. George C. (2008). “The measurement of attachment security and related constructs in infancy and early childhood,” in Handbook of Attachment: Theory, Research, and Clinical Applications, ed. J. Cassidy, and P. R. Shaver (New York, NY, US: The Guilford Press), 383–416.

[B202] Søvold L. E. Naslund J. A. Kousoulis A. A. Saxena S. Qoronfleh M. W. Grobler C. . (2021). Prioritizing the mental health and well-being of healthcare workers: an urgent global public health priority. Front Public Health 9:679397. 10.3389/fpubh.2021.679397 34026720 PMC8137852

[B203] Spilka B. Shaver P. Kirkpatrick L. A. A. (1985). general attribution theory for the psychology of religion. J. Sci. Study Relig. 24, 1–20. 10.2307/1386272

[B204] Stulp H. P. Koelen J. Glas G. G. Eurelings-Bontekoe L. (2021). Validation of the apperception test God representations, an implicit measure to assess God representations. Part 3: associations between implicit and explicit measures of God representations and self-reported level of personality functioning. J. Spiritual Ment. Health. 23, 197–219. 10.1080/19349637.2019.1700475

[B205] Surwillo W. W. Hobson D. P. (1978). Brain electrical activity during prayer. Psychol. Rep. 43, 135–143. 10.2466/pr0.1978.43.1.135 360259

[B206] Szałachowski R. R. Tuszyńska-Bogucka W. (2021). “Yes, in crisis we pray”. The role of prayer in coping with pandemic fears. Religions. 12:824. 10.3390/rel12100824

[B207] Takamura T. Nishitani S. Doi H. Shinohara K. (2016). Possible neural correlate of young child attachment to mother in 4 to 5 year olds. Acta Med. Nagasaki. 60, 45–52. 10.11343/amn.60.45

[B208] Takamura T. Nishitani S. Suegami T. Doi H. Shinohara K. (2015). Developmental changes in the neural responses to own and unfamiliar mother's smiling face throughout puberty. Front. Neurosci. 9:200. 10.3389/fnins.2015.00200 26089774 PMC4452823

[B209] Tang Y. Y. Hölzel B. K. Posner M. I. (2015). The neuroscience of mindfulness meditation. Nat. Rev. Neurosci. 16, 213–225. 10.1038/nrn3916 25783612

[B210] Tang Y. Y. Posner M. I. Rothbart M. K. (2014). Meditation improves self-regulation over the life span. Ann. N. Y. Acad. Sci. 1307, 104–111. 10.1111/nyas.12227 24033306 PMC4176767

[B211] Taylor P. Rietzschel J. Danquah A. Berry K. (2015). Changes in attachment representations during psychological therapy. Psychother. Res. J. Soc. Psychother. Res. 25, 222–238. 10.1080/10503307.2014.886791 24559454

[B212] Terasawa Y. Kurosaki Y. Ibata Y. Moriguchi Y. Umeda S. (2015). Attenuated sensitivity to the emotions of others by insular lesion. Front. Psychol. 6:1314. 10.3389/fpsyg.2015.01314 26388817 PMC4554943

[B213] Thomas J. Barbato M. (2020). Positive religious coping and mental health among Christians and Muslims in response to the COVID-19 pandemic. Religions 11:498. 10.3390/rel11100498

[B214] Tix A. P. Frazier P. A. (1998). The use of religious coping during stressful life events: main effects, moderation, and mediation. J. Consult. Clin. Psychol. 66, 411–422. 10.1037/0022-006X.66.2.411 9583344

[B215] Tricomi E. M. Delgado M. R. Fiez J. A. (2004). Modulation of caudate activity by action contingency. Neuron. 41, 281–292. 10.1016/S0896-6273(03)00848-1 14741108

[B216] Uddin L. Q. Nomi J. S. Hebert-Seropian B. Ghaziri J. Boucher O. (2017). Structure and function of the human insula. J. Clin. Neurophysiol. 34, 300–306. 10.1097/WNP.0000000000000377 28644199 PMC6032992

[B217] Upenieks L. (2023). Unpacking the relationship between prayer and anxiety: a consideration of prayer types and expectations in the United States. J. Relig. Health. 62, 1810–1831. 10.1007/s10943-022-01708-0 36449251 PMC9713100

[B218] Vadivel R. Shoib S. Halabi S. E. Hayek S. E. Essam L. Bytyçi D. G. . (2021). Mental health in the post-COVID-19 era: challenges and the way forward. Gen. Psychiatry. 34:e100424. 10.1136/gpsych-2020-100424 33644689 PMC7875255

[B219] van Elk M. Aleman A. (2017). Brain mechanisms in religion and spirituality: an integrative predictive processing framework. Neurosci. Biobehav. Rev. 73, 359–378. 10.1016/j.neubiorev.2016.12.031 28041787

[B220] van IJzendoorn M. H. Bakermans-Kranenburg M. J. (1996). Attachment representations in mothers, fathers, adolescents, and clinical groups: a meta-analytic search for normative data. J. Consult. Clin. Psychol. 64, 8–21. 10.1037/0022-006X.64.1.8 8907080

[B221] Van Overwalle F. Ma Q. Heleven E. (2020). The posterior crus II cerebellum is specialized for social mentalizing and emotional self-experiences: a meta-analysis. Soc. Cogn. Affect. Neurosci. 15, 905–928. 10.1093/scan/nsaa124 32888303 PMC7851889

[B222] Virto-Farfan H. Vargas D. Grajeda P. (2023). Dios mío, por qué me has abandonado? Estrés, ansiedad, depresión y afrontamiento religioso en los estudiantes de Medicina. Psiquiatr Biológica. 30:100427. 10.1016/j.psiq.2023.100427

[B223] Vos T. Allen C. Arora M. Barber R. M. Bhutta Z. A. Brown A. . (2016). Global, regional, and national incidence, prevalence, and years lived with disability for 310 diseases and injuries, 1990–2015: a systematic analysis for the Global Burden of Disease Study 2015. Lancet 388, 1545–1602. 10.1016/S0140-6736(16)31678-6 27733282 PMC5055577

[B224] Vrticka P. (2017). “The social neuroscience of attachment,” in Neuroscience and Social Science: The Missing Link, eds. A. L. Ibáñez, L. Sedeño, and A. M. García (Springer International Publishing/Springer Nature), 95–119. 10.1007/978-3-319-68421-5_5

[B225] Vrticka P. Andersson F. Grandjean D. Sander D. Vuilleumier P. (2008). Individual attachment style modulates human amygdala and striatum activation during social appraisal. PLoS ONE. 3:e2868. 10.1371/journal.pone.0002868 18682729 PMC2478709

[B226] Vrtička P. Bondolfi G. Sander D. Vuilleumier P. (2012). The neural substrates of social emotion perception and regulation are modulated by adult attachment style. Soc. Neurosci. 7, 473–493. 10.1080/17470919.2011.647410 22217336

[B227] Vrtička P. Sander D. Anderson B. Badoud D. Eliez S. Debban,é M. . (2014). Social feedback processing from early to late adolescence: influence of sex, age, and attachment style. Brain Behav. 4, 703–720. 10.1002/brb3.251 25328847 PMC4113975

[B228] Vrticka P. Simioni S. Fornari E. Schluep M. Vuilleumier P. Sander D. . (2013). Neural substrates of social emotion regulation: a fMRI study on imitation and expressive suppression to dynamic facial signals. Front. Psychol. 4:95. 10.3389/fpsyg.2013.00095 23450458 PMC3582997

[B229] Vrtička P. Vuilleumier P. (2012). Neuroscience of human social interactions and adult attachment style. Front. Hum. Neurosci. 6:212. 10.3389/fnhum.2012.00212 22822396 PMC3398354

[B230] Wachholtz A. B. Pargament K. I. (2005). Is spirituality a critical ingredient of meditation? comparing the effects of spiritual meditation, secular meditation, and relaxation on spiritual, psychological, cardiac, and pain outcomes. J. Behav. Med. 28, 369–384. 10.1007/s10865-005-9008-5 16049627

[B231] Walter Y. (2021). Neural structural changes associated with ritual glossolalia (a preliminary study): morphometrics on the expertise of praying in tongues. Soc. Sci. Res. Netw. 1–67. 10.2139/ssrn.3837534 [Epub ahead of print].

[B232] Weber S. R. Pargament K. I. (2014). The role of religion and spirituality in mental health. Curr. Opin. Psychiatry. 27, 358–363. 10.1097/YCO.0000000000000080 25046080

[B233] Weigle P. E. Shafi R. M. A. (2024). Social media and youth mental health. Curr. Psychiatry Rep. 26, 1–8. 10.1007/s11920-023-01478-w 38103128

[B234] White L. Kungl M. Vrticka P. (2023). Charting the social neuroscience of human attachment (SoNeAt). Attach. Hum. Dev. 25, 1–18. 10.1080/14616734.2023.2167777 36727628

[B235] White L. Schulz C. Schoett M. Kungl M. Keil J. Borelli J. . (2020). Conceptual analysis: a social neuroscience approach to interpersonal interaction in the context of disruption and disorganization of attachment (NAMDA). Front. Psychiatry. 11:517372. 10.3389/fpsyt.2020.517372 33424647 PMC7785824

[B236] White L. K. Makhoul W. Teferi M. Sheline Y. I. Balderston N. L. (2023). The role of dlPFC laterality in the expression and regulation of anxiety. Neuropharmacology 224:109355. 10.1016/j.neuropharm.2022.109355 36442650 PMC9790039

[B237] Winkeljohn Black S. Pössel P. Rosmarin D. H. Tariq A. Jeppsen B. D. (2017). Prayer type, disclosure, and mental health across religious groups. Couns. Values. 62, 216–234. 10.1002/cvj.12060

[B238] Wolfe F. H. Deruelle C. Chaminade T. (2018). Are friends really the family we choose? Local variations of hypothalamus activity when viewing personally known faces. Soc. Neurosci. 13, 289–300. 10.1080/17470919.2017.1317662 28388867

[B239] Wuthnow R. (2008). Prayer, cognition, and culture. Poetics 36, 333–337. 10.1016/j.poetic.2008.06.002

[B240] Yaseen Z. S. Zhang X. Muran J. C. Winston A. Galynker I. I. (2016). Comparison of brain activity correlating with self-report versus narrative attachment measures during conscious appraisal of an attachment figure. Front. Hum. Neurosci. 10:90. 10.3389/fnhum.2016.00090 27014022 PMC4789543

[B241] Zhang X. Deng M. Ran G. Tang Q. Xu W. Ma Y. . (2018a). Brain correlates of adult attachment style: a voxel-based morphometry study. Brain Res. 1699, 34–43. 10.1016/j.brainres.2018.06.035 29969580

[B242] Zhang X. Ran G. Xu W. Ma Y. Chen X. (2018b). Adult attachment affects neural response to preference-inferring in ambiguous scenarios: evidence from an fMRI study. Front. Psychol. 9:139. 10.3389/fpsyg.2018.00139 29559932 PMC5845741

[B243] Zhang X. Yaseen Z. S. Galynker I. I. Hirsch J. Winston A. (2011). Can depression be diagnosed by response to mother's face? A personalized attachment-based paradigm for diagnostic fMRI. PLoS ONE 6:e27253. 10.1371/journal.pone.0027253 22180777 PMC3236742

