# Supplementary material for: The convergent neuroscience of Christian prayer and attachment relationships in the context of mental health: a systematic review
Source: Front Psychol. 2025 Jun 18;16:1569514. doi: 10.3389/fpsyg.2025.1569514 (PMC12213507; doi:10.3389/fpsyg.2025.1569514)
Supplement: Supplementary file 1 [file Supplementary_file_1.docx]

Supplementary Table 1: Search Strategies in Databases

**Supplementary information: full search strategies**

**Search strategy for Elsevier/Scopus (March 25, 2024)**

| **8** | #7 AND NOT #5 | **23** |
| --- | --- | --- |
| **7** | #6 AND #4 | **24** |
| **6** | TITLE-ABS ("object relation*") OR AUTHKEY ("object relation*") | **7,958** |
| **5** | #3 AND #4 | **3,193** |
| **4** | TITLE-ABS (mri OR mris OR fmri OR fmris OR fnris OR "magnetic resonance" OR nirs OR "near infrared" OR neuroimag* OR neuro-imag* OR magnetic-encephalograph* OR magneto-encephalogr* OR magnetoencephalogr* OR meg) OR AUTHKEY (mri OR mris OR fmri OR fmris OR fnris OR "magnetic resonance" OR nirs OR "near infrared" OR neuroimag* OR neuro-imag* OR magnetic-encephalograph* OR magneto-encephalogr* OR magnetoencephalogr* OR meg) | **1,146,678** |
| **3** | #1 OR #2 | **267,362** |
| **2** | TITLE-ABS (pray*) OR AUTHKEY (pray*) | **20,313** |
| **1** | TITLE-ABS (attachment*) OR AUTHKEY (attachment*) | **247,154** |

**Search strategy for OVID/Medline (March 25, 2024)**

*Ovid MEDLINE(R) ALL <1946 to March 22, 2024>*

| **1** | exp "Object Attachment"/ or attachment*.ti,ab,kf. | **140047** |
| --- | --- | --- |
| **2** | pray*.ti,ab,kf. | **4052** |
| **3** | 1 or 2 | **144077** |
| **4** | exp "Magnetic Resonance Imaging"/ or exp "Magnetoencephalography"/ or "Neuroimaging"/ or "Functional Neuroimaging"/ or exp "Spectroscopy, Near-Infrared"/ or (mri or mris or fmri or fmris or fnris or "magnetic resonance" or nirs or "near infrared" or neuroimag* or neuro-imag* or magnetic-encephalograph* or magneto-encephalogr* or magnetoencephalogr* or meg).ti,ab,kf. | **948595** |
| **5** | 3 and 4 | **2866** |
| **6** | object-relation*.ti,ab,kf. | **1874** |
| **7** | 4 and 6 | **12** |
| **8** | 7 not 5 | **11** |

**Search strategy for Ebsco/ATLA Religion (March 25, 2024)**

| **#** | **Query** | **Results** |
| --- | --- | --- |
| **S7** | S4 AND S6 | **0** |
| **S6** | DE "Object relations (Psychoanalysis)" OR TI(object-relation*) OR AB(object-relation*) OR KW(object-relation*) | **203** |
| **S5** | S3 AND S4 | **4** |
| **S4** | DE "Brain -- Magnetic resonance imaging" OR TI(mri OR mris OR fmri OR fmris OR fnris OR "magnetic resonance" OR nirs OR "near infrared" OR neuroimag* OR neuro-imag* OR magnetic-encephalograph* OR magneto-encephalogr* OR magnetoencephalogr* OR meg) OR AB(mri OR mris OR fmri OR fmris OR fnris OR "magnetic resonance" OR nirs OR "near infrared" OR neuroimag* OR neuro-imag* OR magnetic-encephalograph* OR magneto-encephalogr* OR magnetoencephalogr* OR meg) OR KW(mri OR mris OR fmri OR fmris OR fnris OR "magnetic resonance" OR nirs OR "near infrared" OR neuroimag* OR neuro-imag* OR magnetic-encephalograph* OR magneto-encephalogr* OR magnetoencephalogr* OR meg) | **74** |
| **S3** | S1 OR S2 | **26,523** |
| **S2** | DE "Prayer" OR TI(pray*) OR AB(pray*) OR KW(pray*) | **25,907** |
| **S1** | DE "Attachment behavior" OR DE "Attachment theory" OR DE "Attachment behavior in infants" OR TI(attachment*) OR AB(attachment*) OR KW(attachment*) | **624** |

**Search strategy for Ebsco/CINAHL (March 25, 2024)**

| **#** | **Query** | **Results** |
| --- | --- | --- |
| **S7** | S4 AND S6 | **1** |
| **S6** | TI(object-relation*) OR AB(object-relation*) OR KW(object-relation*) | **342** |
| **S5** | S3 AND S4 | **521** |
| **S4** | MH "Magnetic Resonance Imaging" OR MH "Spectroscopy, Near-Infrared" OR MH "Neuroradiography" OR TI(mri OR mris OR fmri OR fmris OR fnris OR "magnetic resonance" OR nirs OR "near infrared" OR neuroimag* OR neuro-imag* OR magnetic-encephalograph* OR magneto-encephalogr* OR magnetoencephalogr* OR meg) OR AB(mri OR mris OR fmri OR fmris OR fnris OR "magnetic resonance" OR nirs OR "near infrared" OR neuroimag* OR neuro-imag* OR magnetic-encephalograph* OR magneto-encephalogr* OR magnetoencephalogr* OR meg) OR KW(mri OR mris OR fmri OR fmris OR fnris OR "magnetic resonance" OR nirs OR "near infrared" OR neuroimag* OR neuro-imag* OR magnetic-encephalograph* OR magneto-encephalogr* OR magnetoencephalogr* OR meg) | **209,458** |
| **S3** | S1 OR S2 | **28,626** |
| **S2** | MH "Prayer" OR TI(pray*) OR AB(pray*) OR KW(pray*) | **4,942** |
| **S1** | MH "Attachment Behavior+" OR TI(attachment*) OR AB(attachment*) OR KW(attachment*) | **23,713** |

**Search strategy for Ebsco/APA PsycINFO (March 25, 2024)**

| **#** | **Query** | **Results** |
| --- | --- | --- |
| **S8** | S7 NOT S5 | **26** |
| **S7** | S4 AND S6 | **27** |
| **S6** | DE "Object Relations" OR TI(object-relation*) OR AB(object-relation*) OR KW(object-relation*) | **12,059** |
| **S5** | S3 AND S4 | **396** |
| **S4** | DE "Functional Magnetic Resonance Imaging" OR DE "Magnetoencephalography" OR DE "Neuroimaging" OR TI(mri OR mris OR fmri OR fmris OR fnris OR "magnetic resonance" OR nirs OR "near infrared" OR neuroimag* OR neuro-imag* OR magnetic-encephalograph* OR magneto-encephalogr* OR magnetoencephalogr* OR meg) OR AB(mri OR mris OR fmri OR fmris OR fnris OR "magnetic resonance" OR nirs OR "near infrared" OR neuroimag* OR neuro-imag* OR magnetic-encephalograph* OR magneto-encephalogr* OR magnetoencephalogr* OR meg) OR KW(mri OR mris OR fmri OR fmris OR fnris OR "magnetic resonance" OR nirs OR "near infrared" OR neuroimag* OR neuro-imag* OR magnetic-encephalograph* OR magneto-encephalogr* OR magnetoencephalogr* OR meg) | **134,993** |
| **S3** | S1 OR S2 | **56,459** |
| **S2** | DE "Prayer" OR TI(pray*) OR AB(pray*) OR KW(pray*) | **4,664** |
| **S1** | DE "Attachment Style" OR DE "Attachment Theory" OR DE "Attachment Behavior" OR TI(attachment*) OR AB(attachment*) OR KW(attachment*) | **51,874** |

**Search strategy for Ebsco/Psychology and Behavioral Sciences Collection (March 25, 2024)**

DE "ATTACHMENT behavior in infants" OR DE "ATTACHMENT behavior" OR DE "ATTACHMENT behavior in children" OR DE "SIBLING attachment" OR DE "ADULT Attachment Interview" OR DE "ATTACHMENT theory (Psychology)" OR TI(attachment*) OR AB(attachment*) OR KW(attachment*)

**OR**

DE "PRAYER in Christianity" OR DE "PRAYER" OR DE "PRAYER groups" OR TI(pray*) OR AB(pray*) OR KW(pray*)

**AND**

DE "MAGNETIC resonance imaging evaluation" OR DE "MAGNETIC resonance imaging" OR DE "CONTRAST-enhanced magnetic resonance imaging" OR DE "DIFFUSION magnetic resonance imaging" OR DE "FUNCTIONAL magnetic resonance imaging" OR DE "MAGNETIC resonance imaging of the brain" OR DE "PHASE contrast magnetic resonance imaging" OR DE "NUCLEAR magnetic resonance" OR DE "MAGNETOENCEPHALOGRAPHY" OR DE "BRAIN imaging" OR DE "INFRARED spectroscopy techniques" OR DE "INFRARED imaging" OR DE "NEAR infrared spectroscopy" OR DE "NEAR infrared reflectance spectroscopy" OR TI(mri OR mris OR fmri OR fmris OR fnris OR "magnetic resonance" OR nirs OR "near infrared" OR neuroimag* OR neuro-imag* OR magnetic-encephalograph* OR magneto-encephalogr* OR magnetoencephalogr* OR meg) OR AB(mri OR mris OR fmri OR fmris OR fnris OR "magnetic resonance" OR nirs OR "near infrared" OR neuroimag* OR neuro-imag* OR magnetic-encephalograph* OR magneto-encephalogr* OR magnetoencephalogr* OR meg) OR KW(mri OR mris OR fmri OR fmris OR fnris OR "magnetic resonance" OR nirs OR "near infrared" OR neuroimag* OR neuro-imag* OR magnetic-encephalograph* OR magneto-encephalogr* OR magnetoencephalogr* OR meg)

DE "OBJECT relations" OR TI(object-relation*) OR AB(object-relation*) OR KW(object-relation*)

**Search strategy for PubPsych.eu (March 25, 2024)**

(attachment* OR pray*) AND (mri OR mris OR fmri OR fmris OR fnris OR "magnetic resonance" OR nirs OR "near infrared" OR neuroimag* OR neuro-imag* OR magnetic-encephalograph* OR magneto-encephalogr* OR magnetoencephalogr* OR meg)

**OR**

(object-relation*) AND (mri OR mris OR fmri OR fmris OR fnris OR "magnetic resonance" OR nirs OR "near infrared" OR neuroimag* OR neuro-imag* OR magnetic-encephalograph* OR magneto-encephalogr* OR magnetoencephalogr* OR meg)

**Search strategy for Clarivate Analytics/Web of Science Core Collection (March 25, 2024) 3354 attachment; 18 sample object)**

| **10** | #9 AND #4 | **18** |
| --- | --- | --- |
| **9** | TS=("object-relation*") | **4,435** |
| **5** | #4 AND #3 | **3,354** |
| **4** | TS=(mri OR mris OR fmri OR fmris OR fnris OR "magnetic resonance" OR nirs OR "near infrared" OR neuroimag* OR neuro-imag* OR magnetic-encephalograph* OR magneto-encephalogr* OR magnetoencephalogr* OR meg) | **1,078,227** |
| **3** | #2 OR #1 | **241,127** |
| **2** | TS=(pray*) | **18,316** |
| **1** | TS=(attachment*) | **222,921** |

Supplementary File 1: Screening Process

Interrater agreement was 98 percent, with a weighted Cohen's Kappa of 0,30 indicating fair agreement. One of the reasons for the lower than expected Cohen’s Kappa, was a difference between raters attitude towards the screening protocol. One of the screeners included records that potentially reported the findings as specified according to the PICOS-criteria in the full article, even when they were not clearly mentioned in title or abstract, whereas another author excluded records that did not concur with the screening protocol based on title and abstract only. The discrepancy is not estimated to have influenced the included number of records, as the first screener included a significantly larger number of studies that considerably expanded the range of articles that were fully screened by both authors.

Supplementary Table 2-7: Critical Appraisal of Included Studies

*Table 2. Quality Assessment of Studies that Implicitly Assessed Attachment*

| **Author, year** | **Score out of 10** | **Sample size (+)** | **Criteria for inclusion (+) & subject settings (+)** | **Exposure condition (++)** | **Attachment assessment**  **(+)** | **Confounding factors identified and adjusted via a reliable method (+)** | **Outcomes reliably measured (+)** | **Appropriate statistical analysis (+) and findings corrected for multiple comparisons (+)** |
| --- | --- | --- | --- | --- | --- | --- | --- | --- |
| [**Buchheim et al. 2006**](https://doi.org/10.1159/000091800) | 8/10 | - | ++ | ++ | + | + | + | +- |
| [**Lemche et al. 2005**](https://doi.org/10.1002/hbm.20206) | 8/10 | - | ++ | +- | + | + | + | ++ |
| [**Petrowski et al. 2019**](https://doi.org/10.1097/NMD.0000000000000931) | 9/10 | + | ++ | +- | + | + | + | ++ |
| [**Yaseen et al. 2016**](http://dx.doi.org/10.3389/fnhum.2016.00090) | 9/10 | + | ++ | ++ | + | - | + | ++ |

*Table 3. Quality Assessment of Studies that Implicitly Assessed Attachment with Comorbidity*

| **Author, year** | **Score out of 10** | **Sample size (+)** | **Criteria for inclusion (+) & subject settings (+)** | **Exposure condition (++)** | **Attachment assessment**  **(+)** | **Confounding factors identified and adjusted via a reliable method (+)** | **Outcomes reliably measured (+)** | **Appropriate statistical analysis (+) and findings corrected for multiple comparisons (+)** |
| --- | --- | --- | --- | --- | --- | --- | --- | --- |
| [**Bernheim et al. 2022**](https://doi.org/10.3389/fnhum.2022.810417) | 9/10 | + | ++ | ++ | + | - | + | ++ |
| [**Buchheim et al. 2008**](https://doi.org/10.1016/j.pscychresns.2007.07.001) | 9/10 | - | ++ | ++ | + | +  (Participants matched for age and education. All female) | + | ++  (Only main effects were uncorrected) |
| [**Buchheim et al. 2012a**](https://doi.org/10.1371/journal.pone.0033745) | 8/10 | - | ++ | ++ | + | + | + | +- |
| [**Buchheim et al. 2012b**](https://doi.org/10.1007/s00278-012-0909-9) | 8/10 | - | ++ | ++ | + | +  (Participantsmatched for age, education and gender) | + | +- |
| [**Buchheim et al. 2013**](http://www.frontiersin.org/Human_Neuroscience/editorialboard) | 9/10 | - | ++ | ++ | + | + | + | ++ |
| [**Buchheim et al. 2016**](http://dx.doi.org/10.3389/fnhum.2016.00389) | 9/10 | - | ++ | ++ | + | +  (Participants matched for age and education. All female) | + | ++ |
| [**Flechsig et al. 2023**](https://doi.org/10.3390/brainsci13071001) | 8.5/10 | +/- | ++ | ++ | + | - | + | ++ |
| [**Galynker et al. 2012**](https://doi.org/10.1093/scan/nsr074) | 10/10 | + | ++ | ++ | + | + | + | ++ |
| [**Zhang et al. 2011**](https://doi.org/10.3389/fnhum.2022.810417) | 9/10 | + | ++ | ++ | + | + | + | +- |

*Table 4. Quality Assessment of Studies that Explicitly Assessed Attachment*

| **Author, year** | **Score out of 10** | **Sample size (+)** | **Criteria for inclusion (+) & subject settings (+)** | **Exposure condition (++)** | **Attachment assessment**  **(+)** | **Confounding factors identified and adjusted via a reliable method (+)** | **Outcomes reliably measured (+)** | **Appropriate statistical analysis (+) and findings corrected for multiple comparisons (+)** |
| --- | --- | --- | --- | --- | --- | --- | --- | --- |
| [**Canterberry et al. 2013**](http://dx.doi.org/10.1016/j.ijpsycho.2012.08.013) | 8/10 | + | +- | ++ | + | + | + | +- |
| [**Krause et al. 2016**](https://doi.org/10.3389/fnhum.2016.00077) | 7.5/10 | + | ++ | +- | + | + | + | ++/- |
| [**Liu et al. 2017**](https://doi.org/10.1038/srep41631) | 7/10 | - | ++ | +- | + | - | + | ++ |
| [**Nash et al. 2014**](https://doi.org/10.1093/scan/nst099) | 6/10 | + | -+ | ++ | + | - | + | – |
| [**Pascal Vrtička et al. 2012**](https://doi.org/10.1080/17470919.2011.647410) | 8/10 | - | ++ | +- | + | + | + | ++ |
| [**Elena rognoni et al. 2007**](https://doi.org/10.1016/j.paid.2007.10.021) | 7/10 | - | +- | +- | + | + | + | ++ |
| [**Yaseen et al. 2016**](http://dx.doi.org/10.3389/fnhum.2016.00090)***** | 28  (F = 28) | + | +- | ++ | + | - | + | ++ |

*This study was also mentioned in*‘Table 2. Quality Assessment of Studies that Implicitly Assessed Attachment’*, but is here evaluated for its results with self-report questionnaires.

*Table 5. Quality Assessment of Studies Reporting Structural Findings of Attachment*

| **Author, year** | **Score out of 10** | **Sample size (+)** | **Criteria for inclusion (+) & subject settings (+)** | **Method of analysis specified (++)** | **Attachment assessment**  **(+)** | **Confounding factors identified and adjusted via a reliable method (+)** | **Outcomes reliably measured (+)** | **Appropriate statistical analysis (+) and findings corrected for multiple comparisons (+)** |
| --- | --- | --- | --- | --- | --- | --- | --- | --- |
| [**Acosta et al. 2018**](https://doi.org/10.1016/j.neuroscience.2018.06.045) | 10/10 | + | ++ | ++ | + | + | + | ++ |
| [**Benetti et al. 2010**](https://doi.org/10.1002/hbm.20954) | 9/10 | + | +- | ++ | + | + | + | ++ |
| [**Bracht et al. 2022**](https://doi.org/10.1016/j.brainres.2018.06.035) | 10/10 | + | ++ | ++ | + | + | + | ++ |
| [**Jin et al. 2016**](https://doi.org/10.1371/journal.pone.0147938) | 10/10 | + | ++ | ++ | + | + | + | ++ |
| [**Lyons-Ruth et al. 2016**](https://doi.org/10.1016/j.bbr.2016.03.050)***** | 8/10 | - | ++ | ++ | + | + | + | +- |
| [**Moutsiana et al. 2014**](https://doi.org/10.1111/jcpp.12317)****** | 9/10 | + | ++ | ++ | + | + | + | +- |
| [**Picerni et al. 2022**](https://doi.org/10.1038/s41598-022-17722-x) | 10/10 | + | ++ | ++ | + | + | + | ++ |
| [**Quirin et al. 2010**](https://doi.org/10.1093/scan/nsp042) | 10/10 | + | ++ | ++ | + | + | + | ++ |
| [**Redlich et al. 2015**](https://doi.org/doi:10.1093/scan/nsu055) | 9/10 | + | ++ | ++ | + | - | + | ++ |
| [**Rigon et al. 2016**](https://doi.org/10.1007/s11682-015-9446-9) | 10/10 | + | ++ | ++ | + | + | + | ++ |
| [**Serra et al. 2015**](https://doi.org/10.1097/WNR.0000000000000479) | 8/10 | + | ++ | ++. | + | - | + | +- |
| [**Zhang et al. 2018**](https://doi.org/10.1016/j.brainres.2018.06.035) | 10/10 | + | ++ | ++ | + | + | + | ++ |

*Table 6. Quality Assessment of Studies on Christian Prayer*

| **Author, year** | **Score out of 9** | **Sample size (+)** | **Criteria for inclusion (+) & subject settings (+)** | **Exposure condition (++)** | **Control condition other than baseline activity (+)** | **Confounding factors identified and adjusted via a reliable method (+)** | **Outcomes reliably measured (+)** | **Appropriate statistical analysis (+) and findings corrected for multiple comparisons (+)** |
| --- | --- | --- | --- | --- | --- | --- | --- | --- |
| [**Azari et al. 2001**](https://www.academia.edu/48886577/Neural_correlates_of_religious_experience) | 6/10 | - | ++ | +- | + | - | + | +- |
| [**Azari et al. 2005**](https://doi.org/10.1207/s15327582ijpr1504_1) | 7/10 | - | ++ | +- | + | + | + | +- |
| [**Elmholdt et al. 2017**](https://doi.org/10.3389/fnhum.2017.00337) | 9/10 | + | ++ | ++ | + | - | + | ++ |
| [**Beauregard and Paquette, 2006**](https://doi.org/10.1016/j.neulet.2006.06.060) | 7/10 | - | ++ | ++ | + | - | + | +- |
| [**Beauregard and Paquette, 2008**](https://doi.org/10.1016/j.neulet.2008.08.028) | 7/10 | - | ++ | ++ | + | - | + | +- |
| [**Galanter et al. 2017**](https://doi.org/10.3109/00952990.2016.1141912) | 9/10 | - | ++ | ++ | + | + | + | ++ |
| [**Kober et al. 2017**](https://doi.org/10.3389/fnhum.2017.00271) | 8/10 | + | ++ | – | + | + | + | ++ |
| [**Leighton, 2021**](https://www.proquest.com/openview/d7c8528fec440213562b928dfe5b2f44/1?pq-origsite=gscholar&cbl=18750&diss=y) | 7/10 | + | ++ | ++ | - | - | + | +- |
| [**Neubauer et al. 2014**](https://doi.org/10.1080/2153599X.2013.768288) | 8/10 | - | ++ | ++ | + | - | + | ++ |
| [**Newberg et al. 2003**](https://doi.org/10.2466/pms.2003.97.2.625)***** | 4/10 | - | – | ++ | - | - | + | +- |
| [**Schjødt et al. 2008**](https://doi.org/10.1016/j.neulet.2008.07.068) | 9/10 | + | ++ | ++ | + | - | + | ++ |
| [**Schjoedt et al. 2009**](https://doi.org/10.1093/scan/nsn050) | 9/10 | + | ++ | ++ | + | - | + | ++ |
| [**Silveira et al. 2015**](https://doi.org/10.1002/pchj.116) | 6/10 | - | ++ | ++ | - | - | + | +- |
| [**Surwillo and Hobson, 1978**](https://doi.org/10.2466/pr0.1978.43.1.135) | 6/10 | - | ++ | ++ | - | - | + | +- |

*This study was excluded based on the quality assessment and fit with our research question.

*Table 7. Quality Assessment of Structural Neuroimaging Study on Praye*r

| **Author, year** | **Score out of 10** | **Sample size (+)** | **Criteria for inclusion (+) & subject settings (+)** | **Method of analysis specified (++)** | **Control condition other than baseline activity**  **(+)** | **Confounding factors identified and adjusted via a reliable method (+)** | **Outcomes reliably measured (+)** | **Appropriate statistical analysis (+) and findings corrected for multiple comparisons (+)** |
| --- | --- | --- | --- | --- | --- | --- | --- | --- |
| [**Kober et al. 2017**](https://doi.org/10.3389/fnhum.2017.00271)***** | 10/10 | + | ++ | ++ | + | + | + | ++ |

*This study occurs in table 6 as well as both structural and functional neuroimaging results were reported.

Supplementary file 2: Critical Appraisal Tool

**Analytical cross sectional studies Critical Appraisal Tool, adapted for a study on the convergent neuroscience of Christian prayer and attachment relationships: a systematic review**

Original source: Moola S, Munn Z, Tufanaru C, Aromataris E, Sears K, Sfetcu R, Currie M, Qureshi R, Mattis P, Lisy K, Mu P-F. Chapter 7: Systematic reviews of etiology and risk . In: Aromataris E, Munn Z (Editors). JBI Manual for Evidence Synthesis. JBI, 2020. Available from <https://synthesismanual.jbi.global>

Answers: (+) / (-)

1. Was the sample size sufficient? (+)

Sample size was justified and satisfactory. At least 20 participants per group were analysed. (+) Conversely, either the inclusion/exclusion criteria were not justified, the sample size was not satisfactory or less than 20 participants per group were analysed. (-)

2. Were the criteria for inclusion in the sample clearly defined, and were study subjects and the setting described in detail? (++)

The authors should provide clear inclusion and exclusion criteria that they developed prior to recruitment of the participants. The inclusion/exclusion criteria should be specified (e.g. neurological diseases, mental health condition, age and used measurement tools) with sufficient detail and all the necessary information critical to the study. (+) Furthermore, the study sample should be described in sufficient detail so that other researchers can determine if it is comparable to the population of interest to them. The authors should provide a clear description of the population from which the study participants were selected or recruited, including demographics, location, mean age, years of education and/or socio-economic status. If the study is longitudinal, the time period should be described in sufficient detail. (+)

3. Did the exposure measure reliably activate the attachment system? (++)

The study should clearly describe the method of measurement of exposure. The exposure condition in attachment-related studies should be specifically designed to activate the attachment system instead of the affiliative, parental or romantic system. (++) If the exposure condition measures the attachment condition in combination with the affiliative, parental or sexual system so that results are confounded (-). For studies on prayer, the exposure condition consists of any form of prayer towards God (++), whether it be ritual, colloquial, meditative or formalized prayer. However, if reading a biblical text out loud is the prayer condition, assign only one +. (+-) In structural neuroimaging studies, this question was replaced by whether the method of analysis was specified (VBM, DTI), if so. (++) if not or unclear. (--)

4. Were objective, standard criteria used for measurement of the condition? (+)

Assessing validity requires that a 'gold standard' is available to which the measure can be compared. The validity of exposure measurement usually relates to whether a current measure is appropriate or whether a measure of past exposure is needed. The condition was measured with a validated measure that has been shown to activate the attachment system in previous or later studies. (+) If a new method is employed that is not yet validated, even though it is designed to activate the attachment system. (-) For studies related to prayer, this category is replaced by whether a good control condition has been used. (+)

5. Were confounding factors identified, and were strategies to deal with confounding factors applied? (+)

Confounding has occurred where the estimated intervention exposure effect is biased by the presence of some difference between the comparison groups (apart from the exposure investigated/of interest). Typical confounders include baseline characteristics, prognostic factors, or mental health conditions (Depression/Anxiety). A confounder is a difference between the comparison groups and it influences the direction of the study results. A high quality study at the level of cohort design will identify the potential confounders and measure them (where possible). This is difficult for studies where behavioral, attitudinal or lifestyle factors may impact on the results.

Confounders were identified, such as sex of participants, state anxiety prior to the scans or any other confounder. Furthermore, strategies to deal with effects of confounding factors have been applied in the study design or in data analysis. By matching or stratifying sampling of participants, effects of confounding factors can be adjusted for. When dealing with adjustment in data analysis, assess the statistics used in the study. Most will be some form of multivariate regression analysis to account for the confounding factors measured. Not only were confounding factors identified, but they were also incorporated into the analysis to mitigate bias. (+) Conversely, if confounders were not identified, or their effects were acknowledged but not addressed, this was noted as a limitation. (-)

6. Were the outcomes measured in a valid and reliable way? (+)

Read the methods section of the paper. If for e.g. attachment style is assessed based on existing definitions or diagnostic criteria, then the answer to this question is likely to be yes. If attachment is assessed using observer reported, or self-reported scales, the risk of over- or under-reporting is increased, and objectivity is compromised. Importantly, determine if the measurement tools used were validated instruments as this has a significant impact on outcome assessment validity.

Having established the objectivity of the outcome measurement (e.g. attachment or prayer) instrument, it’s important to establish how the measurement was conducted. Were those involved in collecting data trained or educated in the use of the instrument/s? (e.g. reliable coders of the AAP). If there was more than one data collector, were they similar in terms of level of education, clinical or research experience, or level of responsibility in the piece of research being appraised? How was attachment style assessed (AAP or self-report), or else, was belief in God a prerequisite for participation? Furthermore, were the necessary conditions in place for proper assessment? For the AAP this would mean that coding had been performed by reliable coders who were trained in accordance with AAP-guidelines. Furthermore, the neuroimaging scans must have been conducted in line with best-practices. Finally, the study should control for head movement and for baseline activity if no other control condition was specified. (+) if one of these criteria has not been met. (-)

7. Was appropriate statistical analysis used? (++)

As with any consideration of statistical analysis, consideration should be given to whether there was a more appropriate alternate statistical method that could have been used. The methods section should be detailed enough for reviewers to identify which analytical techniques were used (in particular, regression or stratification) and how specific confounders were measured. (+) For studies utilizing regression analysis, it is useful to identify if the study identified which variables were included and how they related to the outcome. If stratification was the analytical approach used, were the strata of analysis defined by the specified variables? Additionally, it is important to assess the appropriateness of the analytical strategy in terms of the assumptions associated with the approach as differing methods of analysis are based on differing assumptions about the data and how it will respond. Furthermore, identify whether the results were corrected for multiple comparisons (+). If one element was missing. (-)
